# *Pseudorhabdosynochus* species (Monogenoidea, Diplectanidae) parasitizing groupers (Serranidae, Epinephelinae, Epinephelini) in the western Atlantic Ocean and adjacent waters, with descriptions of 13 new species

**DOI:** 10.1051/parasite/2015024

**Published:** 2015-08-12

**Authors:** Delane C. Kritsky, Micah D. Bakenhaster, Douglas H. Adams

**Affiliations:** 1 Health Education Program, School of Health Professions, Campus Box 8090, Idaho State University Pocatello Idaho 83209 USA; 2 Fish and Wildlife Health Group, Fish and Wildlife Research Institute, Florida Fish and Wildlife Conservation Commission 100 8th Avenue Southeast St. Petersburg Florida 33701-5020 USA; 3 Florida Fish and Wildlife Conservation Commission, Fish and Wildlife Research Institute 1220 Prospect Avenue, No. 285 Melbourne Florida 32901 USA

**Keywords:** Monogenea, Diplectanidae, *Pseudorhabdosynochus*, Grouper, Serranidae, Epinephelini

## Abstract

Seventeen of twenty-three species of groupers collected from the western Atlantic Ocean and adjacent waters were infected with 19 identified species (13 new) of *Pseudorhabdosynochus* Yamaguti, 1958 (Dactylogyridea, Diplectanidae); specimens of the Spanish flag *Gonioplectrus hispanus*, coney *Cephalopholis fulva*, marbled grouper *Dermatolepis inermis*, mutton hamlet *Alphestes afer*, and misty grouper *Hyporthodus mystacinus* were not infected; the yellowmouth grouper *Mycteroperca interstitialis* and yellowfin grouper *Mycteroperca venenosa* were infected with unidentified species of *Pseudorhabdosynochus*; the Atlantic creolefish *Paranthias furcifer* was infected with an unidentified species of Diplectanidae that could not be accommodated in *Pseudorhabdosynochus*. The following species of *Pseudorhabdosynochus* are described or redescribed based entirely or in part on new collections: *Pseudorhabdosynochus americanus* (Price, 1937) Kritsky & Beverley-Burton, 1986 from Atlantic goliath grouper *Epinephelus itajara*; *Pseudorhabdosynochus yucatanensis* Vidal-Martínez, Aguirre-Macedo & Mendoza-Franco, 1997 and *Pseudorhabdosynochus justinella* n. sp. from red grouper *Epinephelus morio*; *Pseudorhabdosynochus kritskyi* Dyer, Williams & Bunkley-Williams, 1995 from gag *Mycteroperca microlepis*; *P*se*udorhabdosynochus capurroi* Vidal-Martínez & Mendoza-Franco, 1998 from black grouper *Mycteroperca bonaci*; *Pseudorhabdosynochus hyphessometochus* n. sp. from *Mycteroperca interstitialis*; *Pseudorhabdosynochus sulamericanus* Santos, Buchmann & Gibson, 2000 from snowy grouper *Hyporthodus niveatus* and Warsaw grouper *Hyporthodus nigritus* (new host record); *Pseudorhabdosynochus firmicoleatus* n. sp. from yellowedge grouper *Hyporthodus flavolimbatus* and snowy grouper *H. niveatus*; *Pseudorhabdosynochus mcmichaeli* n. sp., *Pseudorhabdosynochus contubernalis* n. sp., and *Pseudorhabdosynochus vascellum* n. sp. from scamp *Mycteroperca phenax*; *Pseudorhabdosynochus meganmarieae* n. sp. from graysby *Cephalopholis cruentata*; *Pseudorhabdosynochus beverleyburtonae* (Oliver, 1984) Kritsky & Beverley-Burton, 1986 from dusky grouper *Mycteroperca marginata*; *Pseudorhabdosynochus mizellei* n. sp. from red hind *Epinephelus guttatus*; *Pseudorhabdosynochus williamsi* n. sp. from rock hind *Epinephelus adscensionis*; *Pseudorhabdosynochus bunkleywilliamsae* n. sp. from Nassau grouper *Epinephelus striatus*; *Pseudorhabdosynochus mycteropercae* n. sp. from tiger grouper *Mycteroperca tigris*; and *Pseudorhabdosynochus tumeovagina* n. sp. from speckled hind *Epinephelus drummondhayi*. *Pseudorhabdosynochus woodi* n. sp. from red hind *Epinephelus guttatus* is described based on specimens from the US National Parasite Collection (USNPC). Drawings of the haptoral and copulatory sclerites of the type specimens in the USNPC of *Pseudorhabdosynochus monaensis* Dyer, Williams & Bunkley-Williams, 1994 from rock hind *Epinephelus adscensionis* are presented. Finally, a note confirming *Pseudorhabdosynochus epinepheli* Yamaguti, 1958 rather than its senior synonym *Pseudorhabdosynochus epinepheli* (Yamaguti, 1938) Kritsky & Beverley-Burton, 1986 as the type species of *Pseudorhabdosynochus* is provided.

## Abstract (fr)

Dix-sept des 23 espèces de mérous prélevées dans les eaux de l’océan Atlantique Ouest et adjacentes sont infectées avec 19 espèces identifiées (13 nouvelles) de *Pseudorhabdosynochus* Yamaguti, 1958 (Dactylogyridea, Diplectanidae) ; les spécimens de *Gonioplectrus hispanus*, *Cephalopholis fulva*, *Dermatolepis inermis*, *Alphestes afer* et *Hyporthodus mystacinus* n’étaient pas infectés ; *Mycteroperca interstitialis* et *Mycteroperca venenosa* étaient infectés par des espèces non identifiées de *Pseudorhabdosynochus* ; *Paranthias furcifer* était infecté par une espèce non identifiée de Diplectanidae qui ne correspond pas à *Pseudorhabdosynochus*. Les espèces suivantes de *Pseudorhabdosynochus* sont décrites ou redécrites, sur la base de nouvelles collections en tout ou pour partie : *Pseudorhabdosynochus americanus* (Price, 1937) Kritsky & Beverley-Burton, 1986, d’*Epinephelus itajara* ; *Pseudorhabdosynochus yucatanensis* Vidal-Martínez, Aguirre-Macedo & Mendoza-Franco, 1997 et *Pseudorhabdosynochus justinella* n. sp., d’*Epinephelus morio* ; *Pseudorhabdosynochus kritskyi* Dyer, Williams & Bunkley-Williams, 1995, de *Mycteroperca microlepis* ; *Pseudorhabdosynochus capurroi* Vidal-Martínez & Mendoza-Franco, 1998, de *Mycteroperca bonaci* ; *Pseudorhabdosynochus hyphessometochus* n. sp. de *Mycteroperca interstitialis* ; *Pseudorhabdosynochus sulamericanus* Santos, Buchmann & Gibson, 2000, d’*Hyporthodus niveatus* et *Hyporthodus nigritus* (nouvelle mention d’hôte) ; *Pseudorhabdosynochus firmicoleatus* n. sp., d’*Hyporthodus flavolimbatus* et *H. niveatus* ; *Pseudorhabdosynochus mcmichaeli* n. sp., *Pseudorhabdosynochus contubernalis* n. sp. et *Pseudorhabdosynochus vascellum* n. sp., de *Mycteroperca phenax* ; *Pseudorhabdosynochus meganmarieae* n. sp., de *Cephalopholis cruentata* ; *Pseudorhabdosynochus beverleyburtonae* (Oliver, 1984) Kritsky & Beverley-Burton, 1986, de *Mycteroperca marginata* ; *Pseudorhabdosynochus mizellei* n. sp., d’*Epinephelus guttatus* ; *Pseudorhabdosynochus williamsi* n. sp., d’*Epinephelus adscensionis* ; *Pseudorhabdosynochus bunkleywilliamsae* n. sp., d’*Epinephelus striatus* ; *Pseudorhabdosynochus mycteropercae* n. sp., de *Mycteroperca tigris* ; *Pseudorhabdosynochus tumeovagina* n. sp., d’*Epinephelus drummondhayi*. *Pseudorhabdosynochus woodi* n. sp., d’*Epinephelus guttatus*, est décrit à partir de spécimens de la collection nationale de parasites des États-Unis (USNPC). Des dessins des sclérites haptoraux et copulatoires des spécimens types de l’USNPC de *Pseudorhabdosynochus monaensis* Dyer, Williams & Bunkley-Williams, 1994, d’*Epinephelus adscensionis,* sont présentés. Enfin, une note confirmant *Pseudorhabdosynochus epinepheli* Yamaguti, 1958 plutôt que son synonyme *Pseudorhabdosynochus epinepheli* (Yamaguti, 1938) Kritsky & Beverley-Burton, 1986 comme espèce-type de *Pseudorhabdosynochus* est fournie.

## Introduction

*Pseudorhabdosynochus* Yamaguti, 1958 (Monogenoidea: Dactylogyridea: Diplectanidae) was proposed, with the new species *Pseudorhabdosynochus epinepheli* Yamaguti, 1958 collected from the gills of Hong Kong grouper *Epinephelus akaara* (Temminck & Schlegel) from the Inland Sea of Japan, assigned as its type species by Yamaguti [57]. The genus was characterized in part by species (*P. epinepheli*) having unarmed squamodiscs and an intercecal germarium. Twenty years earlier, Yamaguti [55] had described *Diplectanum epinepheli* Yamaguti, 1938 from *E. akaara*; this species was based partly on the presence of armed haptoral squamodiscs and an intercecal germarium. The two species were subsequently placed in synonymy by Kritsky & Beverley-Burton [25], who, upon examination of the type specimens, found that the germarium looped the right intestinal cecum dorsoventrally in both nominal species; they also stated that absence of an armed squamodisc in *P. epinepheli* was not sufficient to exclude it from the complex of species with armed squamodiscs because the rodlets of the squamodiscs are easily lost if fixation is not done shortly after death of the diplectanid. Finally, Kritsky & Beverley-Burton [25] transferred 13 species of *Cycloplectanum* Oliver, 1968 to *Pseudorhabdosynochus* after determining that *Cycloplectanum* was its junior synonym.

*Pseudorhabdosynochus* currently contains approximately 80 valid species [13], all with the exception of three, reported from groupers assigned to the serranid tribe Epinephelini, members of which support some of the most valuable marine fisheries in the tropical and subtropical regions of the world. The only species recorded exclusively from non-epinephelin hosts are *Pseudorhabdosynochus magnisquamodiscum* (Aljoshkina, 1984) Dyer, Williams & Bunkley-Williams, 1995 from the chaetodontid *Chaetodon hoefleri* Steindachner in the southeastern Atlantic Ocean [1], *Pseudorhabdosynochus serrani* (Yamaguti, 1953) Kritsky & Beverley-Burton, 1986 from an unidentified species of *Serranus* (Serranidae: Serraninae) [56], and *Pseudorhabdosynochus caballeroi* (Oliver, 1984) Kritsky & Beverley-Burton, 1986 from the polyprionid *Stereolepis gigas* Ayres from the Pacific Ocean off Mexico [3, 39]. Many epinephelin groupers had been placed in *Serranus* prior to recent revisions of the Serranidae and recognition of the tribe Epinephelini, and, as a result, Yamaguti [56] may well have collected *P. serrani* from an epinephelin host. Although *Pseudorhabdosynochus amplidiscatum* (Bravo-Hollis, 1954) Kritsky & Beverley-Burton, 1986 was originally described from *Paralabrax maculatofasciatus* (Steindachner) (Serranidae: Serraninae) [2], the species was subsequently reported from two epinephelin hosts [*Epinephelus analogus* Gill and *Epinephelus labriformis* (Jenyns)] from the Pacific coasts of Mexico and Panama [30]; however, these latter records require confirmation. Two other nominal species of *Pseudorhabdosynochus* from non-epinephelin hosts were previously reassigned to other diplectanid genera: *Pseudorhabdosynochus latesi* (Tripathi, 1955) Kritsky & Beverley-Burton, 1986 and *Pseudorhabdosynochus seabassi* Wu, Li, Zhu & Xie, 2005 both from the latid *Lates calcarifer* (Bloch), were transferred to *Laticola* Yang, Kritsky, Sun, Zhang, Shi & Agrawal, 2006 by Yang et al. [59] and Domingues & Boeger [5], respectively.

The majority of described species of *Pseudorhabdosynochus* is known from groupers of the Indo-Pacific and eastern Atlantic regions. The known diversity within the genus in the western Atlantic Ocean and adjacent waters is comparatively small, with only seven species having been described from the region prior to the present study: *Pseudorhabdosynochus americanus* (Price, 1937) Kritsky & Beverley-Burton, 1986 from the Atlantic goliath grouper *Epinephelus itajara* (Lichtenstein) [42]; *Pseudorhabdosynochus beverleyburtonae* (Oliver, 1984) Kritsky & Beverley-Burton, 1986 from dusky grouper *Mycteroperca marginata* (Lowe) [46] (this species was originally described from dusky grouper in the Mediterranean Sea [38]); *Pseudorhabdosynochus monaensis* Dyer, Williams & Bunkley-Williams, 1994 from rock hind *Epinephelus adscensionis* (Osbeck) [6]; *Pseudorhabdosynochus kritskyi* Dyer, Williams & Bunkley-Williams, 1995 from gag *Mycteroperca microlepis* (Goode & Bean) [7]; *Pseudorhabdosynochus yucatanensis* Vidal-Martínez, Aguirre-Macedo & Mendoza-Franco, 1997 from red grouper *Epinephelus morio* (Valenciennes) [50]; *Pseudorhabdosynochus capurroi* Vidal-Martínez & Mendoza-Franco, 1998 from black grouper *Mycteroperca bonaci* (Poey) [52]; and *Pseudorhabdosynochus sulamericanus* Santos, Buchmann & Gibson, 2000 from snowy grouper *Hyporthodus niveatus* (Valenciennes) [46].

The present study was undertaken to further examine the diversity of *Pseudorhabdosynochus* species in the western Atlantic region. To check for infection by members of the genus, the gills of individuals of 23 species of groupers from waters off Florida, Mississippi, Alabama, Puerto Rico, and southern Brazil were surveyed. As a result, 19 species of *Pseudorhabdosynochus* (13 new and six previously described) are reported or described from the region.

## Materials and methods

Groupers were collected by fish trap, hook and line, or longline from marine waters off Florida, Alabama, Mississippi, Puerto Rico, and southern Brazil. In the case of the protected Atlantic goliath grouper, we opportunistically evaluated moribund or freshly dead specimens suspected to have been killed by red tide, a geographically widespread and highly concentrated bloom of the toxin-producing dinoflagellate *Karenia brevis* (Davis) [28]. Groupers were identified by collectors using various resources available at the respective sites. For US collections, most fish specimens were identified morphologically in the field by fisheries biologists familiar with the local fauna; some specimens of questionable identity were returned to the laboratory for further morphological examination and comparison of 16s and 18s rRNA sequences with those published for groupers in GenBank. Morphological identifications of specimens of dusky grouper collected in Brazil were verified by molecular barcoding comparison with sequences available through BOLDsystems (http://www.boldsystems.org). The classification and scientific names of the groupers follow Craig & Hastings [4]; common names of the fishes are those provided in FishBase [11] and verified in Eschmeyer & Fong [8].

When fresh material was available, the host’s gill basket was removed shortly after capture and placed in hot (65–70 °C) 4% phosphate-buffered formalin to relax and fix the parasites. Additional parasite specimens were evaluated from other authors’ collections that had been deposited in the US National Parasite Collection (USNPC). To check for specimens of *Pseudorhabdosynochus* spp. infecting host species not represented in our collections and to supplement those available from the USNPC, we isolated monogenoids that had been incidentally fixed with their hosts and deposited in the Florida Fish and Wildlife Conservation Commission’s Fish and Wildlife Research Institute’s (FWC/FWRI, formerly Florida State Board of Conservation Marine Laboratory) ichthyological specimen collection (FSBC) in St. Petersburg, Florida. To avoid damaging FSBC fish specimens, the gills were not excised but rinsed in situ with a jet of tap water to dislodge the parasites. The rinsate was then evaluated under a dissecting scope to isolate the worms. Fishes from the FSBC collection had been fixed or preserved inconsistently and may have spent some time at ambient temperature in solutions of 50% isopropyl alcohol or 10% buffered formalin; at the time of processing, the fishes and their helminth specimens were stored in 70% ethanol; helminths collected from the rinsate were transferred to 5% phosphate-buffered formalin. As would be expected for specimens fixed with these non-ideal methods, there was substantial intra- and inter-host variance in the physical integrity of monogenoid specimens. Whether collected fresh or isolated from FSBC hosts, materials from each host were placed individually in labeled vials or plastic bags with fixative and shipped to Idaho State University for study. There, a small probe and dissecting microscope were used to isolate diplectanids from the gills or sediment. Some specimens were mounted unstained in Gray and Wess medium for a study of sclerotized structures; other specimens were stained with Gomori’s trichrome [18, 26] and mounted in Canada balsam from beechwood creosote for observation of internal anatomy.

The latter method, during which the stained specimens were mounted in Canada balsam, frequently resulted in the collapse of the proximal two chambers of the male copulatory organ (MCO). In order to minimize collapse of the two chambers, some unstained specimens were carried through a graded ethanol series, cleared in xylene, and then placed in a small watch glass containing a weak solution of Canada balsam in xylene. The xylene was allowed to slowly evaporate over a period of 10–14 days by slightly offsetting the watch glass cover. When the consistency of Canada balsam reached a suitable level, the unstained specimens were individually mounted on a microscope slide under a coverslip.

Illustrations were prepared with the aid of a camera lucida or microprojector. Measurements, all in micrometers, represent straight-line distances between extreme points and are expressed as the mean followed in parentheses by the range and number (*n*) of structures measured; body length included that of the haptor; length of the MCO was represented by a straight-line distance from the distal tip of the cone to the farthest point on the wall of the proximal chamber of the MCO; length of the ventral anchor was obtained from the tip of the superficial root to the distal point on the curve of the anchor shaft and point (use of the tip of the deep root as a point in the measurement was not considered useful because it seldom occurred in the same plane of view as the point and shaft). All measurements were obtained from structures lying within the plane of view under microscopy; structures with portions lying outside the plane of view or those damaged during fixation and mounting were not measured. Terminology of the MCO was in part that suggested by Justine [19]. Terminology of the vagina was adjusted from that of Hinsinger & Justine [17] to include a vaginal vestibule, vaginal sclerite, and vaginal canal; Hinsinger & Justine [17] represented the vagina as only the two former structures. Numbering of haptoral-hook pairs follows the system of Mizelle [32, 33]. Minimum prevalence [23] was provided only when the number of infected and uninfected hosts was known.

Type and voucher specimens collected during the present study were deposited in the US National Museum, Smithsonian Institution, Suitland, Maryland (USNM), the FWC/FWRI’s Invertebrate Specimen Collection, St. Petersburg, Florida (FSBC-I), and the helminth collections of the Natural History Museum, London, UK (NHMUK) and the Muséum National d’Histoire Naturelle, Paris, France (MNHN) as indicated in the respective species accounts. Available specimens of diplectanids from western Atlantic groupers previously accessioned into the USNPC were also examined.

## Results

A total of 23 species of groupers (Serranidae: Epinephelinae: Epinephelini) from the western Atlantic Ocean and adjacent waters was examined for species of *Pseudorhabdosynochus* as follows: Atlantic goliath grouper *Epinephelus itajara* (*n* = 2); red grouper *Epinephelus morio* (*n* = 12); rock hind *Epinephelus adscensionis* (*n* = 2); red hind *Epinephelus guttatus* (Linnaeus) (*n* = 4); Nassau grouper *Epinephelus striatus* (Bloch) (*n* = 1); speckled hind *Epinephelus drummondhayi* Goode & Bean (*n* = 2); snowy grouper *Hyporthodus niveatus* (*n* = 8); Warsaw grouper *Hyporthodus nigritus* (Holbrook) (*n* = 1); yellowedge grouper *Hyporthodus flavolimbatus* (Poey) (*n* = 4); misty grouper *Hyporthodus mystacinus* (Poey) (*n* = 3); gag *Mycteroperca microlepis* (*n* = 13); yellowmouth grouper *Mycteroperca interstitialis* (Poey) (*n* = 2); scamp *Mycteroperca phenax* Jordan & Swain (*n* = 14); dusky grouper *Mycteroperca marginata* (Lowe) (*n* = 4); black grouper *Mycteroperca bonaci* (Poey) (*n* = 1); tiger grouper *Mycteroperca tigris* (Valenciennes) (*n* = 1); yellowfin grouper *Mycteroperca venenosa* (Linnaeus) (*n* = 1); graysby *Cephalopholis cruentata* (Lacepède) (*n* = 3); coney *Cephalopholis fulva* (Linnaeus) (*n* = 5); mutton hamlet *Alphestes afer* (Bloch) (*n* = 1); Atlantic creolefish *Paranthias furcifer* (Valenciennes) (*n* = 1); marbled grouper *Dermatolepis inermis* (Valenciennes) (*n* = 3); and Spanish flag *Gonioplectrus hispanus* (Cuvier) (*n* = 1). All species of groupers were infected with one or more species of *Pseudorhabdosynochus* except the coney, marbled grouper, misty grouper, mutton hamlet, Atlantic creolefish, and Spanish flag. Nineteen (13 new, 6 previously described) species of *Pseudorhabdosynochus* were collected and identified; the *Pseudorhabdosynochus* species found on the yellowfin grouper and one on the yellowmouth grouper were not identified to species because of insufficient material (see Taxonomic Account). The Atlantic creolefish was infected with an undetermined species of Diplectanidae that lacked a reniform quadriloculate MCO and could not be assigned to *Pseudorhabdosynochus*.

Class Monogenoidea Bychowsky, 1937
Subclass Polyonchoinea Bychowsky, 1937
Order Dactylogyridea Bychowsky, 1937
Suborder Dactylogyrinea Bychowsky, 1937
Diplectanidae Monticelli, 1903

### *Pseudorhabdosynochus americanus* (Price, 1937) Kritsky & Beverley-Burton, 1986

Syns *Diplectanum americanum* Price, 1937; *Cycloplectanum americanum* (Price, 1937) Oliver, 1968 (pro parte).

Type host and locality: Atlantic goliath grouper, *Promicrops itaiara* (Lichtenstein) (lapsus) [now *Epinephelus itajara* (Lichtenstein)] (Serranidae: Epinephelinae: Epinephelini): United States (New York Aquarium).

Current records: *Epinephelus itajara*: Conception Key, South Tampa Bay, Florida (27°39.215′ N, 82°40.849′ W), October 20, 2011; off Sea Grape Lane, Vero Beach, Florida (27°38.017′ N, 80°20.896′ W), August 9, 2011 (both new locality records).

Previous records: *Epinephelus itajara*: United States (New York Aquarium) [42].

Unconfirmed and erroneous host and locality records: *Stereolepis gigas* Ayres (Polyprionidae): Salina Cruz, Oaxaca, Mexico (as *Diplectanum americanum*) [3]; *D. americanum* of Caballero & Bravo Hollis [3] renamed *Cycloplectanum caballeroi* Oliver, 1984 by Oliver [38] [now *Pseudorhabdosynochus caballeroi* (Oliver, 1984) Kritsky & Beverley-Burton, 1986]. *Epinephelus gigas* (Brünnich) (Serranidae: Epinephelinae: Epinephelini) [an ambiguous synonym of *Mycteroperca marginata* (Lowe)]: Golfe du Lion, Côte Vermeille, Banyuls-sur-Mer, Mediterranean Sea (as *D. americanum*) [10]; *D. americanum* of Euzet & Oliver [10] considered a synonym of *Cycloplectanum beverleyburtonae* Oliver, 1984 by Oliver [38] [now *Pseudorhabdosynochus beverleyburtonae* (Oliver 1984) Kritsky & Beverley-Burton, 1986]. *Epinephelus guaza* (non-Linnaeus, 1758), a misapplied name [16] (now *M. marginata*) (Serranidae: Epinephelinae: Epinephelini): Bay of Naples, Italy (as *C. americanum*) [49]; *C. americanum* of Ulmer & James [49] considered a synonym of *C. beverleyburtonae* by Oliver [39] (now *P. beverleyburtonae*). *Rivulus harti* (Boulenger) (Rivulidae): Rio Brito, Sucre State, 30 km from Cumaná, en route to Puerto La Cruz, Venezuela (as *C. americanum*) [35] (unconfirmed). *Astyanax bimaculatus* (Linnaeus) (Characidae): Rio Brito, Sucre State, 30 km from Cumaná, en route to Puerto La Cruz, Venezuela (as *C. americanum*) [35] (unconfirmed). *Epinephelus aeneus* (Geoffroy Saint-Hilaire) (Serranidae: Epinephelinae: Epinephelini): southeastern Atlantic Ocean (southwest Africa) (as *C. americanum*) [1]; Oliver [39] stated that *C. americanum* of Aljoshkina [1] may represent *Diplectanum hargisi* Oliver & Paperna, 1984 [now *Pseudorhabdosynochus hargisi* (Oliver & Paperna, 1984) Santos, Buchmann & Gibson, 2000].

Infection site: Gill lamellae.

Minimum prevalence: 100% (2 of 2 *E. itajara* infected).

Specimens studied: 46 voucher specimens, USNM 1251948, 1251949, MNHN HEL437–446; NHMUK 2014.11.14.1–3, FSBC-I 127738–127741.

Museum specimens examined: Holotype, 4 paratypes of *Diplectanum americanum*, USNPC 35703.

#### Redescription (Figs. 1–8)

Body flattened dorsoventrally. Tegumental scales with rounded anterior margins extending from peduncle anteriorly into posterior trunk. Cephalic region broad, with two terminal and two bilateral poorly developed cephalic lobes, three bilateral pairs of head organs, pair of bilateral groups of cephalic-gland cells at level of pharynx. Posterior pair of eyespots lacking lenses, lying immediately anterior to pharynx (two specimens lacking one member of the pair); anterior pair usually absent, often represented by few poorly associated chromatic granules (one specimen with well-developed anterior eyespots lacking lenses); accessory chromatic granules small, irregular, usually anterior to posterior pair of eyespots. Pharynx with muscular wall; esophagus short to nonexistent; intestinal ceca blind, extending posteriorly to near anterior limit of peduncle. Peduncle broad, tapered posteriorly. Haptor with dorsal and ventral anteromedial lobes containing respective squamodiscs and lateral lobes having hook pairs 2–4, 6, 7. Dorsal and ventral squamodiscs subequal, with 19–23 (usually 21) U-shaped rows of rodlets; 1–3 (usually 2) innermost rows closed. Ventral anchor with well-developed superficial root, long deep root having lateral swelling, slightly curved shaft, and short recurved point extending to just past level of tip of superficial root. Dorsal anchor with subtriangular base, poorly developed roots, arcing shaft, recurved point extending past level of superficial tip of base. Ventral bar with slight medial constriction, tapered ends, longitudinal medioventral groove. Paired dorsal bar with spatulate medial end. Hook with elongate depressed thumb, delicate point, uniform shank; filamentous hook (FH) loop nearly shank length. Testis ovate, lying sinistroposterior to germarium; proximal vas deferens, prostatic reservoir not observed; seminal vesicle an indistinct dilation of distal vas deferens, lying just posterior to MCO; ejaculatory bulb not observed. MCO reniform, quadriloculate, with short distal cone, elongate tube with comparatively thick walls, delicate apparently retractile distal filament; walls of two distal chambers thick, walls of proximal two chambers thinner but comparatively rigid. Germarium pyriform, shaped as an inverted comma; germarial bulb lying diagonally at body midlength, with elongate dorsoventral distal loop around right intestinal cecum; ootype lying to left of body midline, with well-developed Mehlis’ gland and giving rise to delicate banana-shaped uterus when empty. Common genital pore ventral, dextral to distal chamber of MCO. Vaginal pore sinistroventral at level of seminal vesicle. Vaginal vestibule delicate; vaginal sclerite with distal funnel and two comparatively large juxtaposed thick-walled chambers; seminal receptacle subspherical, immediately proximal to vagina and anterior to ootype. Bilateral and common vitelline ducts at level of ootype; vitellarium absent in regions of other reproductive organs, otherwise dense throughout trunk.

**Figures 1–8.**
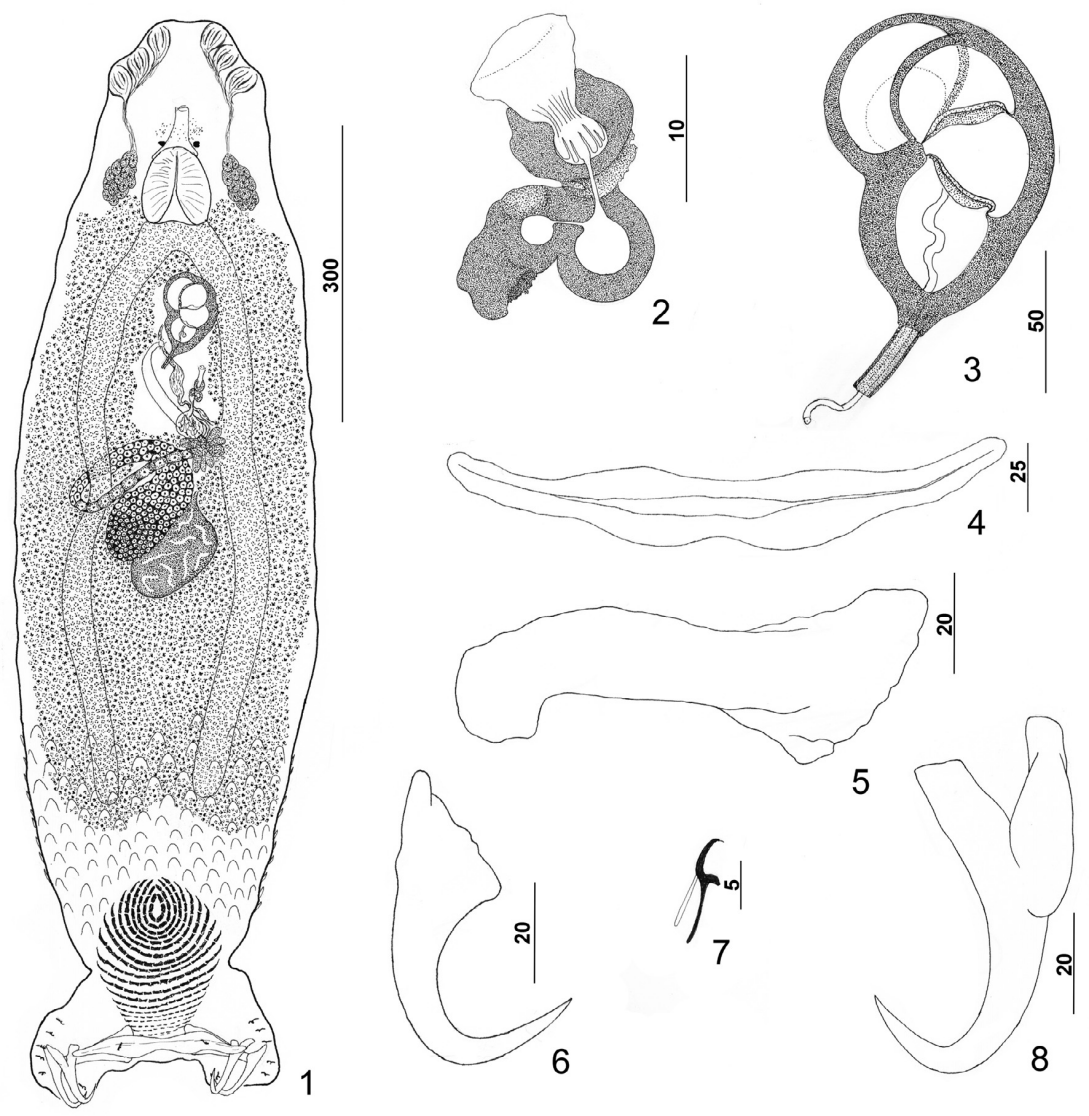
*Pseudorhabdosynochus americanus* (Price, 1937) Kritsky & Beverley-Burton, 1986 from Atlantic goliath grouper *Epinephelus itajara*. 1: whole mount (composite, ventral view; dorsal squamodisc and dorsal anteromedial haptoral lobe not shown); 2: vaginal sclerite (ventral view); 3: male copulatory organ (ventral view); 4: ventral bar; 5: right dorsal bar (ventral view); 6: dorsal anchor; 7: hook; 8: ventral anchor.

Measurements: Body 1007 (863–1223; *n* = 27) long; width at level of germarium 281 (222–347; *n* = 26). Haptor 267 (243–310; *n* = 27) wide; squamodisc 154 (143–170; *n* = 13) long, 140 (109–160; *n* = 12) wide. Ventral anchor 63 (59–67; *n* = 15) long; dorsal anchor 62 (59–66; *n* = 15) long. Ventral bar 157 (139–168; *n* = 13) long; dorsal bar 95 (87–103; *n* = 16) long. Hook 12 (11–13; *n* = 20) long. Pharynx 63 (53–71; *n* = 27) wide. MCO 103 (94–109; *n* = 24) long. Testis 81 (62–115; *n* = 23) long, 78 (58–100; *n* = 23) wide. Germarial bulb 60 (44–74; *n* = 22) wide.

#### Remarks

This species, originally described as *Diplectanum americanum* in 1937 [42], was the first diplectanid known to have a reniform compartmentalized MCO. Over the next 30 years, only three species with this feature were described: *Diplectanum epinepheli* Yamaguti, 1938 [now *Pseudorhabdosynochus epinepheli* (Yamaguti, 1938) Kritsky & Beverley-Burton, 1986]; *Diplectanum amplidiscatum* Bravo-Hollis, 1954 [now *Pseudorhabdosynochus amplidiscatus* (Bravo-Hollis, 1954) Kritsky & Beverley-Burton, 1986]; and *Diplectanum serrani* Yamaguti, 1953 [now *Pseudorhabdosynochus serrani* (Yamaguti, 1953) Kritsky & Beverley-Burton, 1986] [2, 55, 56]. Lacking information on host specificity and intraspecific limits on morphology among the diplectanid species, subsequent investigators frequently assigned specimens possessing a compartmentalized MCO and parasitizing a variety of fish hosts to *D. americanum*. Several of these assignments [1, 3, 10, 49] were determined to represent other diplectanid species [25, 38, 39]. Other reports of *D. americanum* from hosts other than the Atlantic goliath grouper are probably erroneous. As a result, the present specimens likely represent the only valid record of *P. americanus* since its original description and the only one of the species from Atlantic goliath grouper in its natural environment.

Oliver [38] considered *Diplectanum epinepheli, D. serrani, D. amplidiscatum, D. latesi* Tripathi, 1957, *D. melanesiensis* Laird, 1958, and *Pseudorhabdosynochus epinepheli* to be junior synonyms of *D. americanum* when he transferred the species to his newly proposed *Cycloplectanum* Oliver, 1968 as its type species. These synonymies were later reversed when it was determined that *Cycloplectanum* was a junior subjective synonym of *Pseudorhabdosynochus* [25]. Kritsky & Beverley-Burton [25] placed all of the above-listed species in *Pseudorhabdosynochus* as valid species, except that *P. epinepheli* Yamaguti, 1958 (type species of *Pseudorhabdosynochus* by monotypy) was considered a junior subjective synonym of *P. epinepheli* (Yamaguti, 1938) Kritsky & Beverley-Burton, 1986. Yang et al. [59] subsequently transferred *P. latesi* (Tripathi, 1957) Kritsky & Beverley-Burton, 1986 to *Laticola* Yang, Kritsky, Sun, Zhang, Shi & Agrawal, 2006 (Diplectanidae).

After examination of the type specimens of *Diplectanum americanum* and *D. hargisi*, Yang et al. [58] considered the latter species a junior (subjective) synonym of *D. americanum*. They based the synonymy on similarity of measurements provided by Aljoshkina [1] and by Oliver [39] and Santos et al. [46], who first suggested the synonymy. In addition, Yang et al. [58] supported their proposed synonymy by stating that “the vaginal hard parts and other sclerotized structures of the types of both [species]” and their measurements were “virtually identical”. Although the vaginal sclerites of the two species appear to have some common features, the drawings of these structures by Yang et al. [58] are relatively diagrammatic and hardly diagnostic, and our examination of the types of *D. americanum* showed that many features of the vaginal sclerite were not clearly visible in these specimens. That the two species are doubtful synonyms is supported by their respective geographic and host distributions, with *P. hargisi* known only from the white grouper *Epinephelus aeneus* (Geoffroy Saint-Hilaire) in the eastern Mediterranean Sea and *P. americanus* unequivocally from the Atlantic goliath grouper in the western Atlantic region. As a result, the synonymy of the two species is herein rejected, while recognizing that *P. hargisi* requires redescription that should be based on new collections from its type host from or near the type locality.

Examination of the holotype and four paratypes (USNPC 35703) confirmed that present specimens from the Atlantic goliath grouper were conspecific with *D. americanum*. Although the type specimens are in poor condition, their visible haptoral sclerites and MCO (the latter often damaged) were basically identical to those of current specimens. The type specimens differed from specimens collected during the present study in that they possessed three or four eyespots (one anterior eyespot dissociated or absent in two specimens); most specimens of the current collection possessed only the posterior pair of eyespots.

*Pseudorhabdosynochus americanus* is easily distinguished from its congeners that infect groupers in the western Atlantic region by its unique vaginal sclerite consisting of a distal funnel and two comparatively large juxtaposed thick-walled chambers. The Atlantic goliath grouper, the largest grouper species occurring in the region (up to 2.5 m total length) [11], is likely the only natural host for *P. americanus*.

### *Pseudorhabdosynochus yucatanensis* Vidal-Martínez, Aguirre-Macedo & Mendoza-Franco, 1997

Type host and locality: Red grouper, *Epinephelus morio* (Valenciennes) (Serranidae: Epinephelinae: Epinephelini): Progreso, Yucatan, Mexico.

Current records: *Epinephelus morio*: Florida Middle Grounds, Gulf of Mexico (28.208–28.511° N, 84.054–84.119° W), May 2–3, 2009, October 1–8, 2009; an artificial reef in the Gulf of Mexico off Mississippi (30.042° N, 88.586° E), January 30, 2003 (all new locality records).

Previous records: *Epinephelus morio*: Various localities off the Yucatan Peninsula, Mexico [Celestun, Progreso, Sisal, Chelem, Telchac, Chuburna, Chicxulub, and Rio Lagartos (all Yucatan State), Campeche (Campeche State), and Chiquila (Quintana Roo State)] [34, 50, 51, 53, 54].

Infection site: Gill lamellae.

Minimum prevalence: 58% (7 of 12 red grouper from Florida infected).

Specimens studied: 47 voucher specimens from Florida, USNM 1276171–1276175, NHMUK 2014.11.14.4–5, MNHN HEL447–453, FSBC-I 127735–127737; 9 voucher specimens from Mississippi, USNM 1276176.

Museum specimens examined: Holotype, CINVESTAV-IPN (No. 96-5); 3 paratypes, USNPC 87301 [USNPC records indicate that Dr. David Gibson (2005, unpublished) suggested that the three paratypes probably represented *P. sulamericanus* Santos, Buchmann & Gibson, 2000].

#### Redescription (Figs. 9–16)

Body elongate ovate, flattened dorsoventrally, with slight constriction at level of MCO. Numerous tegumental scales with rounded anterior margins extending from posterior ends of intestinal ceca into peduncle. Cephalic region broad, with rounded terminal and two poorly developed bilateral lobes; three bilateral pairs of head organs; pair of bilateral groups of cephalic-gland cells at level of pharynx. Four eyespots lacking lenses immediately anterior to pharynx; members of posterior pair larger, equidistant or slightly closer together than those of anterior pair; accessory chromatic granules small, irregular in outline, uncommon or absent in cephalic region. Pharynx ovate, muscular; esophagus short to nonexistent; intestinal ceca blind, extending posteriorly to level of peduncle. Peduncle broad, tapering posteriorly. Haptor subtrapezoidal, with dorsal and ventral anteromedial lobes containing respective squamodiscs and lateral lobes having hook pairs 2–4, 6, 7. Squamodiscs similar, each with 11 or 12 (usually 12) U-shaped rows of rodlets; innermost row closed. Ventral anchor with elongate superficial root, shorter deep root having lateral swelling, curved shaft, and moderately long recurved point extending to level of tip of superficial root. Dorsal anchor with subtriangular base, superficial root short to lacking, knoblike deep root, curved shaft, recurved point extending past level of tip of superficial root. Ventral bar with slight medial constriction, tapered ends, longitudinal medioventral groove. Paired dorsal bar with slightly spatulate medial end. Hook with elongate slightly depressed thumb, delicate point, uniform shank; FH loop nearly shank length. Testis subspherical, lying immediately posterior to germarium; proximal vas deferens not observed; seminal vesicle a simple dilation of distal vas deferens, lying just posterior to MCO; ejaculatory bulb apparently absent; large vesicle (prostatic reservoir?) with translucent contents lying dorsal to common genital pore. MCO reniform, quadriloculate, with moderately long cylindrical distal cone; distal tube with delicate wall; terminal filament delicate, variable in length; walls of three distal chambers comparatively thick; proximal chamber with delicate wall, frequently collapsing during mounting of specimen on slide. Germarium pyriform; germarial bulb lying slightly to right of body midline, with elongate dorsoventral distal loop around right intestinal cecum; ootype lying slightly to left of body midline, with well-developed Mehlis’ gland and giving rise to delicate banana-shaped uterus when empty. Common genital pore ventral, dextral to distal chamber of MCO. Vaginal pore sinistroventral at or slightly anterior to level of seminal vesicle; vagina with distal vestibule, small vaginal sclerite having two small tandem chambers; vaginal canal unsclerotized, extending diagonally within body to seminal receptacle. Seminal receptacle lying on body midline immediately anterior to ootype. Bilateral and common vitelline ducts not observed; vitellarium absent in regions of other reproductive organs, otherwise extending from level of MCO to anterior limit of peduncle.

**Figures 9–16.**
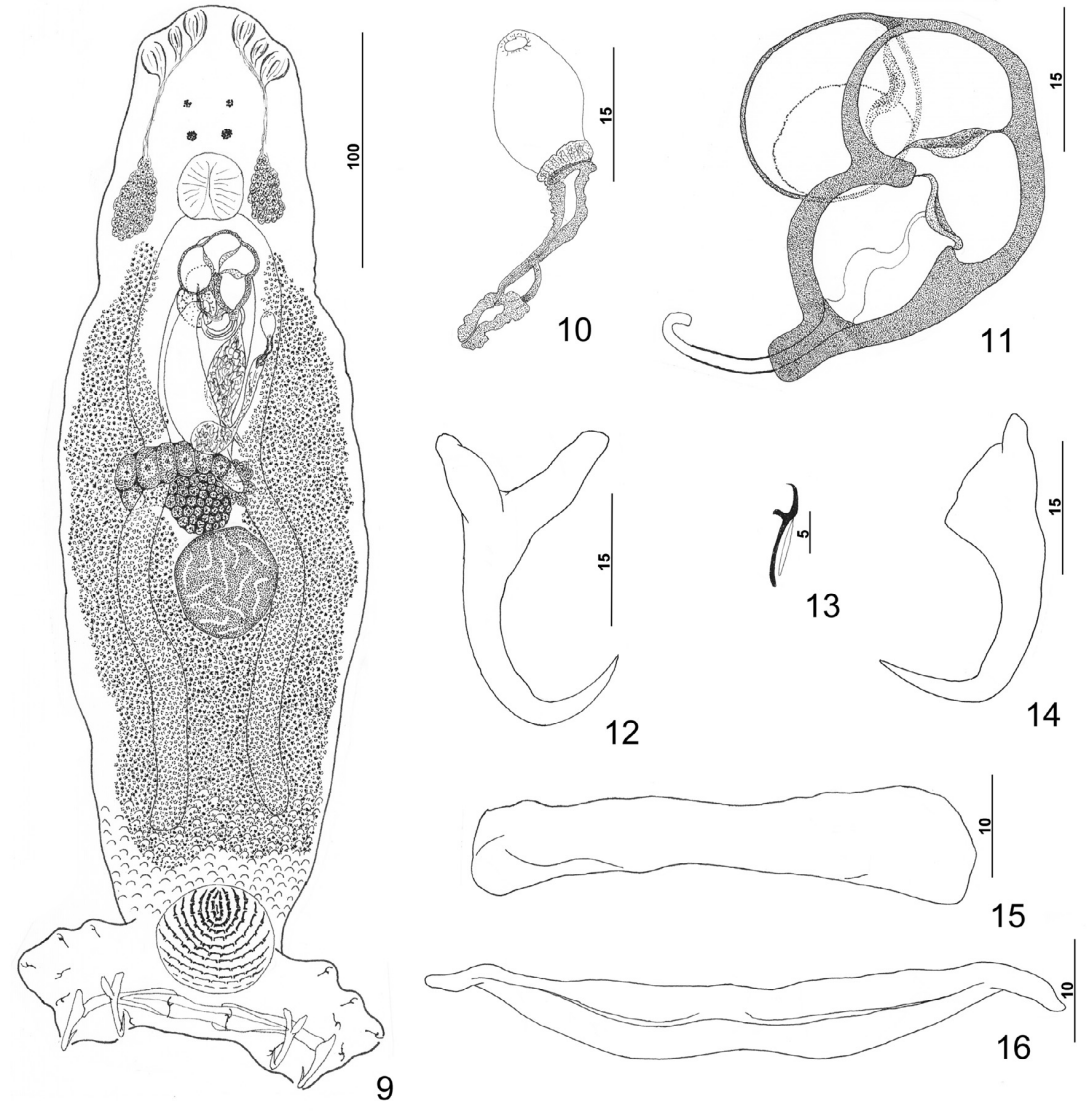
*Pseudorhabdosynochus yucatanensis* Vidal-Martínez, Aguirre-Macedo & Mendoza-Franco, 1997 from red grouper *Epinephelus morio*. 9: whole mount (composite, ventral view; dorsal squamodisc and dorsal anteromedial haptoral lobe not shown); 10: vaginal vestibule and sclerite (ventral view); 11: male copulatory organ (ventral view); 12: ventral anchor; 13: hook; 14: dorsal anchor; 15: right dorsal bar (ventral view); 16: ventral bar.

Measurements: Body 406 (299–489; *n* = 22) long; width at level of germarium 145 (117–172; *n* = 23). Haptor 145 (125–160; *n* = 24) wide; squamodisc 47 (42–52; *n* = 22) long, 54 (47–62; *n* = 23) wide. Ventral anchor 34 (32–36; *n* = 15) long; dorsal anchor 33 (31–34; *n* = 16) long. Ventral bar 69 (64–76; *n* = 15) long; dorsal bar 50 (47–54; *n* = 15) long. Hook 11–12 (*n* = 22) long. Pharynx 30 (27–35; *n* = 26) wide. MCO 43 (39–47; *n* = 21) long. Testis 42 (34–52; *n* = 21) long, 41 (30–50; *n* = 21) wide. Germarial bulb 30 (25–35; *n* = 20) wide.

#### Remarks

Examination of the holotype, three paratypes, and voucher specimens from red grouper off Florida and Mississippi indicated that the original description of *P. yucatanensis* [50] was based on specimens representing two distinct species of *Pseudorhabdosynochus*. Figures 1A and 1C in the original description show the distal parts of the vagina to comprise a weakly sclerotized vaginal vestibule and a large vaginal sclerite having an elongate sigmoid tube originating from a comparatively large thick-walled chamber, while the vaginal sclerites of the holotype and three paratypes were noticeably smaller, each possessing a short distal tube and two small tandem chambers (Fig. 10). The collections from Florida and Mississippi included many specimens representing the two forms.

The comparative morphology of the vaginal sclerite is one of the primary features defining species of *Pseudorhabdosynochus*. Figures 1A and 1C in the original description of *P. yucatanensis* would suggest that the species is defined by the larger vaginal sclerite having a single thick-walled chamber. However, a species is not unequivocally defined by the original description but rather by the holotype, which in this case possessed the smaller sclerite. As a result, *P. justinella* n. sp. is proposed and described below for the form having the larger sclerite as depicted in Figure 1C by Vidal-Martínez et al. [50], and *P. yucatanensis* (s. s.) is redescribed and assigned to specimens with the smaller sclerite (Fig. 10).

Vidal-Martínez et al. [50] stated that tegumental scales were absent, and their Figure 1I suggests that comparatively few rodlets occur in the respective rows of the squamodiscs of *P. yucatanensis*. Tegumental scales, however, are clearly visible, and the concentric rows in the squamodiscs have as many as five or six more rodlets per row in specimens of both *P. yucatanensis* (s. s.) and *P. justinella* n. sp. from Florida. In addition, a few tegumental scales were observed along the margins of the peduncle in one of the paratypes of *P. yucatanensis* deposited in the USNPC. These differences may be a result of fixation procedures used for the type specimens, as tegumental scales and the rodlets of the squamodiscs are frequently lost if fixation does not occur immediately after the death of the helminth.

**Figures 17–24.**
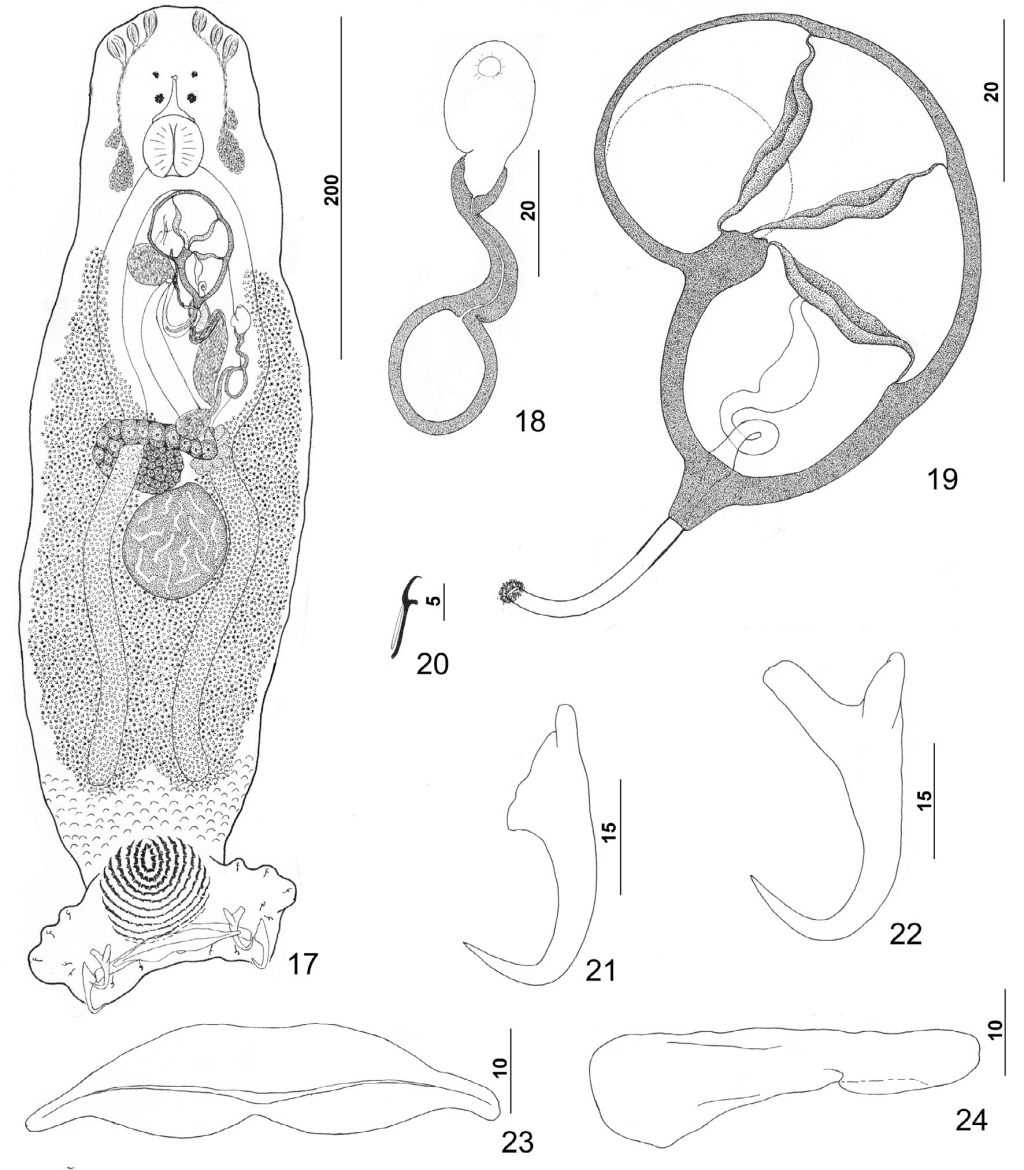
*Pseudorhabdosynochus justinella* n. sp. from red grouper *Epinephelus morio*. 17: whole mount (composite, ventral view; dorsal squamodisc and dorsal anteromedial haptoral lobe not shown); 18: vaginal vestibule and sclerite (ventral view); 19: male copulatory organ (ventral view); 20: hook; 21: dorsal anchor; 22: ventral anchor; 23: ventral bar; 24: left dorsal bar (ventral view).

Several other differences between present specimens and the original account of *P. yucatanensis* are in part a result of the original description being based on two distinct species. Figures 1A (whole mount), 1B (MCO), and 1H (ventral bar) of Vidal-Martínez et al. [50] are undoubtedly from specimens of *P. justinella* (compare with Figs. 17, 19, 23). The original figures show the whole mount to have a vaginal sclerite with a single large chamber, the comparatively large MCO with delicate chamber walls and a tapered cone, and the ventral bar being short and robust, all features of *P. justinella*. In *P. yucatanensis* (s. s.), the vaginal sclerite is comparatively small with two chambers (Fig. 10), the smaller MCO has a cylindrical cone and robust chamber walls (Fig. 11), and the ventral bar is slender and elongate (Fig. 16).

Finally, the original description of *P. yucatanensis* includes several erroneous statements and depictions. The whole-mount figure shows the germarium looping the right intestinal cecum ventrodorsally (germarium loops the right cecum dorsoventrally in all species of *Pseudorhabdosynochus*); the description and whole-mount drawing (Fig. 1A) suggest that only four pairs of hooks are present (the species possesses a full complement of seven pairs of hooks having the usual distribution in the haptor [32, 33]); and although eggs were not observed in the specimens of *P. yucatanensis* (s. s.) from Florida, the original measurements of the egg (22 long × 12 wide) are hardly large enough as all eggs observed in the species of *Pseudorhabdosynochus* collected during the present study are in the order of 100 μm in length.

*Pseudorhabdosynochus yucatanensis* (s. s.) most closely resembles *P. meganmarieae* n. sp. in the comparative morphology of their vaginal sclerites. In both species, the sclerite possesses two small chambers and a distal funnel-shaped tube, but in *P. yucatanensis*, the sclerite is smaller and more delicate than in *P. meganmarieae*. The MCO of *P. yucatanensis* has a cylindrical cone and thick walls of the distal three chambers, while the cone is tapered and the walls of distal chambers are comparatively thin in *P. meganmarieae*. Finally, the shafts of the dorsal and ventral anchors of *P. yucatanensis* are comparatively short and arcing, but in *P. meganmarieae*, they are noticeably longer and minimally arced.

### *Pseudorhabdosynochus justinella* n. sp.

urn:lsid:zoobank.org:act:7ADEDBC4-1E26-484E-B4A5-CB19C9F57AF4

Syn. *Pseudorhabdosynochus yucatanensis* Vidal-Martínez, Aguirre-Macedo & Mendoza-Franco, 1997 (pro parte).

Type host and locality: Red grouper, *Epinephelus morio* (Valenciennes) (Serranidae: Epinephelinae: Epinephelini): Florida Middle Grounds, Gulf of Mexico (28.208–28.511° N, 84.054–84.187° W), May 2–12, 2009, October 1–8, 2009.

Other records: *Epinephelus morio*: Gulf of Mexico (an area off Mississippi with numerous artificial reefs) (30.042° N, 88.586° E), January 30, 2003.

Previous records: *Epinephelus morio*: Progreso, Yucatan State, Mexico (as *P. yucatanensis*) [50].

Unconfirmed records (all as *P. yucatanensis*): *Epinephelus morio*: Various localities off the Yucatan Peninsula, Mexico [Celestun, Progreso, Sisal, Chelem, Telchac, Chuburna, Chicxulub, and Rio Lagartos (all Yucatan State), Campeche (Campeche State), and Chiquila (Quintana Roo State)] [34, 51, 53, 54].

Infection site: Gill lamellae.

Minimum prevalence: 92% (11 of 12 *E. morio* from Florida infected).

Specimens studied: Holotype, USNM 1251935; 49 paratypes, USNM 1251936–1251946, NHMUK 2014.11.14.35–40, MNHN HEL504–510, FSBC-I 127744–127748; 8 voucher specimens from Mississippi, USNM 1251947.

Museum specimens examined: Holotype of *P. yucatanensis*, CINVESTAV-IPN (No. 96-5); 3 paratypes of *P. yucatanensis*, USNPC 87301.

Etymology: The specific name is in honor of our friend and colleague Dr. Jean-Lou Justine, Muséum National d’Histoire Naturelle, Paris, France, in recognition of his extensive work on the species of *Pseudorhabdosynochus* occurring in the western Pacific Ocean and for his support of the present study by providing type specimens from the MNHN.

#### Description (Figs. 17–24)

Body fusiform, dorsoventrally flattened, with a slight constriction at level of MCO; peduncle with small tegumental scales having rounded anterior margins. Cephalic region broad, with two terminal and two bilateral poorly developed lobes, three bilateral pairs of head organs, two bilateral groups of cephalic-gland cells at level of pharynx. Four eyespots lacking lenses immediately anterior to pharynx; members of posterior pair larger, slightly closer together than those of anterior pair; accessory chromatic granules small, irregular in outline, uncommon in cephalic region. Pharynx ovate to subspherical, muscular; esophagus short to nonexistent; intestinal ceca blind, extending posteriorly to level of peduncle, with ends slightly diverging. Peduncle broad, tapered posteriorly. Haptor subtrapezoidal, with dorsal and ventral anteromedial lobes containing respective squamodiscs and lateral lobes having hook pairs 2–4, 6, 7. Dorsal and ventral squamodiscs similar, subequal, each with 10–12 (usually 11) U-shaped rows of rodlets; innermost row usually closed. Ventral anchor with elongate superficial root, slightly shorter deep root having lateral swelling, slightly curved shaft, and moderately long recurved point extending just past level of tip of superficial root. Dorsal anchor with subtriangular base, poorly defined superficial root, elongate deep root, arcing shaft, recurved point extending past level of tip of superficial root. Ventral bar robust, with medial constriction, tapered ends, longitudinal medioventral groove. Paired dorsal bar with spatulate medial end. Hook with elongate slightly depressed thumb, delicate point, uniform shank; FH loop nearly shank length. Testis subspherical, lying sinistroposterior to germarium; proximal vas deferens not observed. Seminal vesicle a fusiform dilation of distal vas deferens, lying just posterior to MCO; ejaculatory bulb pyriform; large vesicle (prostatic reservoir?) lying to right of MCO. MCO reniform, quadriloculate, with short tapered cone, distal tube with delicate wall continuous with distal filament; distal filament with bulbous end; walls of chambers comparatively delicate, often collapsed. Germarium pyriform, lying at midlength of trunk, with dorsoventral distal loop around right intestinal cecum; ootype lying slightly to left of body midline and surrounded by well-developed Mehlis’ gland; uterus banana shaped when empty, delicate. Common genital pore ventral, dextral to distal chamber of MCO. Vaginal pore sinistroventral at level of seminal vesicle; vaginal vestibule with delicate wall; vaginal sclerite with small distal funnel, sigmoid tube leading to thick-walled ovate to subspherical chamber; proximal vaginal canal arising from chamber, extending to subovate seminal receptacle near body midline immediately anterior to ootype and Mehlis’ gland. Bilateral and common vitelline ducts not observed; vitellarium dense, absent in regions of other reproductive organs, otherwise extending in bilateral fields of trunk from level of MCO to anterior limit of peduncle; bilateral fields confluent posterior to testis. Egg ovate (often collapsed due to staining and mounting procedures), lacking filaments.

Measurements: Body 511 (420–661; *n* = 28) long, width at level of germarium 157 (114–202; *n* = 30). Haptor 152 (131–176; *n* = 27) wide; squamodisc 55 (43–61; *n* = 41) long, 68 (57–76; *n* = 42) wide. Ventral anchor 39 (36–42; *n* = 15) long; dorsal anchor 36 (34–38; *n* = 17) long. Ventral bar 67 (63–77; *n* = 14) long; dorsal bar 55 (51–59; *n* = 16) long. Hook 11–12 (*n* = 21) long. Pharynx 34 (29–43; *n* = 29) wide. MCO 62 (56–68; *n* = 28) long. Testis 64 (51–82; *n* = 22) long, 65 (50–77; *n* = 22) wide. Germarial bulb 34 (26–43; *n* = 19) wide. Egg 118–119 (*n* = 1) long, 54–55 (*n* = 1) wide.

#### Remarks

*Pseudorhabdosynochus justinella* n. sp. is most similar to *P. woodi* n. sp. based on the comparative morphology of the vaginal sclerite, the ventral bar, and the ventral and dorsal anchors. In both species, the vaginal sclerite possesses an elongate sigmoid distal tube attached to the distal end of the chamber, the ventral bar is short and robust, the deep root of the ventral anchor is shorter than the superficial root, and the dorsal anchors of the two species are morphologically indistinguishable. *Pseudohaliotrema justinella* differs from *P. woodi* by having a vaginal sclerite with a larger (~20 μm in diameter) subspherical chamber (vaginal sclerite with a small [~10 μm in length] ovate chamber in *P. woodi*).

*Pseudorhabdosynochus justinella*, *P. woodi*, and *P. bunkleywilliamsae* spp. n., from three western Atlantic species of groupers making up the terminal clade (*E. guttatus* (*E. morio*, *E. striatus*)) within *Epinephelus* [4], appear to form a complex of morphologically similar species characterized by having a vaginal sclerite with a distal sigmoid tube arising from the distal end of the chamber. Although phylogenetic analyses of the helminths are wanting, occurrence of these parasites on closely related congeneric groupers suggests that some level of coevolution occurred between the parasites and their respective hosts. Similar relationships between species of *Pseudorhabdosynochus* species and their grouper hosts assigned to *Mycteroperca* may have also been recognized (see Remarks for *P. kritskyi*), indicating that the *Pseudorhabdosynochus* species and their hosts may provide useful models for investigating coevolutionary relationships.

The original description of *P. yucatanensis* [50] was based on a series of 14 specimens that included members of both *P. yucatanensis* (s. s.) and *P. justinella* (see Remarks for *P. yucatanensis*). *Pseudorhabdosynochus yucatanensis* has been subsequently reported only by Vidal-Martínez and coworkers from regions near its type locality (see References listed above in “Previous records”). Because Vidal-Martínez and coworkers apparently did not differentiate between the two species, the latter records probably also included specimens of *P. justinella*. Nonetheless, those records published subsequent to the original description of *P. yucatanensis* require confirmation for the presence of both *P. yucatanensis* and *P. justinella*.

### *Pseudorhabdosynochus kritskyi* Dyer, Williams & Bunkley-Williams, 1995

Type host and locality: Gag, *Mycteroperca microlepis* (Goode & Bean) (Serranidae: Epinephelinae: Epinephelini): Gulf of Mexico (29°15′ N, 86°36′ W).

Current records: *Mycteroperca microlepis*: Florida Middle Grounds, Gulf of Mexico (28.271–28.604° N, 84.090–84.362° W), May 8–11, 2009, October 6, 2009; Gulf of Mexico, ~60 km s of Mobile Bay, Alabama, November 6, 2001; Tampa Bay, Florida (27.619–27.740° N, 82.654–82.905° W), September 3, 2008, August 16, 2010, March 29, 2011 (new locality records).

Previous record: *Mycteroperca microlepis* (Serranidae: Epinephelinae: Epinephelini): Gulf of Mexico (29°15′ N, 86^°^36′ W) [7].

Unconfirmed records: *Mycteroperca bonaci*: Desecheo Island, Puerto Rico [44]. *Mycteroperca tigris* (Valenciennes): Desecheo Island, Puerto Rico [44]. *Mycteroperca venenosa* (Linnaeus): Rincón and Desecheo Island, Puerto Rico [44]. *Epinephelus guttatus*: Lajas, Mayagüez, and Desecheo Island, Puerto Rico [44]. *Cephalopholis fulva* (Linnaeus): Lajas, Puerto Rico [44].

Infection site: Gill lamellae.

Minimum prevalence: 85% (11 of 13 *M. microlepis* from Florida infected).

Specimens studied: 62 voucher specimens from *M. microlepis* off Florida, USNM 1276187–1276194, NHMUK 2014.11.14.7–8, MNHN HEL454–457, FSBC-I 127728, 127729; 8 voucher specimens from *M. microlepis* off Alabama, USNM 1276195.

Museum specimens examined: Holotype, 8 paratypes (*P. kritskyi*), USNPC 83991, 83992.

#### Redescription (Figs. 25–32)

Body dorsoventrally flattened. Tegument smooth, scales absent. Cephalic region broad, with two terminal and two bilateral poorly developed lobes, three bilateral pairs of head organs, pair of bilateral groups of cephalic-gland cells at level of pharynx. Four eyespots immediately anterior to pharynx, lacking lenses; members of posterior pair slightly larger, closer together than those of anterior pair; accessory chromatic granules small, irregular in outline, usually absent in cephalic region. Pharynx ovate, muscular; esophagus short to nonexistent; intestinal ceca blind, extending posteriorly to peduncle, diverging posterior to testis. Peduncle broad. Haptor subtriangular, with dorsal and ventral anteromedial lobes containing respective squamodiscs and lateral lobes having hook pairs 2–4, 6, 7. Squamodiscs subequal, with 14 or 15 U-shaped rows of rodlets; three or four innermost rows oval, closed. Ventral anchor with elongate superficial root, long deep root having lateral swelling, slightly curved shaft, and short recurved point extending just short of level of tip of superficial root. Dorsal anchor with subtriangular base, superficial root short to lacking, moderately long deep root, slightly arcing shaft, recurved point extending past level of tip of superficial root. Ventral bar with medial constriction, tapered ends, longitudinal medioventral groove. Paired dorsal bar with enlarged medial end. Hook with elongate slightly depressed thumb, delicate point, uniform shank; FH loop nearly shank length. Testis subspherical, usually with indentation of posterior margin suggesting two posterior lobes; proximal vas deferens dorsoventrally looping left intestinal cecum; seminal vesicle a simple dilation of distal portion of vas deferens, lying just posterior to MCO; vas deferens entering large subspherical ejaculatory bulb; ejaculatory duct entering portal to MCO; large vesicle (prostatic reservoir?) lying to right of MCO. MCO reniform, quadriloculate, with short tapered cone, elongate distal tube, and variable apparently retractile filament (usually not observed); walls of two distal chambers thick, walls of chambers becoming thinner proximally. Germarium pyriform; germarial bulb dextral, lying diagonally at body midlength, with elongate dorsoventral loop around right intestinal cecum; ootype lying to left of body midline; Mehlis’ gland not observed; uterus delicate, banana shaped when empty. Common genital pore ventral, dextral to MCO. Vaginal pore sinistroventral at level of seminal vesicle; vagina with distal vestibule; vaginal sclerite having sclerotized tube with distal recurved and funnel-shaped terminus opening into vestibule; single chamber usually spherical, with thick wall; proximal vaginal canal delicate, leading to seminal receptacle. Seminal receptacle near body midline. Bilateral vitelline ducts at level of origin of uterus; vitellarium absent in regions of other reproductive organs, otherwise dense throughout trunk.

**Figures 25–32.**
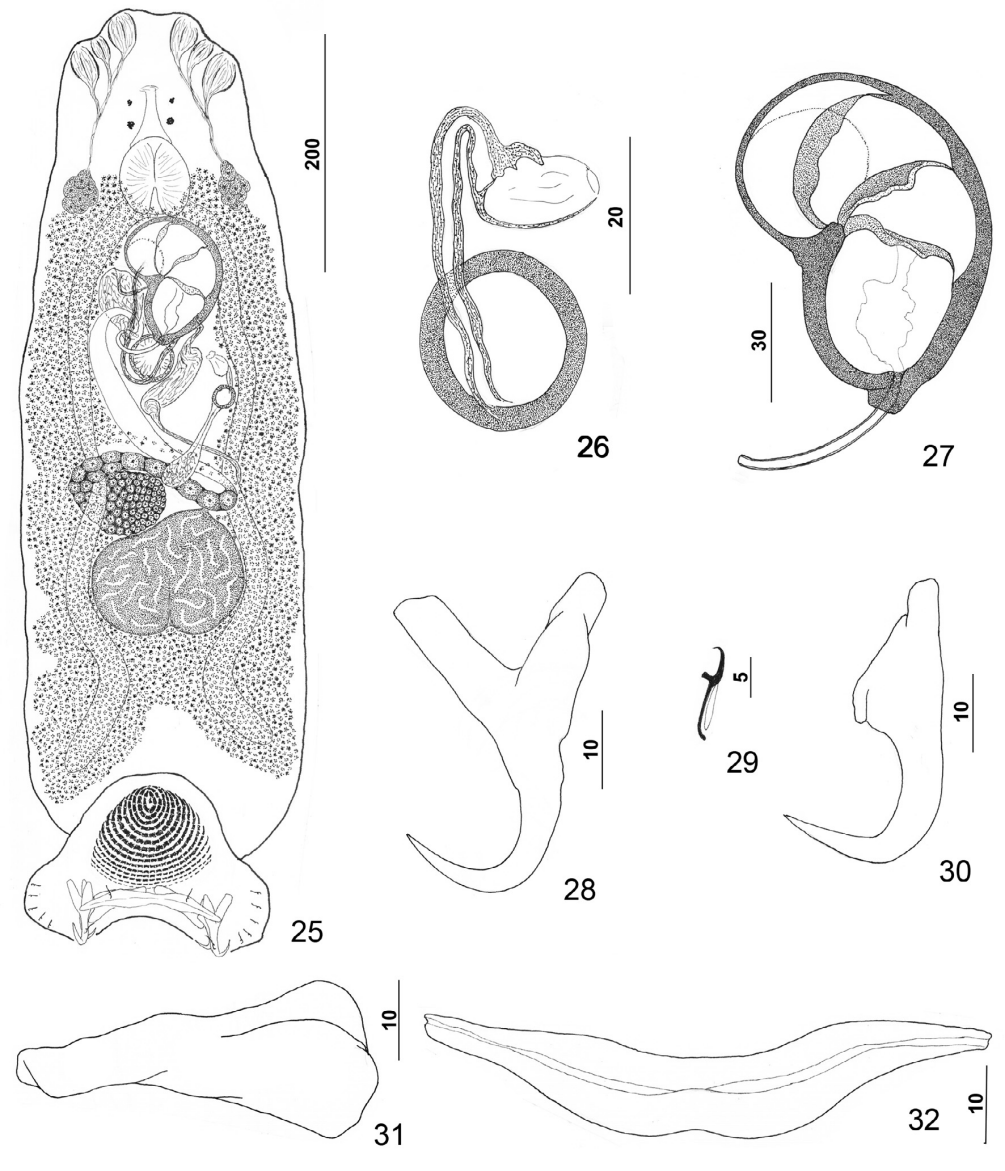
*Pseudorhabdosynochus kritskyi* Dyer, Williams & Bunkley-Williams, 1995 from gag *Mycteroperca microlepis*. 25: whole mount (composite, ventral view; dorsal squamodisc and dorsal anteromedial haptoral lobe not shown); 26: vaginal vestibule and sclerite (dorsal view); 27: male copulatory organ (ventral view); 28: ventral anchor; 29: hook; 30: dorsal anchor; 31: right dorsal bar (ventral view); 32: ventral bar.

Measurements: Body 733 (606–817; *n* = 17) long; width at level of germarium 221 (161–268; *n* = 20). Haptor 187 (142–209; *n* = 19) wide; squamodisc 62 (44–79; *n* = 15) long, 81 (63–93; *n* = 17) wide. Ventral anchor 40 (33–44; *n* = 19) long; dorsal anchor 38 (33–43; *n* = 19) long. Ventral bar 78 (64–102; *n* = 14) long; dorsal bar 50 (43–61; *n* = 17) long. Hook 12 (11–13; *n* = 22) long. Pharynx 54 (47–64; *n* = 17) wide. MCO 114 (101–126; *n* = 19) long. Testis 91 (73–111; *n* = 15) long, 137 (119–159; *n* = 15) wide. Germarial bulb 60 (47–70; *n* = 19) wide.

#### Remarks

Groupers assigned to *Mycteroperca* are parasitized by a complex of similar species of *Pseudorhabdosynochus* that differ from congeners infecting other western Atlantic serranids primarily in the comparative morphology of the vaginal sclerite. The complex includes *P. kritskyi* from *M. microlepis*, *Pseudorhabdosynochus hyphessometochus* n. sp. from *M. interstitialis*, *P. capurroi* from *M. bonaci*, *Pseudorhabdosynochus vascellum* n. sp., and *Pseudorhabdosynochus contubernalis* n. sp. from *M. phenax* and *Pseudorhabdosynochus mycteropercae* n. sp. from *M. tigris*. In these species, the vaginal sclerite has a single subspherical to ovate chamber and a distal tube that is strongly recurved near its articulation with the vaginal vestibule (Fig. 26).

*Pseudorhabdosynochus kritskyi* differs from *P. vascellum* and *P. hyphessometochus* by having a large cavity within the chamber of the vaginal sclerite (cavity comparatively small in *P. vascellum* and *P. hyphessometochus*) and from *P. vascellum* and *P. contubernalis* by having a short heavy cone of the MCO (cone delicate in latter two species). It is distinguished from *P. mycteropercae* by having comparatively short dorsal and ventral anchor shafts and a dorsal bar with an enlarged medial end (medial end of dorsal bar spatulate in *P. mycteropercae*). It differs further from these species by having more rows of rodlets in the haptoral squamodiscs (14 or 15 rows in *P. kritskyi*, 11 or 12 in *P. vascellum*, 12–14 in *P. contubernalis*, and 12 or 13 in *P. mycteropercae* and *P. hyphessometochus*). Finally, the tegument is smooth and lacking scales in *P. kritskyi*, *P. capurroi*, *P. vascellum*, *P. hyphessometochus*, and *P. mycteropercae* (tegument scaled in posterior trunk and peduncle in *P. contubernalis*).

In view of the similarity of species infecting groupers assigned to *Mycteroperca*, the records of *P. kritskyi* from *M. bonaci*, *M. tigris*, *M. venenosa*, *E. guttatus*, and *C. fulva* in Puerto Rico [44] require confirmation. Unfortunately, Rios [44] apparently did not deposit voucher specimens of the helminths from Puerto Rican groupers, and his material was not available for study.

### *Pseudorhabdosynochus capurroi* Vidal-Martínez & Mendoza-Franco, 1998

Type host and locality: Black grouper, *Mycteroperca bonaci* (Poey) (Serranidae: Epinephelinae: Epinephelini): Chuburna, Yucatan State, Mexico.

Current record: *Mycteroperca bonaci* (FSBC 988): ~1 km northwest of Loggerhead Key, Dry Tortugas, Florida (24.641° N, 82.931° W), April 26, 1967.

Previous records: *Mycteroperca bonaci*: Chuburna (21°16′ N, 87°47′ W), Celestun (20°45′ N, 90°15′ W) and Progreso (21°17′ N, 89°40′ W), Yucatan State, Mexico [52]; North Atlantic Ocean, 2 mi SW North Rock, Bermuda as *Diplectanum bonaci* (nomen nudum) (Mizelle & Wood, unpublished); Parque Nacional Arrecife Alacranes, Yucatan, Mexico (22°22′ 41″ N, 89°30′ 57″ W) [9]; Reserva de la Biosfera Ria Celestún, Yucatan, Mexico (20°51′ 34″ N, 90°24′ 69″ W) [9]; Laguna las Marites, Isla de Margarita, Venezuela, as *Diplectanum* sp. [12].

Infection site: Gill lamellae.

Minimum Prevalence: 100% (one specimen examined and infected).

Specimens studied: 10 voucher specimens, USNM 1273882, MNHN HEL534–535, FSBC-I 127763, 127764.

Museum specimens examined: Three paratypes of *P. capurroi*, USNPC 87300; holotype, two paratypes of *Diplectanum bonaci* (nomen nudum), USNPC 72744, 72745.

#### Redescription (Figs. 33–39)

Measurements (dimensions of the paratypes and specimens from Bermuda, respectively, follow in brackets those of the voucher specimens from Florida): Squamodisc rows 14–15 (*n* = 5) [12–15 (*n* = 3); 14–15 (*n* = 3)]; ventral anchor length 39 (37–42; *n* = 10) [39 (37–42; *n* = 3); 35 (34–37; *n* = 3)]; dorsal-anchor length 34 (32–35; *n* = 6) [35–36 (*n* = 3); 30–31 (*n* = 1)]; ventral bar length 94 (90–103; *n* = 8) [106 (101–111; *n* = 2); 82 (72–88; *n* = 3)]; dorsal bar length 54 (52–59; *n* = 8) [62 (60–65; *n* = 2); 52 (48–56; *n* = 3)]; hook length 11–12 (*n* = 2) [11–12 (*n* = 3); 11–12 (*n* = 7)]; MCO 142 (134–151; *n* = 8).

**Figures 33–39.**
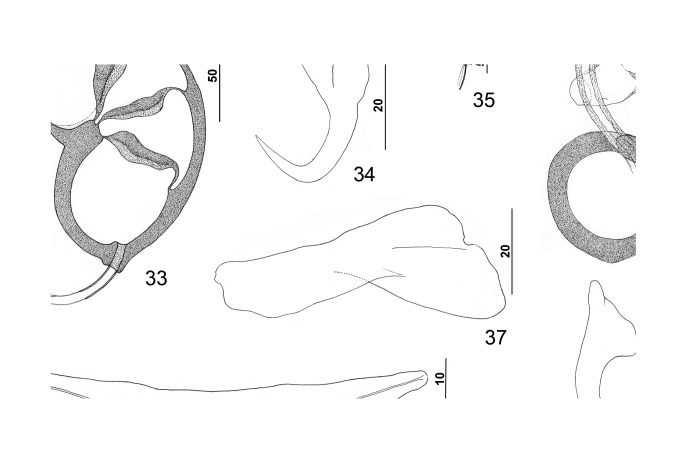
*Pseudorhabdosynochus capurroi* Vidal-Martínez & Mendoza-Franco, 1998 from black grouper *Mycteroperca bonaci*. 33: male copulatory organ (ventral view); 34: ventral anchor; 35: hook; 36: vaginal vestibule and sclerite (dorsal view); 37: right dorsal bar (ventral view); 38: ventral bar; 39: dorsal anchor. All drawings are from the available type specimens except Figure 33 (voucher specimen).

#### Remarks

This species was collected in 1973 from *M. bonaci* in Bermuda by Mizelle and Wood (USNPC Specimen Log for 72744, 72745) prior to the discovery of the Mexican specimens on which the original description was based. Named *Diplectanum bonaci* (nomen nudum) by Mizelle and Wood, the description of the species was never published. Comparison of Mizelle & Wood’s specimens with the available paratypes of *P. capurroi* confirmed the conspecificity of the two collections. Examination of a black grouper housed in the FSBC and collected by FWC/FWRI personnel in 1967 provided the 10 specimens identified herein as *P. capurroi*. Although of sufficient quality to determine their specific identity, the latter specimens had severely contracted which precluded determination of many characters associated with their reproductive systems and the possibility of providing a redescription and a whole-mount figure of the species. Nonetheless, the species is easily differentiated from all congeners by the morphology of its dorsal bars, which Vidal-Martínez & Mendoza-Franco [52] described as being “twisted”. The morphology of the anchors, ventral bar, hooks, and vaginal sclerite is nearly identical to that of *P. kritskyi*, which differs most significantly from *P. capurroi* by possessing dorsal bars with enlarged bilobed medial ends and lacking the twisted nature of those of *P. capurroi*.

Espínola-Novelo et al. [9] indicated that two species of *Pseudorhabdosynochus* occurred on *M. bonaci* off the Yucatan Peninsula, Mexico, near the type locality of *P. capurroi*. While no evidence is available that would suggest that the original description [52] was based on specimens representing two distinct species as occurred in the original description of *P. yucatanensis*, confirmation of the identity of *P. capurroi* may depend on examination of its holotype if another species of *Pseudorhabdosynochus* is verified from *M. bonaci* in the region (see Remarks for *P. yucatanensis* and *P. justinella*).

### *Pseudorhabdosynochus hyphessometochus* n. sp.

urn:lsid:zoobank.org:act:4951C92F-C406-4783-B598-C3546FA92F71

Type host and locality: Yellowmouth grouper, *Mycteroperca interstitialis* (Poey) (Serranidae: Epinephelinae: Epinephelini) (FSBC 7686): ~115 mi NW of Tampa, Florida (28.453° N, 84.217° W), May 20, 1973.

Other record: *Mycteroperca interstitialis* (FSBC 12022): ~135 mi SW of Galveston, Texas (27°49′ 59″ N, 93°19′ 59″ W), March 31, 1981.

Infection site: Gill lamellae.

Minimum prevalence: 100% (2 of 2 yellowmouth grouper infected).

Specimens studied: Holotype, USNM 1273679; 29 paratypes, USNM 1273680, 1273681, NHMUK 2015.2.25.3–4, MNHN HEL532–533, FSBC-I 127765, 127766.

Etymology: The specific name (a noun) is from Greek (*hyphesson* = somewhat smaller + *metochos* = a companion) and refers to the species being a member of the group of similar species of *Pseudorhabdosynochus* parasitizing groupers assigned to *Mycteroperca* and having a comparatively small cavity within the chamber of the vaginal sclerite.

#### Description (Figs. 40–48)

Body flattened dorsoventrally, with broad cephalic region, trunk with nearly parallel lateral margins, and moderately long peduncle tapering posteriorly. Tegumental scales absent. Cephalic region with terminal and two bilateral poorly developed lobes; three pairs of head organs; pair of bilateral groups of cephalic-gland cells at level of pharynx. Two pairs of eyespots anterior to pharynx lacking lenses; chromatic granules small, irregular in outline; accessory granules usually absent in cephalic region. Pharynx subspherical; esophagus short to nonexistent; intestinal ceca blind, extending posteriorly into anterior portion of peduncle. Haptor with dorsal and ventral anteromedial lobes containing respective squamodiscs and lateral lobes having hook pairs 2–4, 6, 7. Squamodiscs subequal, with 12 or 13 concentric U-shaped rows of rodlets; innermost rows of ventral squamodisc (three) and dorsal squamodisc (two) closed, forming ovals. Ventral anchor with short superficial root, deep root with small lateral swelling, slightly curved to straight shaft, and recurved point extending to level of tip of superficial root. Dorsal anchor with subtriangular base, short roots, curved shaft, and recurved point extending past level of tip of superficial root. Ventral bar with deep medial constriction, tapered ends, longitudinal ventral groove. Paired dorsal bar with spatulate medial end. Hook with depressed thumb, delicate point, uniform shank; FH loop about shank length. Testis subspherical; proximal vas deferens not observed; seminal vesicle a slight dilation of vas deferens; distal vas deferens entering elongate thick-walled ejaculatory bulb; ejaculatory duct entering MCO through portal of proximal chamber. A second duct of unknown origin and function entering portal of MCO. MCO reniform, quadriloculate, with short tapered cone; walls of chambers comparatively thick; distal tube elongate; retractile filament not observed. Germarium pyriform, dorsoventrally looping right intestinal cecum; Mehlis’ gland not observed; uterus delicate, with variable diameter. Vaginal sclerite with distal tube having single recurve before its attachment to vaginal vestibule; pear-shaped chamber with thick walls and small cavity; vaginal canal and seminal receptacle not observed. Vitellarium absent in regions of other reproductive organs, otherwise dense throughout trunk and extending into anterior portion of peduncle. Egg elongate ovate, lacking filaments.

**Figures 40–48.**
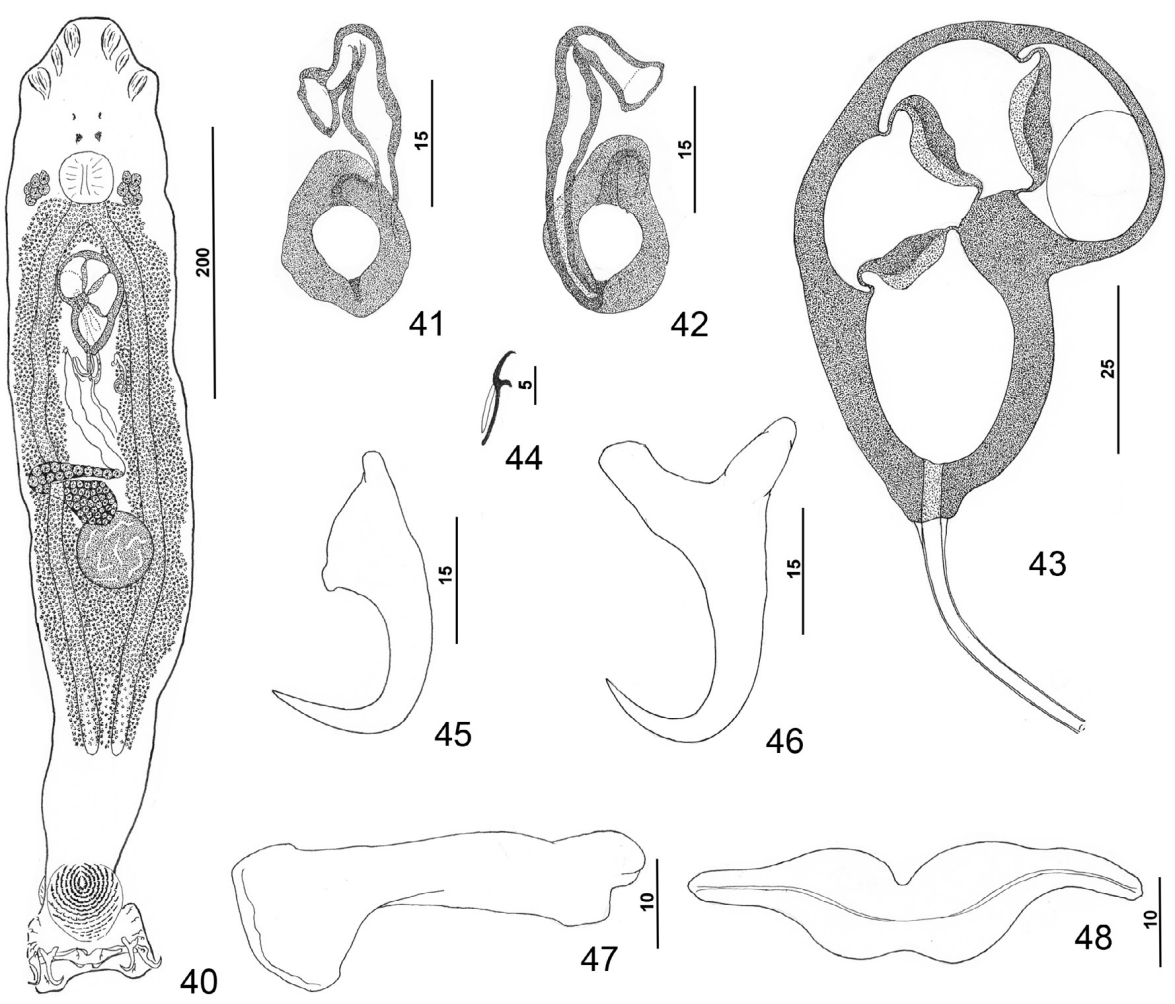
*Pseudorhabdosynochus hyphessometochus* n. sp. from yellowmouth grouper *Mycteroperca interstitialis*. 40: whole mount (composite, ventral view; dorsal squamodisc and dorsal anteromedial haptoral lobe not shown); 41: vaginal sclerite (ventral view); 42: vaginal sclerite (dorsal view); 43: male copulatory organ (dorsal view); 44: hook; 45: dorsal anchor; 46: ventral anchor; 47: left dorsal bar (ventral view); 48: ventral bar.

Measurements: Body 695 (567–896; *n* = 18) long; width at level of germarium 128 (87–166; *n* = 20). Haptor 132 (118–152; *n* = 14) wide; squamodisc 56 (50–67; *n* = 17) long, 52 (42–60; *n* = 18) wide. Ventral anchor 37 (35–39; *n* = 9) long; dorsal anchor 33 (31–36; *n* = 9) long. Ventral bar 55 (47–65; *n* = 9) long; dorsal bar 46 (42–56; *n* = 9) long. Hook 11 (10–12; *n* = 17) long. Pharynx 45 (39–52; *n* = 19) wide. MCO 72 (63–78; *n* = 19) long. Testis 65 (50–80; *n* = 7) long, 61 (49–71; *n* = 7) wide. Germarial bulb 41 (37–45; *n* = 4) wide. Egg 86–87 (*n* = 1) long, 35–36 (*n* = 1) wide.

#### Remarks

*Pseudorhabdosynochus hyphessometochus* n. sp. is a member of the apparently closely related group of *Pseudorhabdosynochus* species infecting most *Mycteroperca* species occurring in the western Atlantic region (see Remarks for *P. kritskyi* for a list of species). It most closely resembles *P. mycteropercae* n. sp. in the basic morphology of the MCO and haptoral sclerites but is distinguished from the latter species by having a vaginal sclerite with a smaller thick-walled chamber with a reduced cavity and a distal tube with a single recurve before its attachment to the vaginal vestibule (vaginal sclerite with a comparatively thinner wall and larger cavity of the chamber and a double recurve of the distal tube in *P. mycteropercae*).

### *Pseudorhabdosynochus sulamericanus* Santos, Buchmann & Gibson, 2000

Type host and locality: Snowy grouper, *Epinephelus niveatus* (Valenciennes) [now *Hyporthodus niveatus* (Valenciennes)] (Serranidae: Epinephelinae: Epinephelini): off Ilhas Cagarras, Rio de Janeiro, Brazil (23°02′ S, 43°12′ W).

Current records: *Hyporthodus niveatus*: ~40 mi E of Cape Canaveral, Florida (28.400–28.436° N, 80.014–80.019° W), April 26, 2011; open Gulf of Mexico, ~60 mi SW of Pensacola, Florida (29.730° N, 86.621° W), July 16, 2011; open Gulf of Mexico, ~130 mi SW of Pensacola, Florida (29.139° N, 85.615° W), July 14, 2011 (new locality records). Warsaw grouper, *Hyporthodus nigritus* (Holbrook): open Gulf of Mexico, ~160 mi W of Naples, Florida (26.167° N, 84.500° W), June 20, 2012 (new host and locality records).

Previous records: There are no previous records other than that of the original description [46].

Infection site: Gill lamellae.

Minimum prevalences: 100% (8 of 8 snowy groupers and 1 of 1 Warsaw grouper infected).

Specimens studied: 56 voucher specimens from *H. niveatus*, USNM 1276179–1276184, NHMUK 2014.11.14.9–10, MNHN HEL458–461, FSBC-I 127742, 127743; 9 voucher specimens from *H. nigritus*, USNM 1276178.

Museum specimen examined: Paratype, NHMUK 1999.1.6.1–3.

#### Redescription (Figs. 49–56)

Body dorsoventrally flattened. Tegumental scales with rounded anterior margins extending from peduncle anteriorly into posterior trunk. Cephalic region broad, with terminal and two bilateral poorly developed lobes, three bilateral pairs of head organs, pair of bilateral groups of cephalic-gland cells at level of pharynx. Two pairs of eyespots lacking lenses immediately anterior to pharynx; one to all eyespots poorly defined, apparently replaced by dissociated chromatic granules; accessory chromatic granules small, irregular in outline, usually present in cephalic region. Pharynx subspherical to subovate; esophagus short to nonexistent; intestinal ceca blind, extending posteriorly to near peduncle. Peduncle broad. Haptor with dorsal and ventral anteromedial lobes containing respective squamodiscs and lateral lobes having hook pairs 2–4, 6, 7. Squamodiscs subequal, with 14–17 (usually 15) U-shaped rows of rodlets; innermost row teardrop shaped, closed. Ventral anchor with short superficial root, longer deep root having lateral swelling, slightly curved shaft, and recurved point extending just past level of tip of superficial root. Dorsal anchor with subtriangular base, superficial root short to lacking, short deep root, slightly arcing shaft, recurved point extending past level of tip of superficial root. Ventral bar with medial constriction, tapered ends, longitudinal medioventral groove. Paired dorsal bar with spatulate medial end. Hook with long slightly depressed thumb, delicate point, uniform shank; FH loop nearly shank length. Testis ovate, lying sinistroposterior to germarium along body midline; proximal vas deferens not observed; seminal vesicle a simple dilation of distal vas deferens, lying posterior to MCO; ejaculatory bulb and duct not observed; large vesicle (prostatic reservoir?) lying dextral to distal chamber of MCO. MCO quadriloculate, with thick walls, short distal cone, elongate tube, protruding filament variable in length. Germarium pyriform; germarial bulb lying diagonally at body midlength, with dorsoventral distal loop around right intestinal cecum; ootype lying to left of body midline, with well-developed Mehlis’ gland; uterus delicate, banana shaped when empty. Common genital pore ventral, dextral to MCO. Vaginal pore sinistroventral at level of distal end of MCO; vaginal vestibule delicate; vaginal sclerite complex, with distal flare, irregular tube with small proximal bulge and surrounded by variable small sclerites, and small chamber giving rise to delicate vaginal canal. Seminal receptacle subspherical, immediately proximal to vagina and anterior to ootype. Bilateral vitelline ducts not observed; vitellarium absent in regions of other reproductive organs, otherwise dense throughout trunk.

**Figures 49–56.**
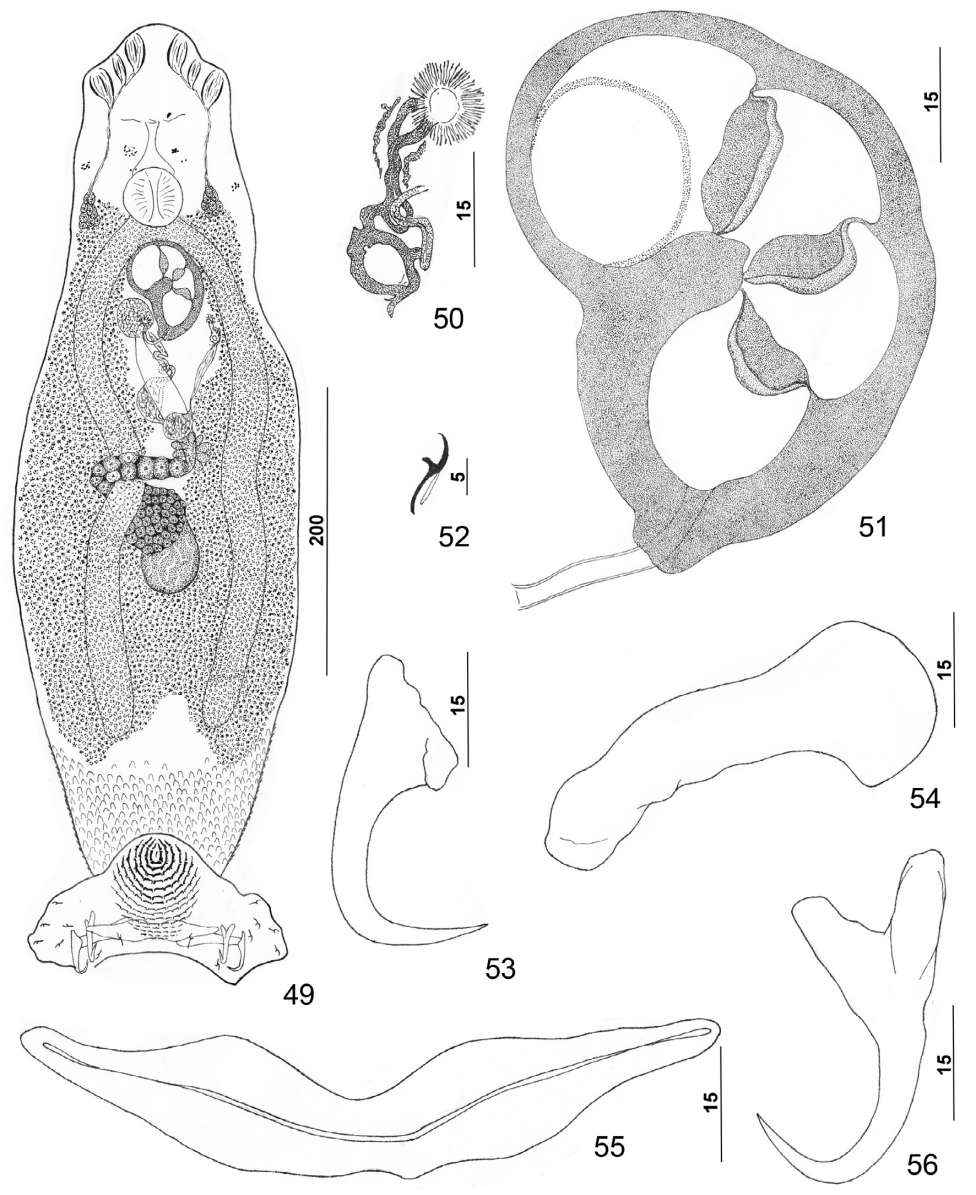
*Pseudorhabdosynochus sulamericanus* Santos, Buchmann & Gibson, 2000 from snowy grouper *Hyporthodus niveatus*. 49: whole mount (composite, ventral view; dorsal squamodisc and dorsal anteromedial haptoral lobe not shown); 50: vaginal sclerite (ventral view); 51: male copulatory organ (ventral view); 52: hook; 53: dorsal anchor; 54: right dorsal bar (ventral view); 55: ventral bar; 56: ventral anchor.

Measurements (dimensions of specimens from the Warsaw grouper, *Hyporthodus nigritus*, follow in brackets those from the type host, *H. niveatus*, respectively): Body 542 (460–649; *n* = 21) [879–880 (*n* = 1)] long; width at level of germarium 170 (137–201; *n* = 22) [179–180 (*n* = 1)]. Haptor 160 (131–180; *n* = 21) [165–166 (*n* = 1)] wide; squamodisc 72 (61–79; *n* = 18) [47–48 (*n* = 1)] long, 71 (63–81; *n* = 21) [80–81 (*n* = 1)] wide. Ventral anchor 41 (38–45; *n* = 17) [48 (47–50; *n* = 5)] long; dorsal anchor 40 (38–43; *n* = 18) [47 (46–49; *n* = 5)] long. Ventral bar 88 (82–97; *n* = 14) [83 (80–87; *n* = 5)] long; dorsal bar 60 (52–65; *n* = 18) [65 (58–69; *n* = 6)] long. Hook 12 (11–13; *n* = 26) [12 (11–13; *n* = 10)] long. Pharynx 38 (34–43; *n* = 22) [45–46 (*n* = 1)] wide. MCO 71 (65–79; *n* = 28) [74–75 (*n* = 1)] long. Testis 61 (53–74; *n* = 9) [71–72 (*n* = 1)] long, 55 (44–69; *n* = 11) [49–50 (*n* = 1)] wide. Germarial bulb 39 (34–45; *n* = 13) [50–51 (*n* = 1)] wide.

#### Remarks

The original description of *P. sulamericanus* [46] was based on 11 of 22 specimens they observed parasitizing a single snowy grouper from southern Brazil. The helminths collected from snowy and Warsaw groupers off Florida during the present study generally correspond to the original description, except that Santos et al. [46] reported that the “vas deferens enters the basal part of the cirrus-bulb [MCO], forming a large, round reservoir of the male accessory glands, which give an appearance of being an additional [5th] chamber of the proximal cirrus-bulb” (articles and brackets ours). Their statement is somewhat confusing because they implied that their so-termed reservoir is formed from the vas deferens but apparently serves as storage for product from the male accessory glands (prostate glands?).

Examination of the larger collection of *P. sulamericanus* from the hosts off Florida revealed that this “round reservoir of the male accessory glands” is actually a large portal in the dorsal wall of the proximal chamber of the MCO through which the distal ducts of the male reproductive system enter the organ. Santos et al. [46] apparently mistook the rim of the portal for walls of the reservoir of the male accessory glands. The portal is not unique to *P. sulamericanus*. It has been observed on all species described herein for which full descriptions or redescriptions are presented. The portal is usually not visible in individual specimens in which the wall of the proximal chamber of the MCO has collapsed.

*Pseudorhabdosynochus sulamericanus* is most similar to *P. firmicoleatus* n. sp. based on comparative morphology of the vaginal sclerite, MCO, and haptoral elements. The vaginal sclerite of both species has a comparatively small basal chamber and a small secondary bulge of the tube that flares at its union with the vaginal vestibule. The sclerite of *P. sulamericanus*, however, differs from that of *P. firmicoleatus* by having a slightly larger chamber and irregular sclerites associated with the distal tube.

### *Pseudorhabdosynochus firmicoleatus* n. sp.

urn:lsid:zoobank.org:act:BDE4BECE-5285-4E4D-9577-6E7F4316F8B2

Type host and locality: Yellowedge grouper, *Hyporthodus flavolimbatus* (Poey) (Serranidae: Epinephelinae: Epinephelini): open Gulf of Mexico, ~70 mi S of Panama City, Florida (29.139° N, 85.615° W), July 14, 2011.

Other record: Snowy grouper, *Hyporthodus niveatus*: open Gulf of Mexico, ~60 mi SW of Pensacola, Florida (29.730° N, 86.621° W), July 16, 2011.

Infection site: Gill lamellae.

Minimum prevalences: 100% (4 of 4 yellowedge grouper infected); 25% (2 of 8 snowy grouper infected).

Specimens studied: Holotype, USNM 1276213; 29 paratypes, USNM 1276214, NHMUK 2014.11.14.29–30, MNHN HEL496–498, FSBC-I 127753, 127754; 19 voucher specimens from *H. niveatus*, USNM 1276212.

Etymology: The specific name (an adjective) is from Latin (*firm/i* = firm + *cole/o* = sheath +-*atus* = marked by having) and refers to the heavy thick-walled chambers of the MCO.

#### Description (Figs. 57–72)

Based on specimens from the yellowedge grouper, *Hyporthodus flavolimbatus*: Body flattened dorsoventrally. Tegument smooth, lacking tegumental scales. Cephalic region broad, with terminal and two bilateral poorly developed lobes, three bilateral pairs of head organs, pair of bilateral groups of cephalic-gland cells at level of pharynx. Four eyespots immediately anterior to pharynx lacking lenses; members of posterior pair larger, closer together (infrequently adjoined) than those of anterior pair; accessory chromatic granules small, irregular in outline, infrequent in cephalic region. Pharynx subspherical to subovate, muscular; esophagus short to nonexistent; intestinal ceca blind, extending posteriorly to near peduncle, diverging posterior to testis. Peduncle broad, tapered posteriorly. Haptor subtrapezoidal to subtriangular, with dorsal and ventral anteromedial lobes containing squamodiscs and lateral lobes having hook pairs 2–4, 6, 7. Squamodiscs subequal, with 11–13 (usually 12) U-shaped rows of rodlets; ends of innermost teardrop-shaped row closed. Ventral anchor with short superficial root, slightly longer deep root usually directed dorsally and having small lateral swelling, shaft slightly curved, and recurved point extending just past level of tip of superficial root. Dorsal anchor with subtriangular base, superficial root short and directed posteriorly, deep root knoblike, shaft forming gentle arc, recurved point extending past level of tip of superficial root. Ventral bar with medial constriction, tapered ends, longitudinal medioventral groove. Paired dorsal bar with spatulate medial end. Hook with elongate slightly depressed thumb, delicate point, uniform shank; FH loop nearly shank length. Testis ovate to subspherical, postgermarial; proximal vas deferens not observed; seminal vesicle an indistinct dilation of distal vas deferens, lying on left side of body posterior to MCO; ejaculatory bulb small; ejaculatory duct entering portal of MCO; large vesicle (prostatic reservoir?) with reticulate contents lying dextral to distal chamber of MCO. MCO reniform, quadriloculate, with thick walls, variable short cone, moderately long distal tube, and retractile filament variable in length. Germarium pyriform; germarial bulb lying diagonally at midlength of trunk, with elongate dorsoventral distal loop around right intestinal cecum; ootype lying near body midline, surrounded by well-developed Mehlis’ gland, giving rise to delicate banana-shaped uterus when empty. Common genital pore ventral, dextral to MCO. Vaginal pore sinistroventral at level of seminal vesicle; vaginal sclerite with distal funnel, distal tube having a proximal bulge, and small chamber giving rise to delicate vaginal canal. Seminal receptacle anterior to ootype. Bilateral vitelline ducts not observed; vitellarium absent in regions of other reproductive organs, otherwise dense throughout trunk.

Measurements (dimensions of specimens from *H. niveatus* follow in brackets those from *H. flavolimbatus*, respectively): Body 443 (364–575; *n* = 17) [475 (466–483; *n* = 3)] long; width at level of germarium 111 (82–166; *n* = 17) [115 (95–128; *n* = 3)]. Haptor 155 (129–172; *n* = 17) [141 (135–148; *n* = 2)] wide; squamodisc 49 (44–52; *n* = 16) [49 (37–57; *n* = 3)] long, 49 (42–57; *n* = 17) [52 (50–54; *n* = 3)] wide. Ventral anchor 36 (35–39; *n* = 13) [35 (32–37; *n* = 13)] long; dorsal anchor 38 (36–41; *n* = 13) [37 (36–39; *n* = 13)] long. Ventral bar 84 (76–92; *n* = 12) [81 (73–90; *n* = 11)] long; dorsal bar 59 (57–64; *n* = 13) [58 (55–62; *n* = 13)] long. Hook 11–12 (*n* = 16) [12 (11–13; *n* = 20)] long. Pharynx 30 (22–37; *n* = 14) [35 (32–37; *n* = 3)] wide. MCO 53 (47–63; *n* = 15) [53 (52–54; *n* = 2)] long. Testis 64 (51–74; *n* = 12) [57 (52–63; *n* = 2)] long, 55 (41–69; *n* = 12) [50 (47–53; *n* = 2)] wide. Germarial bulb 34 (28–39; *n* = 5) [33 (28–40; *n* = 3)] wide.

#### Remarks

This species most closely resembles *P. sulamericanus* in the general morphology of the vaginal sclerite. The structure in both species possesses a delicate distal funnel at its attachment to the vaginal vestibule, a proximal bulge of the distal tube, and a small chamber. In *P. firmicoleatus*, however, the vaginal sclerite is delicate and lacks the irregular sclerites along the distal tube typical of *P. sulamericanus*. It further differs from *P. sulamericanus* in that the deep root of the ventral anchor has its proximal end directed dorsally (deep root of ventral anchor *of P. sulamericanus* straight). Finally, tegumental scales are lacking and the squamodiscs of *P. firmicoleatus* have 11–13 (usually 12) rows of rodlets (tegumental scales present and 14–17 rows, usually 15, in the squamodics of *P. sulamericanus*).

No significant differences were observed between specimens of *P. firmicoleatus* collected from yellowedge and snowy groupers (compare Figs. 58–72). The differences in the cone of the MCO depicted in Figures 59 and 65 were not specific to specimens from the respective hosts, but occurred among specimens from both hosts.

**Figures 57–64.**
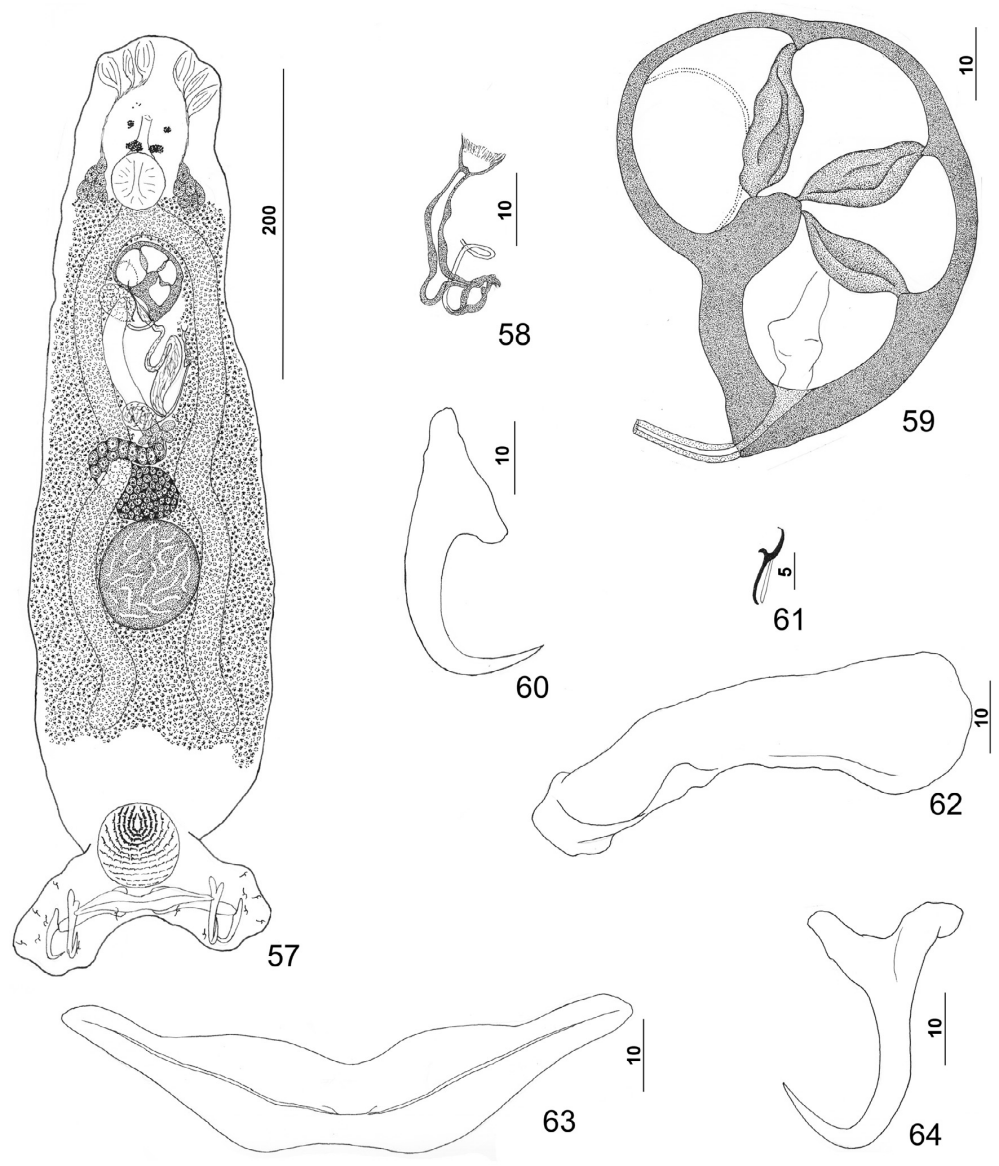
*Pseudorhabdosynochus firmicoleatus* n. sp. from yellowedge grouper *Hyporthodus flavolimbatus*. 57: whole mount (composite, ventral view; dorsal squamodisc and dorsal anteromedial haptoral lobe not shown); 58: vaginal sclerite (dorsal view); 59: male copulatory organ (ventral view); 60: dorsal anchor; 61: hook; 62: right dorsal bar (ventral view); 63: ventral bar; 64: ventral anchor.

**Figures 65–72.**
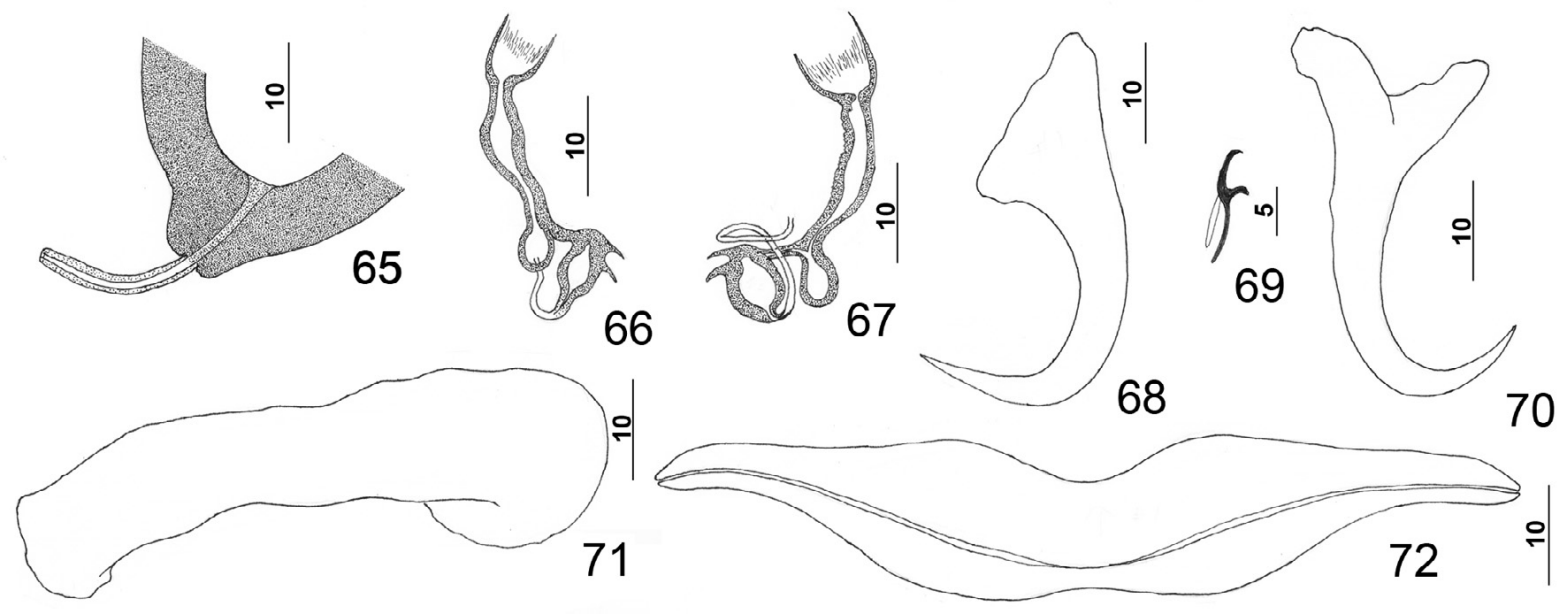
*Pseudorhabdosynochus firmicoleatus* n. sp. from snowy grouper *Hyporthodus niveatus*. 65: tip of distal chamber, cone, and distal tube of MCO (ventral view); 66: vaginal sclerite (dorsal view); 67: vaginal sclerite (ventral view); 68: dorsal anchor; 69: hook; 70: ventral anchor; 71: right dorsal bar (ventral view); 72: ventral bar.

### *Pseudorhabdosynochus mcmichaeli* n. sp.

urn:lsid:zoobank.org:act:B472E4AE-9F31-409B-A16C-10A442E13904

Type host and locality: Scamp, *Mycteroperca phenax* Jordan and Swain (Serranidae: Epinephelinae: Epinephelini): Florida Middle Grounds, Gulf of Mexico (28.269–28.615° N, 84.127–84.334° W), May 5–11, 2009.

Other record: *Mycteroperca phenax*: Gulf of Mexico, ~25 mi west of Tampa Bay, Florida (27.522° N, 83.088° W), October 18, 2008.

Infection site: Gill lamellae.

Minimum prevalence: 71% (10 of 14 scamp infected).

Specimens studied: Holotype, USNM 1276196; 34 paratypes, USNM 1276197–1276203, NHMUK 2014.11.14.21–22, MNHN HEL481–483, FSBC-I 127726, 127727; 3 voucher specimens, USNM 1276204.

Etymology: This species is named for Robert (Bob) McMichael, Jr., Founding Director of the FWC/FWRI Fisheries Independent Monitoring Program, in honor of his decades of support for research on the parasites and diseases affecting Florida’s marine fishes. Specimens of groupers he collected in 1980, the first year he was employed by the FWC (then the Florida Department of Natural Resources), are among those we evaluated for this study, and until he retired as program director in 2014, he actively, unhesitatingly, and enthusiastically accommodated our requests for host specimens and our participation in field sampling.

#### Description (Figs. 73–80)

Body flattened dorsoventrally. Tegumental scales small, with rounded anterior margins, extending from peduncle anteriorly into posterior trunk. Cephalic region broad, with terminal and two bilateral poorly developed lobes, three bilateral pairs of head organs, pair of bilateral groups of cephalic-gland cells at level of pharynx. Four eyespots lacking lenses anterior to pharynx; members of posterior pair larger and slightly closer together than those of anterior pair; accessory chromatic granules small, irregular in outline, infrequent or absent in cephalic region. Pharynx subovate, muscular; esophagus short to nonexistent; intestinal ceca blind, extending posteriorly to peduncle. Peduncle broad, tapered posteriorly. Haptor subtriangular, with dorsal and ventral anteromedial lobes containing respective squamodiscs and lateral lobes having hook pairs 2–4, 6, 7. Squamodiscs subequal, with 11–13 (usually 12) concentric U-shaped rows of rodlets; innermost two rows usually closed, teardrop shaped. Ventral anchor with comparatively short superficial root, elongate deep root having lateral swelling, slightly curved shaft and short recurved point extending to just past the level of tip of superficial root. Dorsal anchor with subtriangular base, superficial root short to lacking, deep root knoblike, shaft forming an arc, recurved point extending past level of tip of superficial root. Ventral bar with slight medial constriction, long tapered ends, longitudinal medioventral groove. Paired dorsal bar with spatulate medial end. Hook with elongate slightly depressed thumb, delicate point, uniform shank; FH loop nearly shank length. Testis subspherical, lying sinistroposterior to germarium; common genital pore ventral, dextral to MCO. Proximal vas deferens not observed; seminal vesicle a small dilation of distal vas deferens, lying posterior to MCO; ejaculatory bulb and duct not observed; large vesicle (prostatic reservoir?) lying to right of distal chamber of MCO. MCO reniform, quadriloculate, with elongate tapered cone, delicate distal tube, and retractile filament variable in length; walls of chambers of MCO comparatively thin, becoming progressively thicker distally. Germarium pyriform; germarial bulb lying diagonally in posterior trunk, with dorsoventral distal loop around right intestinal cecum; ootype lying on or near body midline, with well-developed Mehlis’ gland; uterus delicate, banana shaped when empty. Vaginal pore sinistroventral at level of cone of MCO; vaginal vestibule delicate; vaginal sclerite with two tandem chambers having thin walls; delicate vaginal canal arising from posterior chamber, looping anteriorly before expanding slightly and extending posteriorly to seminal receptacle; seminal receptacle subspherical, lying near ootype and Mehlis’ gland. Bilateral vitelline ducts not observed; vitellarium absent in regions of other reproductive organs, otherwise dense throughout trunk.

**Figures 73–80.**
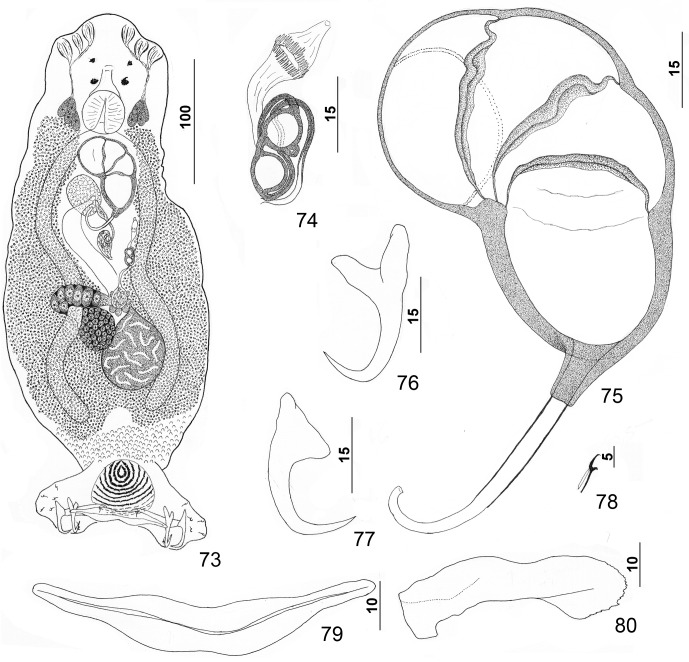
*Pseudorhabdosynochus mcmichaeli* n. sp. from scamp *Mycteroperca phenax*. 73: whole mount (composite, ventral view; dorsal squamodisc and dorsal anteromedial haptoral lobe not shown); 74: vaginal sclerite and vestibule (ventral view); 75: male copulatory organ (ventral view); 76: ventral anchor; 77: dorsal anchor; 78: hook; 79: ventral bar; 80: right dorsal bar (ventral view).

Measurements: Body 405 (350–473; *n* = 20) long; width at level of germarium 163 (127–185; *n* = 19). Haptor 139 (125–197; *n* = 19) wide; squamodisc 47 (40–53; *n* = 18) long, 54 (45–62; *n* = 18) wide. Ventral anchor 28 (26–29; *n* = 13) long; dorsal anchor 31 (28–33; *n* = 14) long. Ventral bar 74 (66–81; *n* = 10) long; dorsal bar 48 (44–52; *n* = 14) long. Hook 10 (9–12; *n* = 12) long. Pharynx 34 (29–38; *n* = 23) wide. MCO 68 (61–74; *n* = 17) long. Testis 63 (52–77; *n* = 19) long, 56 (43–72; *n* = 19) wide. Germarial bulb 33 (25–40; *n* = 17) wide.

#### Remarks

*Pseudorhabdosynochus mcmichaeli* n. sp. is easily distinguished from all congeners from western Atlantic waters by its unique vaginal sclerite. Within the region, it is probably most similar to the congeners infecting other *Mycteroperca* species. In *P. mcmichaeli*, however, the vaginal sclerite has two tandem chambers, each with comparatively thin walls, while all other species infecting *Mycteroperca* species, including *P. kritskyi*, *P. capurroi*, *P. vascellum* n. sp., *P. hyphessometochus*, *P. contubernalis* n. sp., and *P. mycteropercae* n. sp., possess a vaginal sclerite with a single comparatively thick-walled chamber.

Based on the similarities of the respective vaginal sclerites, *P. mcmichaeli* most closely resembles the recently described *Pseudorhabdosynochus jeanloui* Knoff, Cohen, Cárdenas, Cárdenas-Callirgos & Corrêa Gomes, 2015 from the Pacific creole-fish *Paranthias colonus* (Valenciennes) off Peru [22]. It differs from *P. jeanloui* by being a much smaller parasite [body length 405 (350–473) in *P. mcmichaeli*; 781 (600–930) in *P. jeanloui*], by having a less expanded base of the smaller ventral anchor and tapered ends of the ventral bar, and in the fine details of the vaginal sclerite.

### *Pseudorhabdosynochus meganmarieae* n. sp.

urn:lsid:zoobank.org:act:A7B62D31-CF89-4264-B29C-0AB08EF2E0D2

Type host and locality: Graysby, *Cephalopholis cruentata* (Lacepède) (Serranidae: Epinephelinae: Epinephelini): Florida Middle Grounds, Gulf of Mexico (28.536–28.561° N, 84.263–84.271° W), October 2 and 3, 2009.

Infection site: Gill lamellae.

Minimum prevalence: 100% (3 of 3 hosts infected).

Specimens studied: Holotype, USNM 1276215; 23 paratypes, USNM 1276216, 1276217, NHMUK 2014.11.14.31–32, MNHN HEL499–501, FSBC-I 127755, 127756.

Etymology: This species is named for Megan-Marie (Duckworth) Bakenhaster, who helped to make this study possible by spending weeks and innumerable late nights isolated from adult company while caring for a young, singular child possessed of strong opinions on the limits of parental authority. Her patience and perseverance facilitated our collections of specimens of this and other species of *Pseudorhabdosynochus* reported herein.

#### Description (Figs. 81–88)

Body elongate ovate, flattened dorsoventrally. Tegument smooth, lacking tegumental scales. Cephalic region broad, with terminal and two bilateral poorly developed lobes, three bilateral pairs of head organs, pair of bilateral groups of cephalic-gland cells at level of pharynx. Four eyespots lacking lenses, lying anterior to pharynx; members of posterior pair larger, closer together than those of anterior pair; accessory chromatic granules small, irregular in outline, uncommon or absent in cephalic region. Pharynx subovate; esophagus short to nonexistent; intestinal ceca blind, extending posterior to anterior limit of peduncle. Peduncle broad, tapered posteriorly. Haptor subtriangular, with dorsal and ventral anteromedial lobes containing respective squamodiscs and lateral lobes having hook pairs 2–4, 6, 7; squamodiscs subequal, with 11–13 (usually 12) U-shaped rows of rodlets; 2–3 innermost rows circular, closed. Ventral anchor with subequal comparatively short superficial and deep roots; deep root with slight lateral swelling; shaft long straight; short recurved point extending to level of tip of superficial root. Dorsal anchor with subtriangular base, superficial root short to lacking, deep root knoblike, comparatively long shaft forming gentle arc, recurved point extending past level of tip of superficial root. Ventral bar with deep medial constriction, elongate tapered ends, longitudinal medioventral groove. Paired dorsal bar with spatulate medial end. Hook with long slightly depressed thumb, delicate point, uniform shank; FH loop nearly shank length. Testis subspherical, lying dorsoposterior to germarium; proximal vas deferens not observed; seminal vesicle an indistinct dilation of distal vas deferens, lying just posterior to MCO; ejaculatory bulb and duct not observed: large vesicle (prostatic reservoir?) with lightly staining reticulate contents dextral of distal chamber of MCO. MCO reniform, quadriloculate, with long tapered cone, moderately long distal tube, and variable retractile filament often not observed; chamber walls of MCO comparatively thin, delicate, often collapsed. Germarium pyriform; germarial bulb lying diagonally at midlength of trunk, with elongate distal loop dorsoventrally around right intestinal cecum; ootype lying on or near body midline, with well-developed Mehlis’ gland; uterus delicate, banana shaped when empty. Common genital pore ventral, dextral to MCO. Vaginal pore sinistroventral at level of cone of MCO; vaginal vestibule delicate; vaginal sclerite funnel shaped, with heavy walls and two small juxtaposed chambers. Seminal receptacle subspherical immediately proximal to vagina and anterior to ootype. Bilateral vitelline ducts not observed; vitellarium absent in regions of other reproductive organs, otherwise dense throughout trunk and anterior portion of peduncle.

**Figures 81–88.**
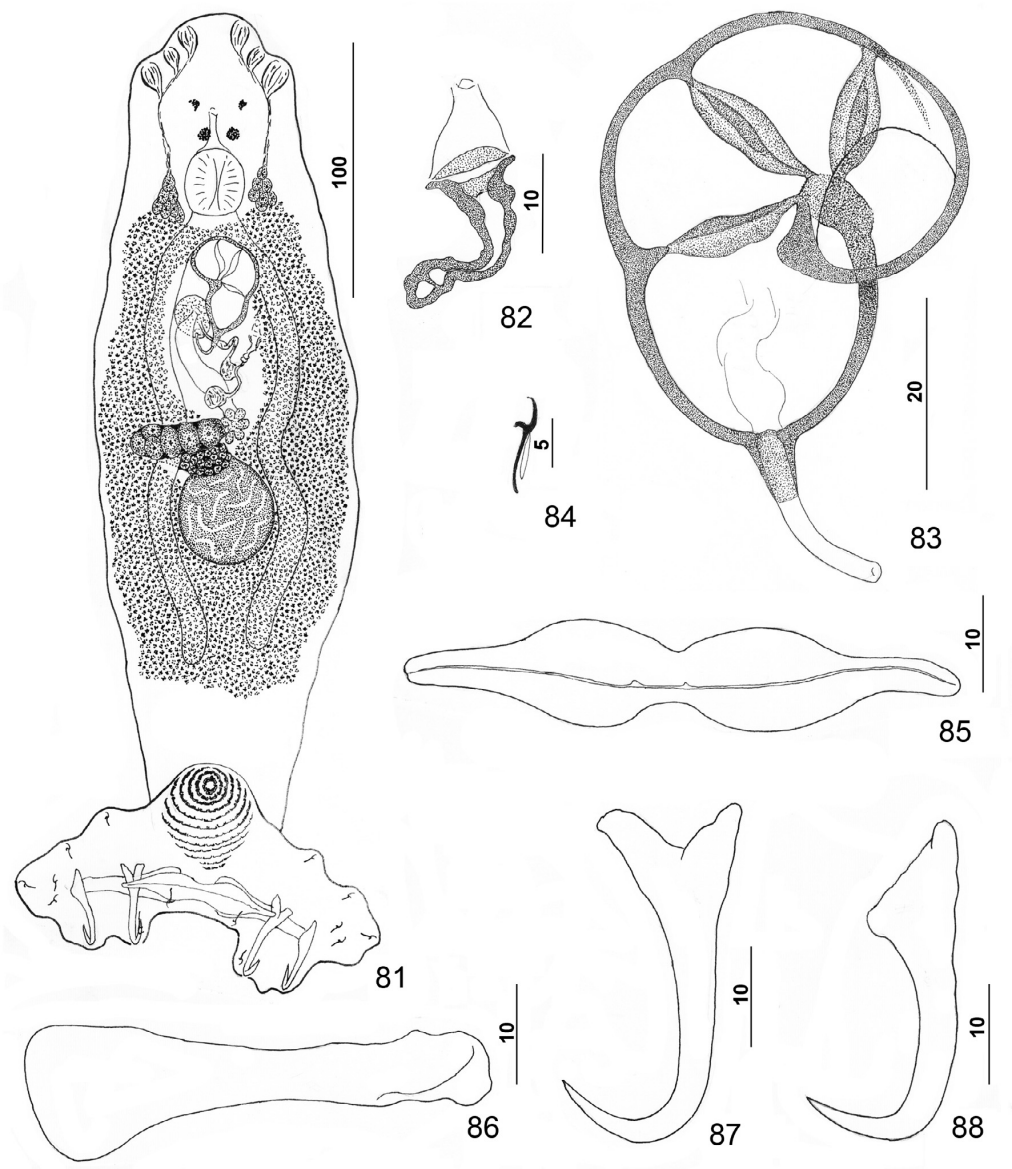
*Pseudorhabdosynochus meganmarieae* n. sp. from graysby *Cephalopholis cruentata*. 81: whole mount (composite, ventral view; dorsal squamodisc and dorsal anteromedial haptoral lobe not shown); 82: vaginal vestibule and sclerite (ventral view); 83: male copulatory organ (dorsal view); 84: hook; 85: ventral bar; 86: left dorsal bar (ventral view); 87: ventral anchor; 88: dorsal anchor.

Measurements: Body 366 (327–399; *n* = 7) long; width at level of germarium 103 (85–124; *n* = 8). Haptor 127 (105–146; *n* = 7) wide; squamodisc 41 (32–47; *n* = 6) long, 39 (34–46; *n* = 6) wide. Ventral anchor 36 (34–39; *n* = 15) long; dorsal anchor 36 (33–39; *n* = 14) long. Ventral bar 57 (54–62; *n* = 13) long; dorsal bar 52 (48–56; *n* = 15) long. Hook 12 (11–13; *n* = 21) long. Pharynx 27 (25–29; *n* = 8) wide. MCO 44 (42–47; *n* = 8) long. Testis 40 (38–47; *n* = 8) long, 40 (36–45; *n* = 8) wide. Germarial bulb 22 (20–24; *n* = 5) wide.

#### Remarks

*Pseudorhabdosynochus meganmarieae* n. sp. is easily distinguished from all other known congeners from the western Atlantic region by having ventral anchors with short roots (superficial root slightly longer than deep root) and an elongate straight shaft. Based on the presence of two small chambers and a distal funnel-shaped tube in the vaginal sclerites, *P. meganmarieae* most closely resembles *P. yucatanensis*, but the vaginal sclerite in *P. meganmarieae* is more robust and its distal end has a more pronounced funnel shape than that of *P. yucatanensis*. It differs further from *P. yucatanensis* by lacking tegumental scales (scales present in *P. yucatanensis*) and by having a comparatively deep medial constriction in the ventral bar (medial constriction of the ventral bar minimal in *P. yucatanensis*) and a tapered cone and thinner walls of the chambers of the MCO (cone cylindrical and walls of the three distal chambers noticeably thicker in *P. yucatanensis*).

### *Pseudorhabdosynochus vascellum* n. sp.

urn:lsid:zoobank.org:act:C5CF5C5C-CB56-4CEE-8E4F-67F0E82849B2

Type host and locality: Scamp, *Mycteroperca phenax* Jordan & Swain (Serranidae: Epinephelinae: Epinephelini): Pinnacles Reef System off Mississippi Sound, S of Pascagoula, Mississippi (~30°00′ N, 88°30′ W), May 4, 2008.

Infection site: Gill lamellae.

Minimum prevalence: 7% (1 of 14 *M. phenax* infected).

Specimens studied: Holotype, USNM 1276205; 28 paratypes, USNM 1276206, NHMUK 2014.11.14.23–24, MNHN HEL484–487, FSBC-I 127757, 127758.

Etymology: The specific name (a noun) is from Latin (*vascellum* = a small vessel) and refers to the small chamber of the vaginal sclerite.

#### Description (Figs. 89–96)

Body flattened dorsoventrally, gently tapering anteriorly from level of testis. Tegument smooth, lacking tegumental scales. Cephalic region broad, with terminal and two bilateral poorly developed lobes, three bilateral pairs of head organs, pair of bilateral groups of cephalic-gland cells at level of pharynx. Four eyespots anterior to pharynx lacking lenses, individual eyespots occasionally dissociated; members of posterior pair larger, closer together than those of anterior pair; accessory chromatic granules small, subspherical, infrequent or absent in cephalic region. Mouth ventral along body midline at level of or slightly anterior to eyespots; pharynx subspherical to subovate, muscular; esophagus short; intestinal ceca blind, extending posteriorly to anterior limit of peduncle. Peduncle broad, tapered posteriorly. Haptor subtrapezoidal, with dorsal and ventral anteromedial lobes containing respective squamodiscs and lateral lobes having hook pairs 2–4, 6, 7. Squamodiscs subequal, with 11–12 (usually 12) U-shaped rows of rodlets; ventral squamodisc with three innermost rows of rodlets closed, teardrop shaped; innermost two rows of rodlets of dorsal squamodisc closed, teardrop shaped. Ventral anchor with short superficial root, slightly longer deep root directed dorsally and having small lateral swelling, curved shaft, and recurved point extending to level of tip of superficial root. Dorsal anchor with subtriangular base, inconspicuous superficial root, knoblike deep root, gently curved shaft, recurved point extending past level of tip of superficial root. Ventral bar with medial constriction, tapered ends, longitudinal medioventral groove. Paired dorsal bar with spatulate medial end. Hook with elongate slightly depressed thumb, delicate point, uniform shank; FH loop nearly shank length. Testis subspherical, postgermarial, or dorsally overlapping posterior end of germarial bulb. Proximal vas deferens not observed; seminal vesicle a simple dilation of distal vas deferens, lying to left of body midline near vaginal sclerite; distal vas deferens with circular loop before entering small thick-walled ejaculatory bulb; ejaculatory duct entering MCO through portal of proximal chamber of MCO; large vesicle (prostatic reservoir?) with nearly clear contents lying to right of distal chamber of MCO. MCO reniform, quadriloculate, with distal chamber having thick wall, barely noticeable short cone, moderately long distal tube, and retractile filament variable in length. Germarium pyriform; germarial bulb lying on body midline, with distal dorsoventral loop around right intestinal cecum; ootype slightly sinistral or on body midline, surrounded by well-developed Mehlis’ gland, giving rise to delicate banana-shaped uterus when empty. Common genital pore ventral, dextral to distal chamber of MCO. Vaginal pore sinistroventral; vaginal vestibule delicate, with thin wall; vaginal sclerite having delicate tube with recurved and flared distal end, chamber small with thick wall and small inner cavity. Vaginal canal arising from vaginal sclerite and directed posteriorly to seminal receptacle lying on body midline anteroventral to Mehlis’ gland. Bilateral vitelline ducts not observed; vitellarium absent in regions of other reproductive organs, otherwise dense throughout trunk.

**Figures 89–96.**
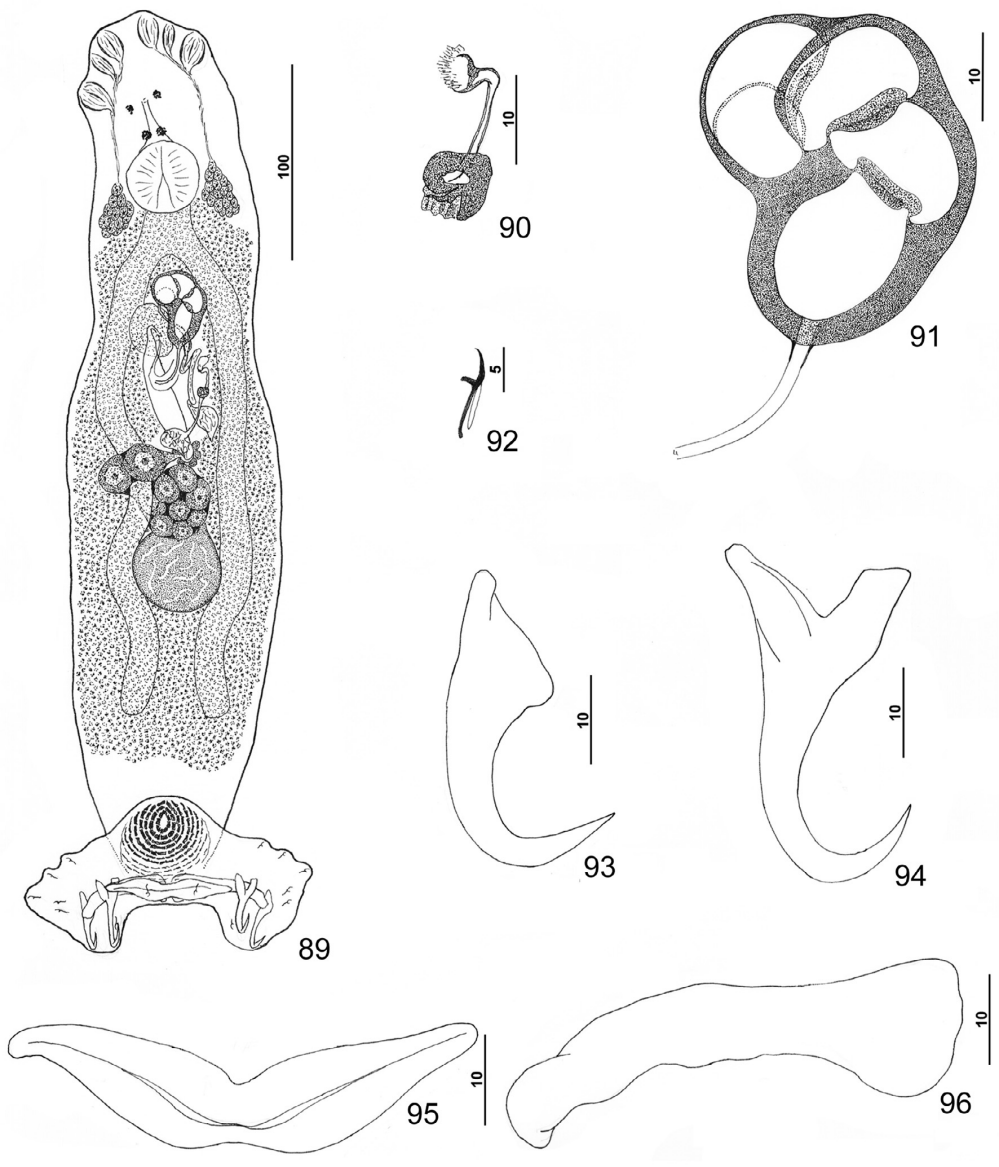
*Pseudorhabdosynochus vascellum* n. sp. from scamp *Mycteroperca phenax*. 89: whole mount (composite, ventral view; dorsal squamodisc and dorsal anteromedial haptoral lobe not shown); 90: vaginal sclerite (ventral view); 91: male copulatory organ (ventral view); 92: hook; 93: dorsal anchor; 94: ventral anchor; 95: ventral bar; 96: right dorsal bar (ventral view).

Measurements: Body 494 (419–580; *n* = 21) long; width at level of germarium 101 (80–123; *n* = 22). Haptor 144 (133–160; *n* = 18) wide; squamodisc 116 (104–127; *n* = 9) long, 119 (109–131; *n* = 9) wide. Ventral anchor 38 (36–39; *n* = 6) long; dorsal anchor 36 (34–37; *n* = 6) long. Ventral bar 58 (54–61; *n* = 5) long; dorsal bar 55 (52–58; *n* = 6) long. Hook 11 (10–12; *n* = 10) long. Pharynx 36 (31–41; *n* = 22) wide. MCO 43 (40–46; *n* = 22) long. Testis 45 (40–50; *n* = 18) long, 43 (34–50; *n* = 18) wide. Germarial bulb 35 (29–43; *n* = 20) wide.

#### Remarks

*Pseudorhabdosynochus vascellum* n. sp. belongs to the group of congeners parasitizing groupers assigned to *Mycteroperca* and characterized by having a distally reflexed tube and a single chamber in the vaginal sclerite. The group includes *P. kritskyi*, *P. capurroi*, *P. vascellum*, *P. contubernalis* n. sp., *P. hyphessometochus* n. sp., and *P. mycteropercae* n. sp. Except for *P. hyphessometochus*, *P. vascellum* is easily distinguished from the these species by its small thick-walled chamber of the vaginal sclerite having a meager cavity (chamber of the remaining species comparatively large and with a large cavity). *Pseudorhabdosynochus hyphessometochus* also possesses a vaginal sclerite with a thick wall of the chamber, but the cavity of the chamber in this species is comparatively large. The new species is probably closest morphologically to *P. contubernalis* by possessing a delicate, almost nonexistent cone of the MCO.

### *Pseudorhabdosynochus contubernalis* n. sp.

urn:lsid:zoobank.org:act:6B13BD66-0EF6-48D8-8C21-ECB253A62B61

Type host and locality: Scamp, *Mycteroperca phenax* Jordan & Swain (Serranidae: Epinephelinae: Epinephelini): Pinnacles Reef System off Mississippi Sound, S of Pascagoula, Mississippi (~30°00′ N, 88°30′ W), May 4, 2008.

Infection site: Gill lamellae.

Minimum prevalence: 7% (1 of 14 scamp infected).

Specimens studied: Holotype, USNM 1276207; 35 paratypes, USNM 1276208, NHMUK 2014.11.14.25–26, MNHN HEL488–491, FSBC-I 127751, 127752.

Etymology: The specific name (a noun) is from Latin (*contubernalis* = a companion or comrade) and refers to its co-occurrence with *P. vascellum* n. sp. on *M. phenax*.

#### Description (Figs. 97–105)

Body flattened dorsoventrally, with nearly parallel lateral margins along trunk. Tegument with numerous scales on peduncle; scales small, with rounded anterior margins. Cephalic region broad, with terminal and two bilateral poorly developed lobes, three bilateral pairs of head organs, three pairs of bilateral groups of cephalic-gland cells prepharyngeal (two pairs) and at level of pharynx. Four eyespots lacking lenses anterior to pharynx; members of posterior pair larger, closer together than those of anterior pair; accessory chromatic granules small, irregular in outline, infrequent in cephalic region. Pharynx subspherical, muscular; esophagus short to nonexistent; intestinal ceca blind, extending posteriorly to peduncle. Peduncle broad. Haptor with dorsal and ventral anteromedial lobes containing respective squamodiscs and lateral lobes having hook pairs 2–4, 6, 7. Squamodiscs subequal, with 12–14 (usually 13) concentric U-shaped rows of rodlets; ventral squamodisc with three innermost circular rows closed; dorsal squamodisc with two innermost circular rows closed. Ventral anchor with short superficial and deep roots; deep root with lateral swelling; shaft slightly arced, recurved point extending just short of level of tip of superficial root. Dorsal anchor with robust subtriangular base, short superficial root, knoblike deep root, arced shaft, and recurved point extending past level of tip of superficial root. Ventral bar with deep medial constriction, tapered ends, longitudinal ventral groove. Paired dorsal bar with subtriangular spatulate medial end. Hook with elongate slightly depressed thumb, delicate point, uniform shank; FH loop nearly shank length. Testis subspherical, postgermarial. Vas deferens originating from anterior margin of testis, extending anteriorly ventral to left intestinal cecum, forming seminal vesicle as a simple dilation; anterior duct of seminal vesicle having a circular loop before entering spherical thick-walled ejaculatory bulb; ejaculatory duct arising from bulb and extending to portal on proximal chamber of MCO; inconspicuous vesicle (prostatic reservoir?) with nearly clear contents lying to right of distal chamber of MCO. MCO reniform, usually quadriloculate, with short thin cone and elongate distal tube; distal two chambers of MCO with heavy thick walls; proximal two chambers with delicate walls, often collapsed (proximal-most chamber infrequently absent, Fig. 100); retractile filament variable in length. Germarium pyriform; germarial bulb pretesticular, with distal dorsoventral loop around right intestinal cecum; ootype slightly to left of body midline, surrounded by well-developed Mehlis’ gland, giving rise to delicate banana-shaped uterus when empty. Common genital pore ventral, dextral to cone of MCO. Vaginal pore sinistroventral at level of seminal vesicle; vaginal sclerite with distal funnel, distal tube (doubly recurved distally), and chamber having a tubular extension and one or more solid projections; proximal vaginal canal delicate, extending diagonally in trunk from vaginal sclerite to small subspherical seminal receptacle lying immediately anterior to Mehlis’ gland. Bilateral vitelline ducts not observed; vitellarium absent in regions of other reproductive organs, otherwise dense throughout trunk.

**Figures 97–105.**
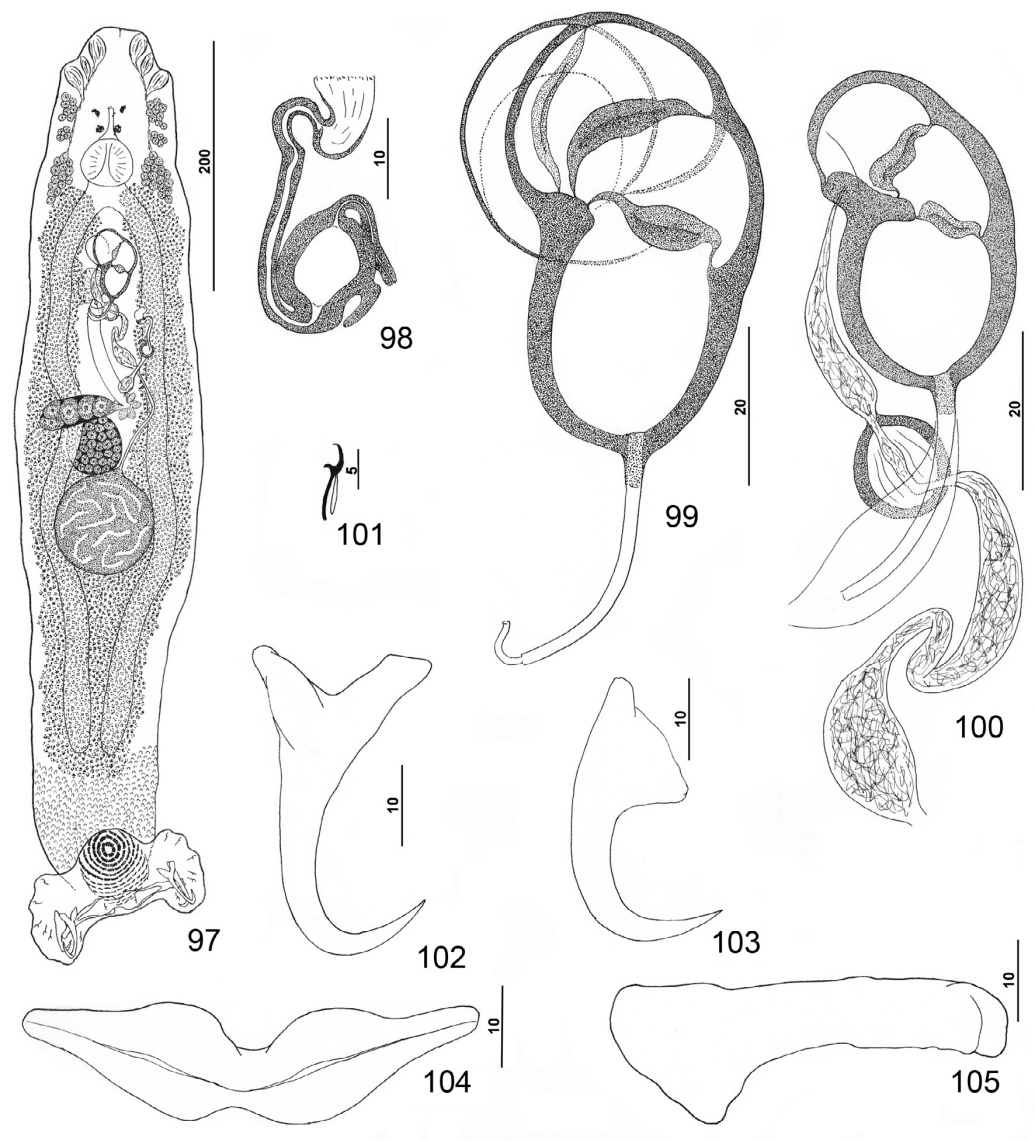
*Pseudorhabdosynochus contubernalis* n. sp. from scamp *Mycteroperca phenax*. 97: whole mount (composite, ventral view; dorsal squamodisc and dorsal anteromedial haptoral lobe not shown); 98: vaginal sclerite (dorsal view); 99: male copulatory organ (ventral view); 100: terminal male reproductive system showing MCO with collapsed or absent proximal chamber, seminal vesicle, distal vas deferens, and ejaculatory bulb and duct (ventral view); 101: hook; 102: ventral anchor; 103: dorsal anchor; 104: ventral bar; 105: left dorsal bar (ventral view).

Measurements: Body 707 (624–787; *n* = 21) long; width at level of germarium 133 (102–159; *n* = 23). Haptor 138 (120–154; *n* = 14) wide; squamodisc 53 (45–61; *n* = 18) long, 51 (40–61; *n* = 18) wide. Ventral anchor 40 (38–43; *n* = 13) long; dorsal anchor 35 (33–39; *n* = 13) long. Ventral bar 60 (54–67; *n* = 11) long; dorsal bar 53 (49–57; *n* = 12) long. Hook 11 (10–12; *n* = 18) long. Pharynx 45 (40–50; *n* = 24) wide. MCO 63 (56–68; *n* = 20) long (dimension does not include measurements of specimens for which the proximal chambers were lacking or had collapsed). Testis 79 (60–89; *n* = 16) long, 74 (49–88; *n* = 16) wide. Germarial bulb 48 (34–64; *n* = 21) wide.

#### Remarks

This species most closely resembles *P. vascellum* n. sp. It differs from *P. vascellum* by its larger overall size (body length about 700 μm vs. 500 μm) and length of the MCO (about 60 μm vs. 40 μm). In addition, the proximal two chambers of the MCO of *P. contubernalis* n. sp. have comparatively thin walls which frequently have collapsed or are absent (walls of proximal chambers sturdy and seldom collapsed in *P. vascellum*). *Pseudorhabdosynochus contubernalis* is a member of the group of *Pseudorhabdosynochus* species (*P. kritskyi*, *P. capurroi*, *P. mycteropercae* n. sp., *P. contubernalis*, *P. hyphessometochus* n. sp., and *P. vascellum*) infecting species assigned to *Mycteroperca* by having a prominent chamber and a distally recurved tube of the vaginal sclerite. It differs from all of these species by having a scaled tegument on the peduncle (tegumental scales absent in all other members of the group); in addition, it is differentiated from *P. kritskyi*, *P. capurroi*, *P. hyphessometochus*, and *P. vascellum* by having a doubly recurved distal tube of the vaginal sclerite (distal tube simply recurved in all of the latter species). The distal tube of the vaginal sclerite of *P. mycteropercae* is doubly recurved.

### *Pseudorhabdosynochus monaensis* Dyer, Williams & Bunkley-Williams, 1994

Type host and locality: Rock hind, *Epinephelus adscensionis* (Osbeck) (Serranidae: Epinephelinae: Epinephelini): Rock ridges off Playa Sardinera, Mona Island, Puerto Rico (18°04′ 12″ N, 67°57′ 36″ W).

Previous records: *Epinephelus adscensionis*: Rock ridges off Playa Sardinera, Mona Island, Puerto Rico (18°04′ 12″ N, 67°57′ 36″ W) [6].

Unconfirmed records: *Epinephelus adscensionis*: Desecheo Island, Puerto Rico [44]. *Epinephelus guttatus* (Serranidae: Epinephelinae: Epinephelini): Cabo Rojo, Puerto Rico [44].

*Infection site*: Gill lamellae.

Museum specimens examined: Holotype, USNPC 82789; two paratypes (one of which was misidentified as *P. monaensis* by Dyer et al. [6] and re-identified herein as *Pseudorhabdosynochus williamsi* n. sp.), USNPC 82790.

#### Redescription (Figs. 106–111)

Measurements [holotype and paratype USNPC 82790 (MT26-25C) measured]: Body 696–697 (*n* = 1) long; width at level of germarium 183–184 (*n* = 1). Haptor 159–160 (*n* = 1) wide. Pharynx 42 (40–45; *n* = 2). MCO 121 (118–125 (*n* = 2). Ventral anchor 37–38 (*n* = 1) long; dorsal anchor 39–40 (*n* = 1) long. Ventral bar 96–97 (*n* = 1) long; dorsal bar 58–59 (*n* = 1) long. Hook 11–12 (*n* = 1) long.

**Figures 106–111.**
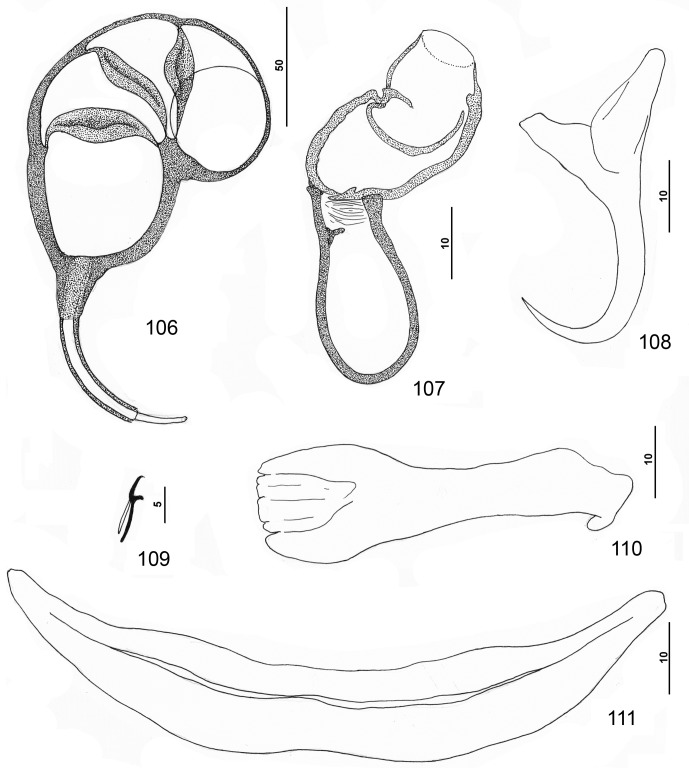
*Pseudorhabdosynochus monaensis* Dyer, Williams & Bunkley-Williams, 1994 from rock hind *Epinephelus adscensionis*. 106: male copulatory organ (dorsal view); 107: vaginal sclerite (dorsal view); 108: ventral anchor; 109: hook; 110: left dorsal bar (ventral view); 111: ventral bar.

#### Remarks

This species was not collected during the present study, and the three type specimens available in the USNPC were insufficient for a complete redescription of the species. Nonetheless, *P. monaensis* is easily distinguished from its congeners of the western Atlantic Ocean by its unique vaginal sclerite (Fig. 107). Other features defining the species include the elongate ventral bar with a minimal medial constriction (Fig. 111) and an MCO having a comparatively long cone (Fig. 106).

Dyer et al. [6] did not indicate how many specimens were used to develop the original description of this species but stated that six specimens were used to obtain measurements and that “specimens” had been deposited in the USNPC. USNPC records showed that the collection only holds three specimens of *P. monaensis* (the holotype and two paratypes). Whereas Dyer et al. [6] stated that the squamodiscs of *P. monaensis* had 20–24 rows of rodlets, only 14 or 15 rows were present in the squamodiscs of the holotype; 19 or 20 rows of rodlets were present in the squamodisc of the paratype [USNPC 82790 (MT26-25B)] collected from rock hind in Puerto Rico, which was here determined to represent a second species of *Pseudorhabdosynochus* on rock hind in Puerto Rico (see *P. williamsi* n. sp. below). The second paratype [USNPC 82790 (MT26-25C)], considered conspecific with the holotype based on comparative morphology of the vaginal sclerite, was damaged and lacked the haptor and squamodiscs. The finding of two species of *Pseudorhabdosynochus* within the type series of *P. monaensis* indicates the need for a redescription of the species based on fresh material from rock hind. Further, records of *P. monaensis* subsequent to the original description on rock hind in Puerto Rico [44] require confirmation.

### *Pseudorhabdosynochus mycteropercae* n. sp.

urn:lsid:zoobank.org:act:753506AC-A6C3-4D50-90CD-7E6E0554A3B7

Syn. *Diplectanum mycteropercae* of Mizelle & Wood (nomen nudum)

Type host and locality: Tiger grouper, *Mycteroperca tigris* (Valenciennes) (Serranidae: Epinephelinae: Epinephelini): 8 mi NW of St. George’s Island, Bermuda.

Current record: *Mycteroperca tigris* (FSBC 11990): ~15 km NNW of Dry Tortugas National Park, Florida (24.533° N, 82.983° W; these coordinates are in the collecting records of the FSBC collection but are not congruent with the written collecting notes, the latter of which are considered to be more accurate in this case), April 23, 1980.

Infection site: Not recorded for type specimens by Mizelle & Wood (unpublished) (probably gill lamellae); gill lamellae (voucher specimens).

Minimum prevalence: 100% (1 specimen from Florida examined and infected).

Specimens studied: Holotype, USNPC 72750; 2 paratypes, USNPC 72751; 23 voucher specimens, USNM 1273683, NHMUK 2015.2.25.5–6, MNHN HEL536–537, FSBC-I 127761, 127762.

Etymology: *Pseudorhabdosynochus mycteropercae* is named after the host genus, *Mycteroperca* Gill, species of which serve as hosts for the morphologically similar group of *Pseudorhabdosynochus* spp. (*P. kritskyi*, *P. capurroi*, *P. vascellum*, *P. contubernalis, P. hyphessometochus*, and *P. mycteropercae*).

#### Description (Figs. 112–119)

Body flattened dorsoventrally, elongate ovate in dorsoventral view. Tegument smooth. Cephalic region broad, with terminal and two bilateral poorly developed lobes; head organs, cephalic-gland cells not observed. Four eyespots anterior to pharynx lacking lenses; members of posterior pair larger, closer together than those of anterior pair; chromatic granules small, irregular in outline; accessory granules infrequent or absent in cephalic region. Pharynx subspherical; gut not observed. Peduncle broad. Squamodiscs subequal, with 11–13 concentric rows of rodlets; ventral squamodisc with two or three innermost rows closed; dorsal squamodisc with two innermost rows closed. Shape of haptor indistinct. Ventral anchor with short superficial and deep roots; deep root with lateral swelling; shaft slightly arced, recurved point extending to level of tip of superficial root. Dorsal anchor with subtriangular base, inconspicuous superficial root, knoblike deep root, minimally arced shaft, and slightly recurved point extending past level of tip of superficial root. Ventral bar with conspicuous medial constriction, tapered ends, longitudinal ventral groove. Paired dorsal bar with spatulate medial end. Hook with elongate slightly depressed thumb, delicate point, uniform shank; FH loop nearly shank length. Gonads indistinct; soft reproductive ducts not observed. MCO reniform, quadriloculate, with short cone and elongate distal tube (based on voucher specimens); retractile filament not observed. Vaginal sclerite variable, with distal funnel, doubly recurved tube, large spherical to ovate chamber having open or nipplelike anterior wall; proximal vaginal canal, seminal receptacle not observed. Vitellarium absent in regions of other reproductive organs, otherwise dense throughout trunk.

**Figures 112–119.**
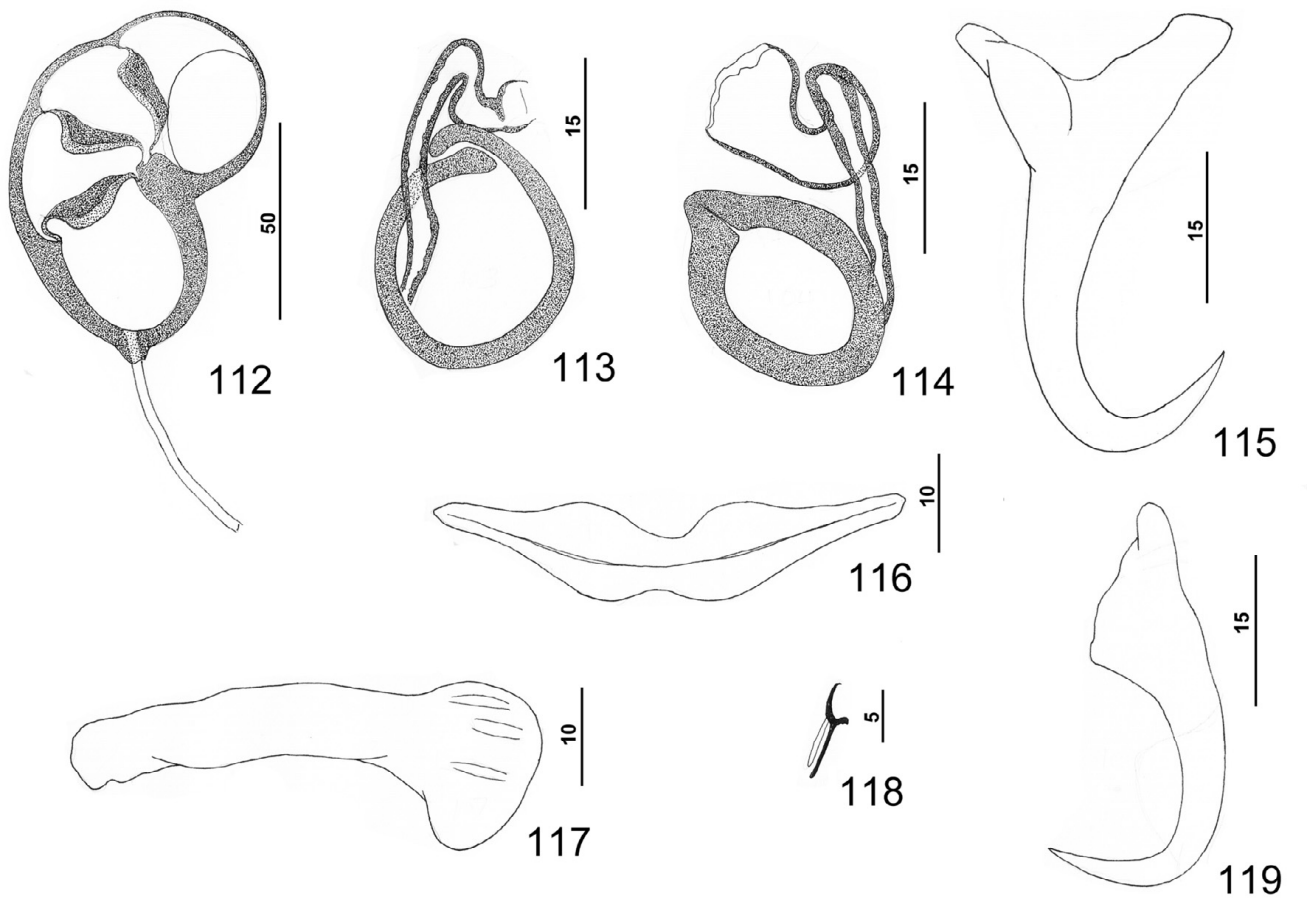
*Pseudorhabdosynochus mycteropercae* n. sp. from tiger grouper *Mycteroperca tigris*. 112: male copulatory organ (dorsal view, voucher specimen); 113: vaginal sclerite (dorsal view); 114: vaginal sclerite (ventral view, voucher specimen); 115: ventral anchor (voucher specimen); 116: ventral bar; 117: right dorsal bar (ventral view); 118: hook; 119: dorsal anchor. All drawings are from the type specimens unless indicated otherwise.

Measurements (dimensions of the voucher specimens from Florida follow in brackets those of the types): Body 670 (596–765; *n* = 3) long; width at level of germarium 165 (150–175; *n* = 3). Haptor 144 (133–150; *n* = 3) wide; squamodisc 52 (45–59; *n* = 2) [62 (55–70; *n* = 7)] long, 55 (47–63; *n* = 2) [55 (48–58; *n* = 6)] wide. Ventral anchor 41 (40–42; *n* = 2) [46 (44–50; *n* = 12)] long; dorsal anchor 38 (36–40; *n* = 2) [42 (39–44; *n* = 13)] long. Ventral bar 57 (50–63; *n* = 3) [63 (60–67; *n* = 10)] long; dorsal bar 54 (47–60; *n* = 3) [57 (53–60; *n* = 13)] long. Hook 11–12 (*n* = 6) [11–12 (*n* = 13)] long. MCO 89 (86–92; *n* = 8) long.

#### Remarks

The description of *P. mycteropercae* n. sp. is based on three type specimens collected from the tiger grouper in Bermuda by John D. Mizelle and Raymond M. Wood, who deposited them in the USNPC as *Diplectanum mycteropercae* (nomen nudum), and supplemented by 23 voucher specimens from a tiger grouper in the ichthyology collection of the Florida Fish and Wildlife Conservation Commission in St. Petersburg, Florida (FSBC 11990). Although the type and voucher specimens were of sufficient quality to determine that they represented the new species, many features of the reproductive systems could not be discerned and a drawing of a whole mount was not possible.

The species differs from other members of the group of species infecting *Mycteroperca* spp. by having an open chamber of the vaginal sclerite (chamber of *P. kritskyi*, *P. capurroi*, *P. vascellum* n. sp., and *P. contubernalis* n. sp. is closed; in *P. hyphessometochus* n. sp., the closed anterior wall of the chamber is formed by the two overlapping ends of the chamber wall). In addition, the cavity of the chamber in *P. vascellum* is small (large in *P. mycteropercae*); the wall of the chamber in *P. contubernalis* has external projections (absent in *P. mycteropercae*); the distal portion of the distal tube of the vaginal sclerite is simply recurved in *P. kritskyi*, *P. capurroi*, and *P. hyphessometochus* (distal tube doubly recurved in *P. mycteropercae*). Other differences include a comparatively small spatulate medial end of the dorsal bars in *P. mycteropercae* (medial ends large in *P. kritskyi* and *P. capurroi* and subtriangular in *P. contubernalis*) and a ventral anchor having a deep root shorter than the superficial root in *P. mycteropercae* (deep roots subequal or longer than the superficial roots in *P. kritskyi*, *P. capurroi*, *P. hyphessometochus*, and *P. vascellum*).

### *Pseudorhabdosynochus williamsi* n. sp.

urn:lsid:zoobank.org:act:5CB5AB39-A806-4682-978E-B3CB6454B245

Syn. *Pseudorhabdosynochus monaensis* Dyer, Williams & Bunkley-Williams, 1994 (pro parte).

Type host and locality: Rock hind, *Epinephelus adscensionis* (Osbeck) (Serranidae: Epinephelinae: Epinephelini) (FSBC 4215): ~40 mi offshore from St. Andrews Bay, Panama City, Florida (heading 205°) (coordinates not available), June 19, 1965.

Other record: *Epinephelus adscensionis* (FSBC 1808): ~70 mi W of St. Petersburg, Florida (27.781° N, 83.941° W), July 11, 1960.

Previous record: *Epinephelus adscensionis*: Rock ridges off Playa Sardinera, Mona Island, Puerto Rico (18°04′ 12″ N, 67°57′ 36″ W) as *P. monaensis* (pro parte) [6].

Infection site: Gill lamellae.

Minimum prevalence: 100% (2 of 2 rock hind examined and infected).

Specimens studied: Holotype, USNM 1276209; 39 paratypes, USNM 1276210, NHMUK 2014.11.14.27–28, MNHN HEL492–495, FSBC-I 127759, 127760; 2 vouchers, USNM 1276211.

Museum specimen examined: Paratype of *P. monaensis*, USNPC 82790 (MT26-25B) (redetermined as *P. williamsi*).

Etymology: This species is named for Dr. Ernest (Bert) H. Williams, Jr., professor (retired), University of Puerto Rico, Lajas, Puerto Rico, in recognition of his extensive research on the parasites of fishes in the Caribbean region. Dr. Williams was one of the investigators who collected the voucher specimen (a paratype of *P. monaensis*) of this species from rock hind in Puerto Rico.

#### Description (Figs. 120–128)

Body flattened dorsoventrally, subovate in dorsoventral view. Tegumental scales scattered on peduncle. Cephalic region broad, with terminal and two bilateral poorly developed lobes; three pairs of head organs; pair of bilateral groups of cephalic-gland cells lateral to pharynx. Four eyespots anterior to pharynx lacking lenses; members of posterior pair larger, closer together than those of anterior pair; chromatic granules small, irregular in outline; few accessory granules near eyespots or absent in cephalic region. Pharynx subspherical; esophagus short to nonexistent; intestinal ceca blind, extending to level of anterior limit of peduncle. Peduncle broad. Haptor with dorsal and ventral anteromedial lobes containing respective squamodiscs and lateral lobes having hook pairs 2–4, 6, 7. Squamodiscs subequal, with 19 open concentric U-shaped rows of delicate rodlets. Ventral anchor with short superficial and deep roots; deep root with lateral swelling; elongate shaft slightly arced; recurved point extending to just past level of tip of superficial root. Dorsal anchor with narrow base, short roots, elongate straight shaft, recurved point extending past level of tip of superficial root. Ventral bar elongate, with medial constriction, tapered ends, longitudinal ventral groove. Paired dorsal bar with slightly expanded medial end. Hook with depressed thumb, delicate point, uniform shank; FH loop nearly shank length. Testis subspherical; vas deferens, seminal vesicle, dextral vesicle (prostatic reservoir?) not observed; ejaculatory bulb small, ejaculatory duct entering dorsal portal of MCO. MCO reniform, quadriloculate, with comparatively long delicate cone; walls of chambers variably thick (Figs. 121 and 122); distal tube short; retractile filament not observed. Germarium pyriform, dorsoventrally looping around right intestinal cecum; Mehlis’ gland indistinct; uterus with delicate walls, often collapsed. Vaginal sclerite small, with short distal tube and small chamber; proximal vaginal canal and seminal receptacle not observed. Vitellarium absent in regions of other reproductive organs, otherwise dense throughout trunk.

**Figures 120–128.**
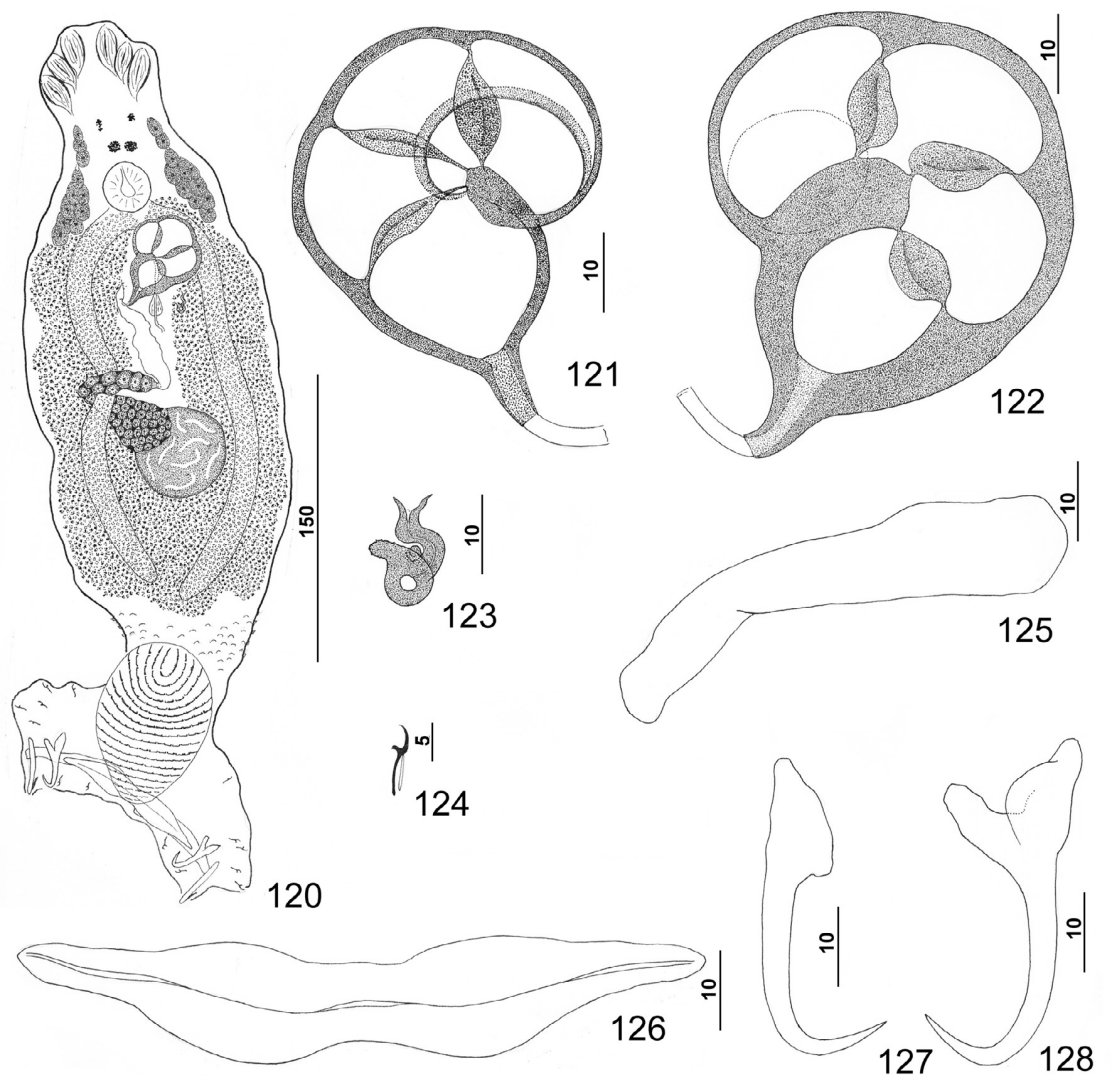
*Pseudorhabdosynochus williamsi* n. sp. from rock hind *Epinephelus adscensionis*. 120: whole mount (composite, ventral view; dorsal squamodisc and dorsal anteromedial haptoral lobe not shown); 121: male copulatory organ (dorsal view) with thin chamber walls; 122: male copulatory organ (ventral view) with thick walls of the chambers; 123: vaginal sclerite (ventral view); 124: hook; 125: right dorsal bar (ventral view); 126: ventral bar; 127: dorsal anchor; 128: ventral anchor.

Measurements: Body 495 (349–655; *n* = 11) long; width at level of germarium 127 (109–161; *n* = 11). Haptor 153 (127–183; *n* = 7) wide; squamodisc 82 (70–100; *n* = 16) long, 56 (48–71; *n* = 14) wide. Ventral anchor 34 (31–37; *n* = 14) long; dorsal anchor 37 (35–40; *n* = 13) long. Ventral bar 76 (67–85; *n* = 13) long; dorsal bar 50 (41–61; *n* = 15) long. Hook 11 (10–12; *n* = 18) long. Pharynx 28 (23–33; *n* = 20) wide. MCO 51 (40–62; *n* = 19) long. Testis 48 (41–53; *n* = 7) long, 52 (40–64; *n* = 7) wide. Germarial bulb 32 (27–36; *n* = 7) wide.

#### Remarks

This species was first thought to be distinct upon examination of the type specimens of *P. monaensis* deposited in the USNPC, when one of the specimens [USNPC 82790 (MT26-25B)] was found to lack the distinctive vaginal sclerite of *P. monaensis*. Confirmation of the validity of the specimen representing an undescribed species was obtained when numerous specimens of the species were found infecting two rock hinds off Florida. In that species, herein named *P. williamsi* n. sp., the vaginal sclerite is comparatively small with a small subspherical chamber [vaginal sclerite much larger and with an ovate chamber in *P. monaensis* (compare Figs. 107 and 123)]. *Pseudorhabdosynochus williamsi* differs further from *P. monaensis* by possessing a small MCO (about 51 μm in *P. williamsi* vs. 121 μm in *P. monaensis*), and dorsal bars with a minimally expanded medial end (medial end of dorsal bar about twice as wide as the proximal end in *P. monaensis*). The new species most closely resembles *P. justinella* n. sp. and *P. meganmarieae* n. sp. based on the comparative morphology of the respective vaginal sclerites. It differs from *P. justinella* by having a delicate cone of the MCO (cone robust in *P. justinella*) and a ventral bar with a moderately constricted medial region (constriction minimal in *P. justinella*). It differs from *P. meganmarieae* by the minimally expanded medial end of the dorsal bars (medial end of dorsal bar about twice as wide as the distal end in *P. meganmarieae*) and by having the shaft of the ventral anchor forming a gentle arc (shaft comparatively straight in *P. meganmarieae*). Finally, the morphology of the MCO varied significantly among specimens of *P. williamsi*. In what appeared to be fully mature specimens, the walls of the respective chambers were noticeably thicker than those of putatively younger specimens (compare Figs. 121 and 122).

*Pseudorhabdosynochus bocquetae* (Oliver & Paperna, 1984) Kritsky & Beverley-Burton, 1986 was described from a fish identified as *Epinephelus adscensionis* and collected from the Gulf of Aqaba [40]. This species possesses a relatively non-descript vaginal sclerite similar to that of *P. williamsi*. *Pseudorhabdosynochus williamsi*, however, is easily distinguished from *P. bocquetae* by having 19 open concentric rows of rodlets in the squamodisc (8–10 rows with the innermost three rows closed in *P. bocquetae*).

The recording of *P. bocquetae* on *E. adscensionis* in the Gulf of Aqaba is clearly erroneous. The fish host reported by Oliver & Paperna [40] for *P. bocquetae* apparently does not occur within these waters. According to Heemstra & Randall [16], rock hind are known from Ascension and St. Helena islands and the western Atlantic from Massachusetts into the Gulf of Mexico and Caribbean Sea, and south to southern Brazil. It is highly unlikely, therefore, that Oliver & Paperna [40] had rock hind in their collections.

### *Pseudorhabdosynochus mizellei* n. sp.

urn:lsid:zoobank.org:act:85100365-3F1E-4DDD-8BCC-3761CE750718

Syns *Diplectanum epinepheli* of Mizelle & Wood (nomen nudum), *nec* Yamaguti [55] (pro parte); *Diplectanum* sp. in USNPC Specimen Log (pro parte).

Type host and locality: Red hind, *Epinephelus guttatus* (Linnaeus) (Serranidae: Epinephelinae: Epinephelini) (FSBC 7520): Florida Middle Grounds, Gulf of Mexico (28.453° N, 84.217° W), January 23, 1972.

Other records: *Epinephelus guttatus*: Open Gulf of Mexico, ~17 km SSW of Loggerhead Key, Florida (24.483° N, 82.967° W), March 19, 1980 (FSBC 12123); ~70 mi W of Key West, Florida (24.483° N, 82.967° W), March 29, 2008 (FSBC 11284); offshore, Marathon, Florida (coordinates not available), May 3, 1962 (FSBC 2160); off Castle Roads, Bermuda, 1973 (as *Diplectanum epinepheli* of Mizelle & Wood [nomen nudum], nec Yamaguti [55]).

Infection site: Gill lamellae.

Minimum prevalence: 100% (4 of 4 red hind from Florida examined and infected).

Specimens studied: Holotype, USNM 1276218; 25 paratypes, USNM 1276219–1276222, NHMUK 2014.11.14.33–34, MNHN HEL502–503, FSBC-I 127749, 127750.

Museum specimens examined: Holotype (*Diplectanum epinepheli* of Mizelle & Wood, unpublished, nec Yamaguti [55]), USNPC 72746; paratype (*Diplectanum epinepheli* of Mizelle & Wood, unpublished, nec Yamaguti [55], USNPC 72747 (M1421-4). These specimens are herein considered voucher specimens of *P. mizellei*.

Etymology: This species is named for the late Dr. John D. Mizelle, mentor of the senior author and codiscoverer of this new species in Bermuda, in recognition of his extensive research on monogenoids occurring in North and South America.

#### Description (Figs. 129–136)

Body flattened dorsoventrally, elongate-ovate in dorsoventral view. Tegument with numerous small scales in posterior trunk and peduncle; scales indistinct or appearing absent in poorly fixed specimens. Cephalic region narrow when body relaxed, with terminal and two bilateral poorly to moderately developed lobes; head organs large, usually poorly defined; pair of bilateral groups of cephalic-gland cells lateral or posterolateral to pharynx. Four eyespots prepharyngeal, equidistant, lacking lenses; members of posterior pair larger than those of anterior pair; chromatic granules small, irregular in outline; accessory granules absent. Pharynx subspherical; esophagus short; intestinal ceca blind, extending to level of anterior limit of peduncle. Peduncle broad, tapered posteriorly. Haptor with dorsal and ventral anteromedial lobes containing respective squamodiscs and lateral lobes having hook pairs 2–4, 6, 7. Squamodiscs subequal; each with 11–12 (usually 11) concentric U-shaped rows of rodlets; innermost row closed, subcircular. Ventral anchor with deep root shorter than superficial root, slightly curved shaft, point extending to level of tip of superficial root. Dorsal anchor with subtriangular base, short upright superficial root, knoblike deep root, arced shaft, and recurved point extending past level of tip of superficial root. Ventral bar with slight medial constriction, tapered ends, longitudinal ventral groove. Paired dorsal bar with slightly expanded medial end. Hook with slightly depressed thumb, delicate point, uniform shank; FH loop approaching shank length. Testis subspherical; distal soft tissue organs of male reproductive system not observed. MCO reniform, quadriloculate, with elongate cone, short distal tube, variable retractile filament. Germarium pyriform; seminal receptacle, Mehlis’ gland not observed. Vaginal sclerite comprising two tandem thick-walled chambers having small cavities; vaginal canal not observed. Vitellarium absent in regions of other reproductive organs, otherwise dense throughout trunk and anterior portions of peduncle.

**Figures 129–136.**
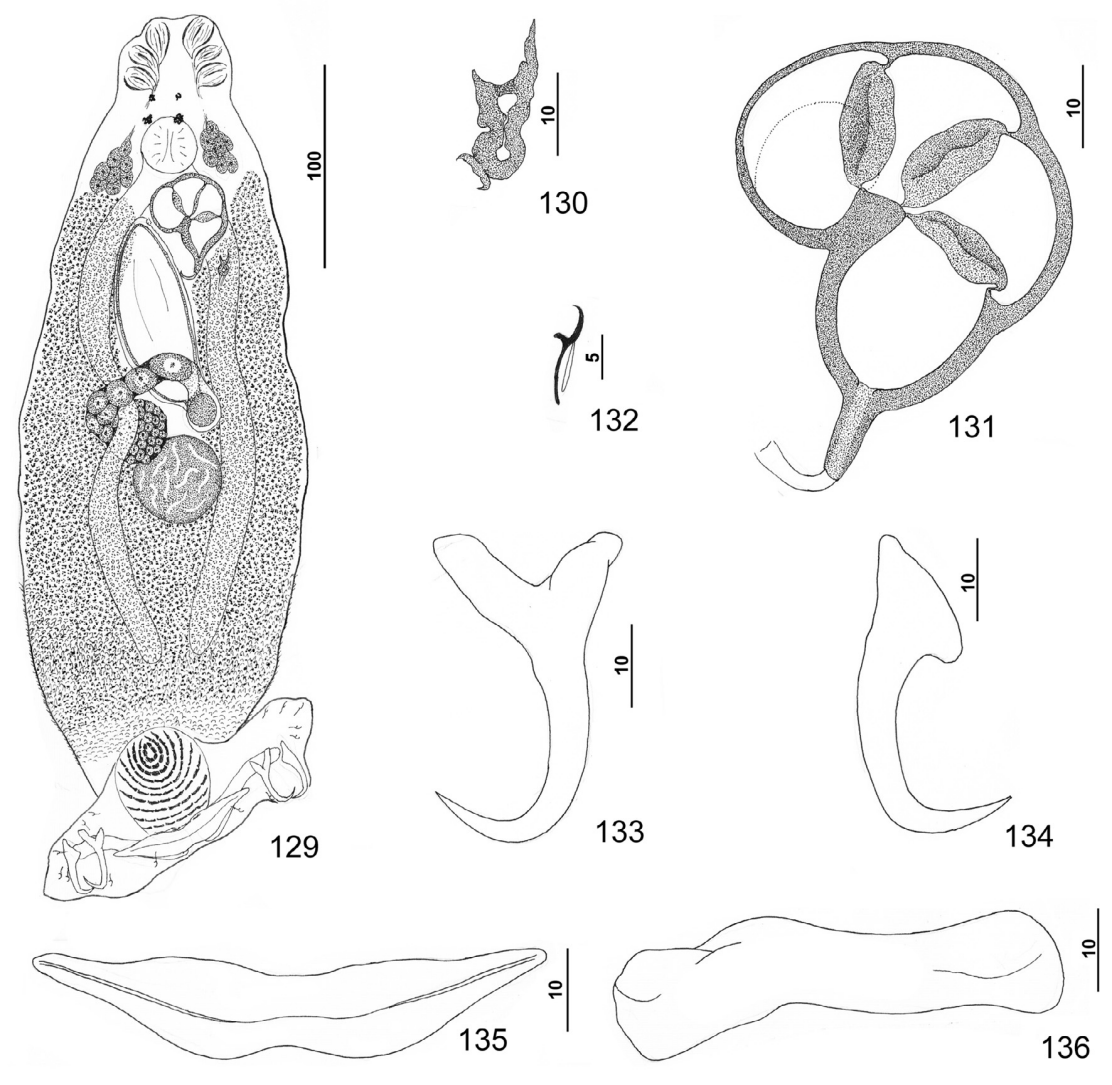
*Pseudorhabdosynochus mizellei* n. sp. from red hind *Epinephelus guttatus*. 129: whole mount (composite, ventral view; dorsal squamodisc and dorsal anteromedial haptoral lobe not shown); 130: vaginal sclerite (ventral view); 131: male copulatory organ (ventral view); 132: hook; 133: ventral anchor; 134: dorsal anchor; 135: ventral bar; 136: left dorsal bar (ventral view).

Measurements (dimensions of Mizelle and Wood’s specimens follow in brackets those of Florida specimens): Body 384 (316–437; *n* = 4) [384 (329–438; *n* = 2)] long; width at level of germarium 151 (133–178; *n* = 4) [132 (115–150; *n* = 2)]. Haptor 147 (138–157; *n* = 4) [137 (131–143; *n* = 2)] wide; squamodisc 47 (39–56; *n* = 22) [45 (44–47; *n* = 2)] long, 49 (44–54; *n* = 22) [51 (48–53; *n* = 2)] wide. Ventral anchor 36 (33–40; *n* = 19) long; dorsal anchor 34 (31–38; *n* = 22) [35 (33–36; *n* = 2)] long. Ventral bar 63 (46–72; *n* = 20) [63 (57–70; *n* = 2)] long; dorsal bar 52 (46–57; *n* = 22) [53 (50–55; *n* = 2)] long. Hook 12 (10–13; *n* = 19) [12 (11–13; *n* = 2)] long. Pharynx 32 (31–33; *n* = 4) wide. MCO 49 (47–51; *n* = 4) long. Testis 42 (29–53; *n* = 4) long, 46 (35–59; *n* = 4) wide. Germarial bulb 30 (27–36; *n* = 4) wide.

#### Remarks

*Pseudorhabdosynochus mizellei* n. sp. most closely resembles *P. williamsi* n. sp. in the basic morphology of the vaginal sclerite and MCO. In both species, the sclerite has small chambers and the cone of the MCO is elongate. The new species is easily differentiated from *P. williamsi* by the deep root of the ventral anchor being shorter than the superficial root and the shaft of the dorsal anchor forming a gentle curve (dorsal-anchor shaft comparatively straight in *P. williamsi*). In addition, the ventral and dorsal bars of *P. mizellei* are noticeably shorter than those of *P. williamsi*.

Although *P. mizellei* occurs concurrently with *P. woodi* n. sp. on red hind in Bermuda (based on specimens collected by Mizelle and Wood, unpublished, and deposited in the USNM), the latter species was not recovered from red hind collected off Florida during the present study. *Pseudorhabdosynochus mizellei* differs from *P. woodi* by having a comparatively small vaginal sclerite with two small chambers. The vaginal sclerite of *P. woodi* has a larger single ovate chamber (compare Figs. 130 and 156).

### *Pseudorhabdosynochus beverleyburtonae* (Oliver, 1984) Kritsky & Beverley-Burton, 1986

Syns *Diplectanum americanum* of Euzet & Oliver (1965), nec Price [42]; *Cycloplectanum americanum* (Price, 1937) Oliver, 1968 (pro parte); *Cycloplectanum beverleyburtonae* Oliver, 1984.

Type host and locality: Dusky grouper, *Epinephelus guaza* (Linnaeus) [now *Mycteroperca marginata* (Lowe)] (Serranidae: Epinephelinae: Epinephelini): Côte Vermeille (Golfe du Lion, Méditerranée occidentale), France.

Current records: *Mycteroperca marginata*: Atlantic Ocean off Barra Velha, State of Santa Catarina, Brazil (groupers purchased from local fishermen at the Barra Velha fish market; hosts apparently caught the previous nights of December 18, 2013, February 3, 2014).

Previous records: *Epinephelus guaza* (now *M. marginata*): Côte Vermeille (Golfe du Lion, Méditerranée occidentale), France (as *Cycloplectanum beverleyburtonae*) [38]; Mer Catalane, Rosas, France (as *C. beverleyburtonae*) [39]; Bay of Naples, Italy (as *C. americanum*) [49]; Banyuls sur Mer, Mediterranean Sea, France (as *C. americanum*) [36, 37]. *E. gigas* (Brünnich) (an ambiguous synonym of *M. marginata*): Banyuls sur Mer, Mediterranean Sea, France (as *Diplectanum americanum*) [10]. *Epinephelus marginatus* (now *M. marginata*): off Ilhas Cagarras, Rio de Janeiro, Brazil (23°02′ S, 43°12′ W) [46]; Ubatuba, coast of São Paulo, southeastern Brazil (23°26′ 20″ S, 45°01′ 37″ W) [45] (both as *Pseudorhabdosynochus beverleyburtonae*).

Infection site: Gill lamellae.

Minimum prevalence: 100% (4 of 4 dusky grouper from Brazil infected).

Specimens studied: 52 voucher specimens, USNM 1276185, 1276186, NHMUK 2014.11.14.11–15, MNHN HEL462–472, FSBC-I 127730–127734.

Museum specimens examined: 2 syntypes, MNHN 249H-TC 167, 249H-TC 167 bis; paratype, USNPC 77469; voucher specimen, NHMUK 1999.1.6.4–6; the specimen from the USNPC is apparently mislabeled as a paratype because Oliver [38] did not designate a holotype but considered all specimens on which the description was based as syntypes.

#### Redescription (Figs. 137–144)

Body dorsoventrally flattened, with nearly parallel lateral margins. Tegument smooth, lacking scales. Cephalic region broad, with terminal and two bilateral poorly developed lobes, three bilateral pairs of head organs, pair of bilateral groups of cephalic-gland cells at level of pharynx. Two pairs of prepharyngeal eyespots lacking lenses; members of posterior pair larger, closer together than those of anterior pair; chromatic granules small, irregular in outline; accessory granules generally absent in cephalic region. Pharynx subspherical to subovate, muscular; esophagus short; intestinal ceca blind, extending posteriorly to level of anterior limit of peduncle. Peduncle broad, tapered posteriorly. Haptor with dorsal and ventral anteromedial lobes containing respective squamodiscs and lateral lobes having hook pairs 2–4, 6, 7. Squamodiscs subequal, with 10–12 (usually 12) concentric U-shaped rows of rodlets; innermost two or three rows closed, circular. Ventral anchor with short superficial and deep roots; deep root with small lateral swelling; shaft curved; short recurved point extending to level of tip of superficial root. Dorsal anchor with subtriangular base, superficial root short to lacking, deep root knoblike, arcing shaft, short recurved point extending slightly past level of tip of superficial root. Ventral bar with conspicuous medial constriction, tapered ends, longitudinal medioventral groove. Paired dorsal bar with spatulate irregular medial end, bilobed lateral end. Hook with depressed thumb, delicate point, uniform shank; FH loop nearly shank length. Testis subspherical, lying sinistroposterior to germarium along body midline; proximal vas deferens not observed; seminal vesicle a simple dilation of vas deferens; distal vas deferens forming a sigmoid curve before entering lacriform ejaculatory bulb; ejaculatory duct entering dorsal portal of proximal chamber of MCO; vesicle with reticulate contents (prostatic reservoir?) dextral to MCO. MCO quadriloculate, with thick walls, long tapered cone, elongate distal tube, and long protruding filament. Germarium pyriform; germarial bulb lying to right of body midline and slightly overlapping testis, with distal loop dorsoventrally around right intestinal cecum; ootype lying on body midline, with poorly developed Mehlis’ gland; uterus delicate, banana shaped when empty. Common genital pore ventral, dextral to distal chamber of MCO. Vaginal pore sinistroventral posterior to MCO at level of seminal vesicle; vaginal vestibule delicate; vaginal sclerite with distal sigmoid tube giving rise to elongate U-shaped chamber with slight dilation before narrowing and then opening into small subspherical chamber; vaginal canal delicate, leading to seminal receptacle. Seminal receptacle subspherical, on body midline ventral to proximal end of uterus. Bilateral vitelline ducts not observed; vitellarium absent in regions of other reproductive organs, otherwise dense throughout trunk. Egg elongate-ovate, lacking filaments.

**Figures 137–144.**
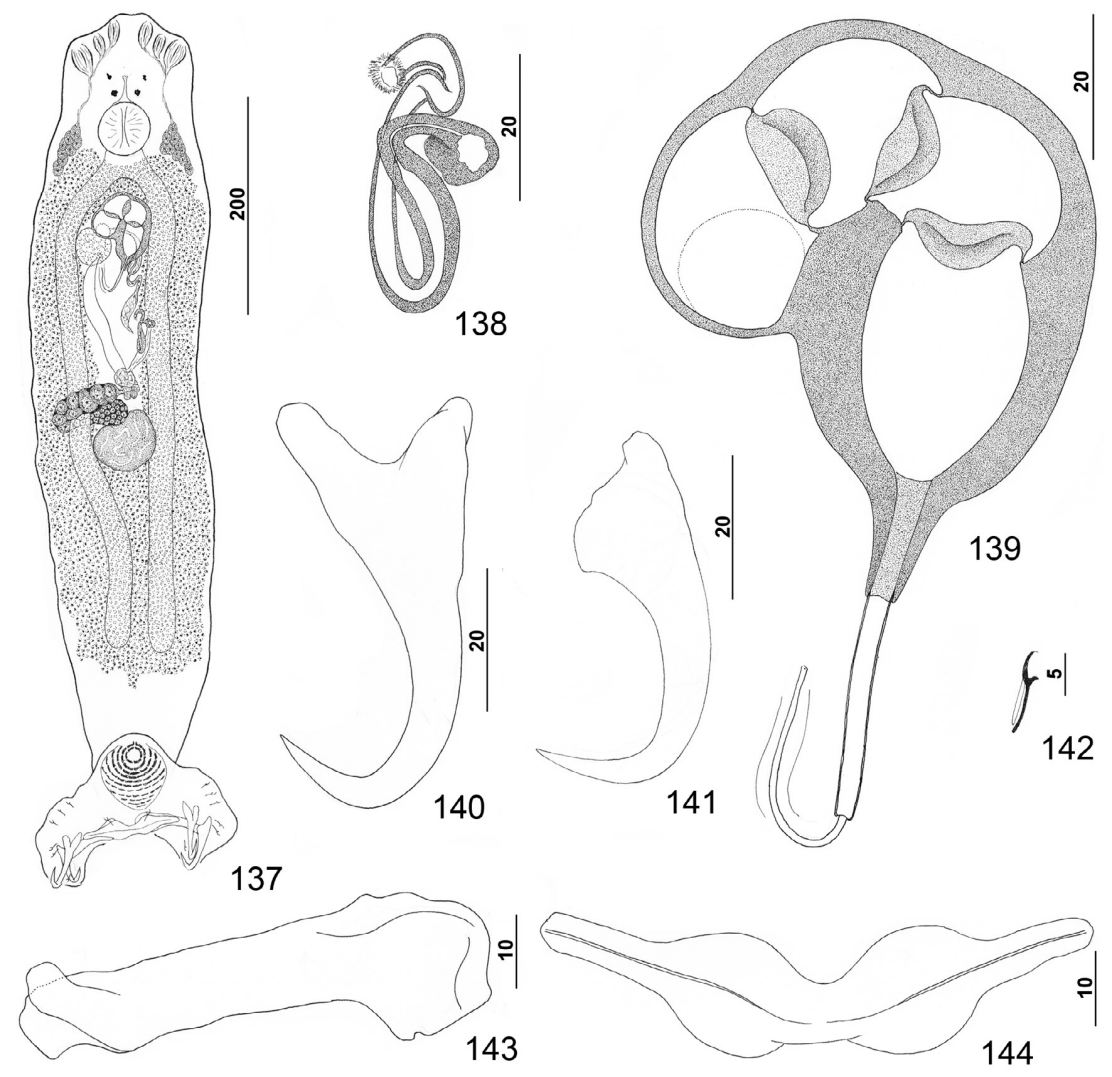
*Pseudorhabdosynochus beverleyburtonae* (Oliver, 1984) Kritsky & Beverley-Burton, 1986 from dusky grouper *Mycteroperca marginata*. 137: whole mount (composite, ventral view; dorsal squamodisc and dorsal anteromedial haptoral lobe not shown); 138: vaginal sclerite (ventral view); 139: male copulatory organ (ventral view); 140: ventral anchor; 141: dorsal anchor; 142: hook; 143: left dorsal bar (dorsal view); 144: ventral bar.

Measurements: Body 741 (569–974; *n* = 24) long; width at level of germarium 170 (143–235; *n* = 27). Haptor 168 (141–197; *n* = 22) wide; squamodisc 56 (47–64; *n* = 32) long, 55 (42–67; *n* = 34) wide. Ventral anchor 56 (50–61; *n* = 15) long; dorsal anchor 49 (44–53; *n* = 16) long. Ventral bar 77 (72–84; *n* = 13) long; dorsal bar 67 (61–72; *n* = 15) long. Hook 12 (11–13; *n* = 10) long. Pharynx 47 (39–60; *n* = 31) wide. MCO 84 (75–100; *n* = 28) long. Testis 69 (46–93; *n* = 20) long, 70 (54–95; *n* = 20) wide. Germarial bulb 47 (39–65; *n* = 17) wide. Egg 87–88 (*n* = 1) long, 35–36 (*n* = 1) wide.

#### Remarks

This species was first recorded by Euzet & Oliver [10] as *Diplectanum americanum* from dusky grouper collected from the Mediterranean Sea and later by Ulmer & James [49] and Oliver [36, 37] as *Cycloplectanum americanum* from the Mediterranean Sea. That the helminth from dusky grouper in the Mediterranean Sea represented a species distinct from *C. americanum* (now *P. americanum*) was later recognized by Oliver [38], who described it as *Cycloplectanum beverleyburtonae*. Kritsky & Beverley-Burton [25] transferred the species to *Pseudorhabdosynochus* when it was determined that *C. americanum* (sensu Price [42]), type species of *Cycloplectanum* Oliver, 1968, was a member of the senior *Pseudorhabdosynochus* Yamaguti, 1958. Although the synonymy of the two genera is broadly accepted, Oliver [39] continued to consider them distinct and *C. beverleyburtonae* as the valid name of this helminth.

The dusky grouper is one of only four epinephelin groupers with a trans-Atlantic distribution. In addition to *M. marginata*, the trans-Atlantic species include the rock hind, *E. adscensionis*, the Atlantic goliath grouper, *E. itajara*, and the Atlantic creolefish, *Paranthias furcifer* (Valenciennes). Dusky groupers are known from western Europe and the Mediterranean Sea to south around the southern tip of Africa; in the Americas, they have been reported from the coastal areas of southern Brazil [16]. It is the only trans-Atlantic grouper known to harbor a species of *Pseudorhabdosynochus* (*P. beverleyburtonae*) throughout its geographic range. Comparison of the type specimens of *P. beverleyburtonae* (USNPC 77469; MNHN 249H-TC 167, 249H-TC 167 bis) with the voucher specimen of Santos et al. [46] (NHMUK 1999.1.6.4–6) and those of the present study from *M. marginata* off southern Brazil, did not reveal any morphological features that distinguished eastern Atlantic from western Atlantic specimens. Thus, the Mediterranean and western Atlantic populations are considered conspecific as first proposed by Santos et al. [46].

### *Pseudorhabdosynochus bunkleywilliamsae* n. sp.

urn:lsid:zoobank.org:act:4FFF5DBF-BCE6-41C9-B22A-0A2DB3045718

Type host and locality: Nassau grouper, *Epinephelus striatus* (Bloch) (Serranidae: Epinephelinae: Epinephelini): La Parguera, Puerto Rico (17°58′ N, 67°13′ W), October 29, 1992.

Infection site: Gill lamellae.

Minimum prevalence: 100% (1 of 1 Nassau grouper from Puerto Rico examined and infected).

Specimens studied: Holotype, USNM 1251933; 46 paratypes, USNM 1251934, NHMUK 2014.11.14.16–20, MNHN HEL473–480, FSBC-I 127722–127725.

Etymology: This species is named for Dr. Lucy Bunkley-Williams, University of Puerto Rico, Mayagüez, Puerto Rico, in recognition of her extensive research on the parasites of fishes occurring within the environs of Puerto Rico. She and Dr. Ernest Williams collected and preserved the specimens of *P. bunkleywilliamsae* n. sp. on which the present description is based.

#### Description (Figs. 145–153)

Body flattened dorsoventrally, elongate ovate in dorsoventral view. Tegument smooth. Cephalic region broad, with terminal and two bilateral poorly developed lobes, three bilateral pairs of head organs, pairs of bilateral groups of cephalic-gland cells prepharyngeal and at level of pharynx. Four eyespots lacking lenses anterior to pharynx; members of posterior pair larger, equidistant, or slightly closer together than those of anterior pair; chromatic granules small, subovate; accessory granules absent in cephalic region. Pharynx subspherical, muscular; esophagus short; intestinal ceca blind, extending posteriorly to anterior limit of peduncle. Peduncle broad. Haptor with dorsal and ventral anteromedial lobes containing respective squamodiscs and lateral lobes having hook pairs 2–4, 6, 7. Squamodiscs subequal, wider than peduncle, with 11 or 12 concentric U-shaped rows of rodlets. Ventral anchor with moderately long superficial and deep roots; deep root with lateral swelling; shaft slightly arced; recurved point extending to level of tip of superficial root. Dorsal anchor with subtriangular base, short superficial root, knoblike deep root, arced shaft, and recurved point extending slightly past level of tip of superficial root. Ventral bar with conspicuous medial constriction, tapered ends, longitudinal ventral groove. Paired dorsal bar with spatulate medial end and lateral end with two broadly rounded lobes separated by anteroposterior cleft. Hook with depressed thumb, delicate point, uniform shank; FH loop nearly shank length. Testis subspherical, postgermarial; proximal vas deferens not observed; seminal vesicle a simple dilation of distal vas deferens; anterior duct of seminal vesicle forming a loop before entering thick-walled teardrop-shaped ejaculatory bulb; ejaculatory duct arising from bulb and extending to portal of proximal chamber of MCO; large vesicle (prostatic reservoir?) with clear or reticulate contents lying to right of MCO. MCO reniform, quadriloculate, with short cone; four chambers with delicate walls; retractile filament not observed. Germarium pyriform; germarial bulb pretesticular, with distal loop dorsoventrally around right intestinal cecum; ootype slightly to left of body midline, surrounded by well-developed Mehlis’ gland, giving rise to delicate slightly dilated uterus when empty. Common genital pore ventral, dextral to cone of MCO. Vaginal pore sinistroventral at level of seminal vesicle; vaginal sclerite with distal funnel, sigmoid distal tube frequently recurved distally, and chamber having comparatively thin walls, large cavities, and tubular extension; chamber frequently collapsed in specimens stained with Gomori’s trichrome and mounted in Canada balsam; proximal vaginal canal delicate, extending posteriorly from vaginal sclerite to small subspherical seminal receptacle lying anterior to Mehlis’ gland. Bilateral vitelline ducts not observed; vitellarium absent in regions of other reproductive organs, otherwise dense throughout trunk.

**Figures 145–153.**
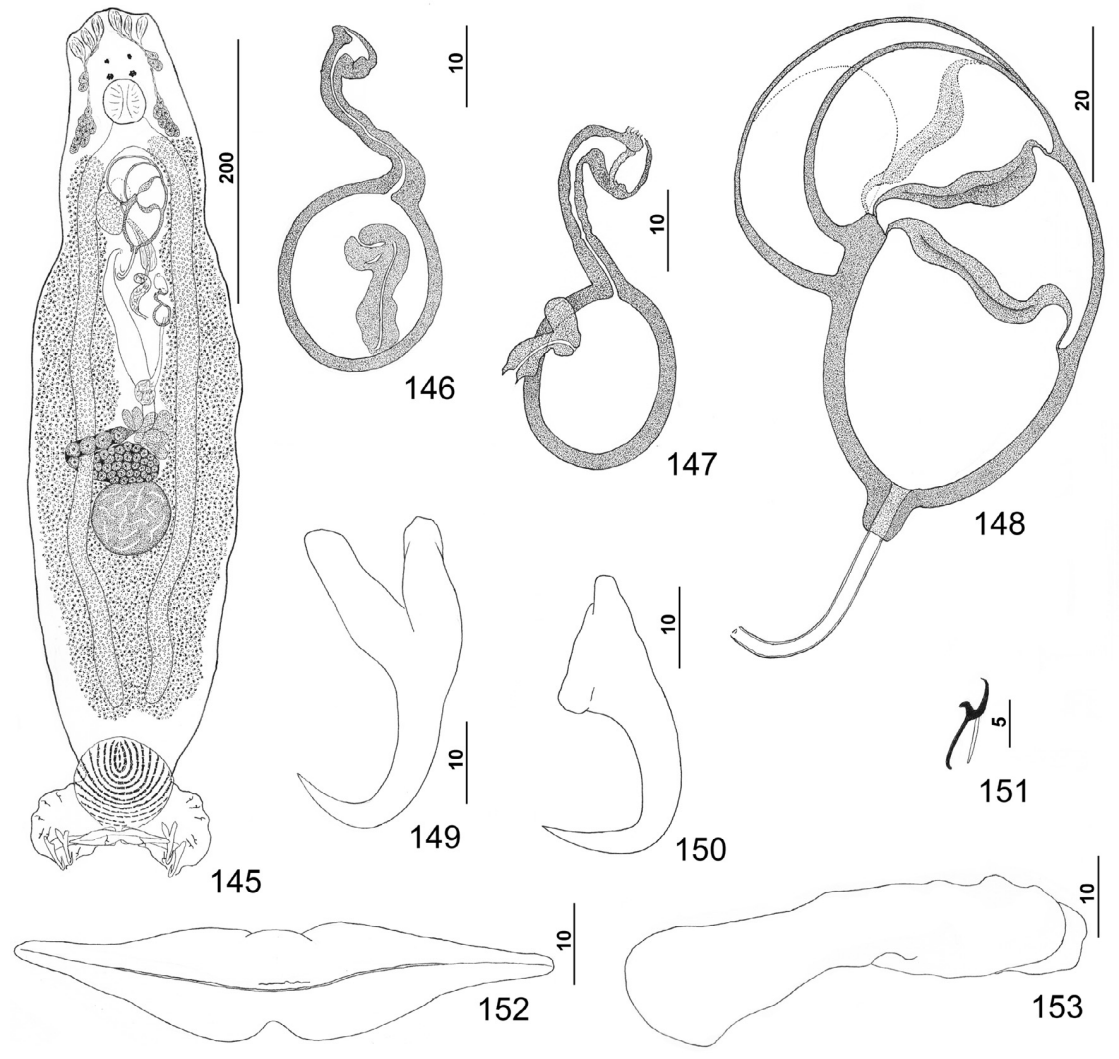
*Pseudorhabdosynochus bunkleywilliamsae* n. sp. from Nassau grouper *Epinephelus striatus*. 145: whole mount (composite, ventral view; dorsal squamodisc and dorsal anteromedial haptoral lobe not shown); 146 and 147: vaginal sclerites (ventral views); 148: male copulatory organ (ventral view); 149: ventral anchor; 150: dorsal anchor; 151: hook; 152: ventral bar; 153: left dorsal bar (ventral view).

Measurements: Body 591 (420–697; *n* = 27) long; width at level of germarium 137 (103–176; *n* = 29). Haptor 147 (119–165; *n* = 27) wide; squamodisc 69 (56–76; *n* = 38) long, 78 (62–92; *n* = 38) wide. Ventral anchor 39 (37–43; *n* = 15) long; dorsal anchor 38 (34–40; *n* = 15) long. Ventral bar 72 (67–82; *n* = 13) long; dorsal bar 59 (54–65; *n* = 14) long. Hook 12 (11–13; *n* = 34) long. Pharynx 32 (26–35; *n* = 22) wide. MCO 70 (62–90; *n* = 27) long. Testis 69 (51–82; *n* = 18) long, 66 (43–81; *n* = 18) wide. Germarial bulb 39 (34–47; *n* = 15) wide.

#### Remarks

*Pseudorhabdosynochus bunkleywilliamsae* n. sp. most closely resembles *P. justinella*, a parasite of *Epinephelus morio*, in the general morphology of the vaginal sclerite and in the shape and number of concentric rows of rodlets in the squamodisc. It differs from *P. justinella* by having ventral anchors with subequal superficial and deep roots (deep root shorter than superficial root in *P. justinella*), and a dorsal bar having a bifid lateral end (lateral end with elongate lobe and not bifurcated in *P. justinella*). While the vaginal sclerites of the two species are very similar, that of *P. justinella* lacks the tubular extension that apparently gives rise to the proximal vaginal canal in *P. bunkleywilliamsae*.

### *Pseudorhabdosynochus woodi* n. sp.

urn:lsid:zoobank.org:act:996D0E93-1EDC-485B-9D19-7CF16EA6AC4C

Syns *Diplectanum epinepheli* of Mizelle & Wood (nomen nudum) (pro parte); *Diplectanum* sp. of USNPC Specimen Log (pro parte).

Host and locality: Red hind, *Epinephelus guttatus* (Linnaeus) (Serranidae: Epinephelinae: Epinephelini): off Castle Roads, Bermuda.

Infection site: Not recorded by Mizelle & Wood (unpublished) (probably gill filaments).

Type specimen: Holotype, USNPC 72747 (M1421-3).

Etymology: This species is named for Dr. Raymond A. Wood, co-discoverer of the species on red hind in Bermuda.

#### Description (Figs. 154–159)

Body flattened dorsoventrally, ovate in dorsoventral view. Tegument smooth, lacking scales. Cephalic region broad, with terminal and two bilateral poorly developed lobes; head organs, cephalic-gland cells not observed. Four eyespots anterior to pharynx lacking lenses; members of posterior pair larger, slightly closer together than those of anterior pair; chromatic granules small, irregular in shape; accessory granules absent in cephalic region. Pharynx subspherical; gut not observed. Peduncle broad. Squamodiscs subequal, with 11 or 12 concentric rows of rodlets; ventral squamodisc with two innermost rows closed; dorsal squamodisc with innermost row closed. Haptor with dorsal and ventral anteromedial lobes containing respective squamodiscs. Ventral anchor with moderately long superficial and deep roots; deep root with lateral swelling, somewhat shorter than superficial root; shaft forming gentle arc; recurved point extending to level of tip of superficial root. Dorsal anchor with subtriangular base, short superficial root, knoblike deep root, arced shaft, and recurved point extending past level of tip of superficial root. Ventral bar robust, with medial constriction, tapered narrow ends, longitudinal ventral groove. Paired dorsal bar with spatulate medial end. Hook with slightly depressed thumb, delicate point, uniform shank; FH loop nearly shank length. Gonads indistinct; soft reproductive ducts and vesicles not observed. MCO damaged by pressure from coverslip, reniform, quadriloculate, with short cone, elongate distal tube; retractile filament not observed. Vaginal sclerite with sigmoid distal tube, small ovate chamber. Vitellarium absent in regions of other reproductive organs, otherwise dense throughout trunk.

**Figures 154–159.**
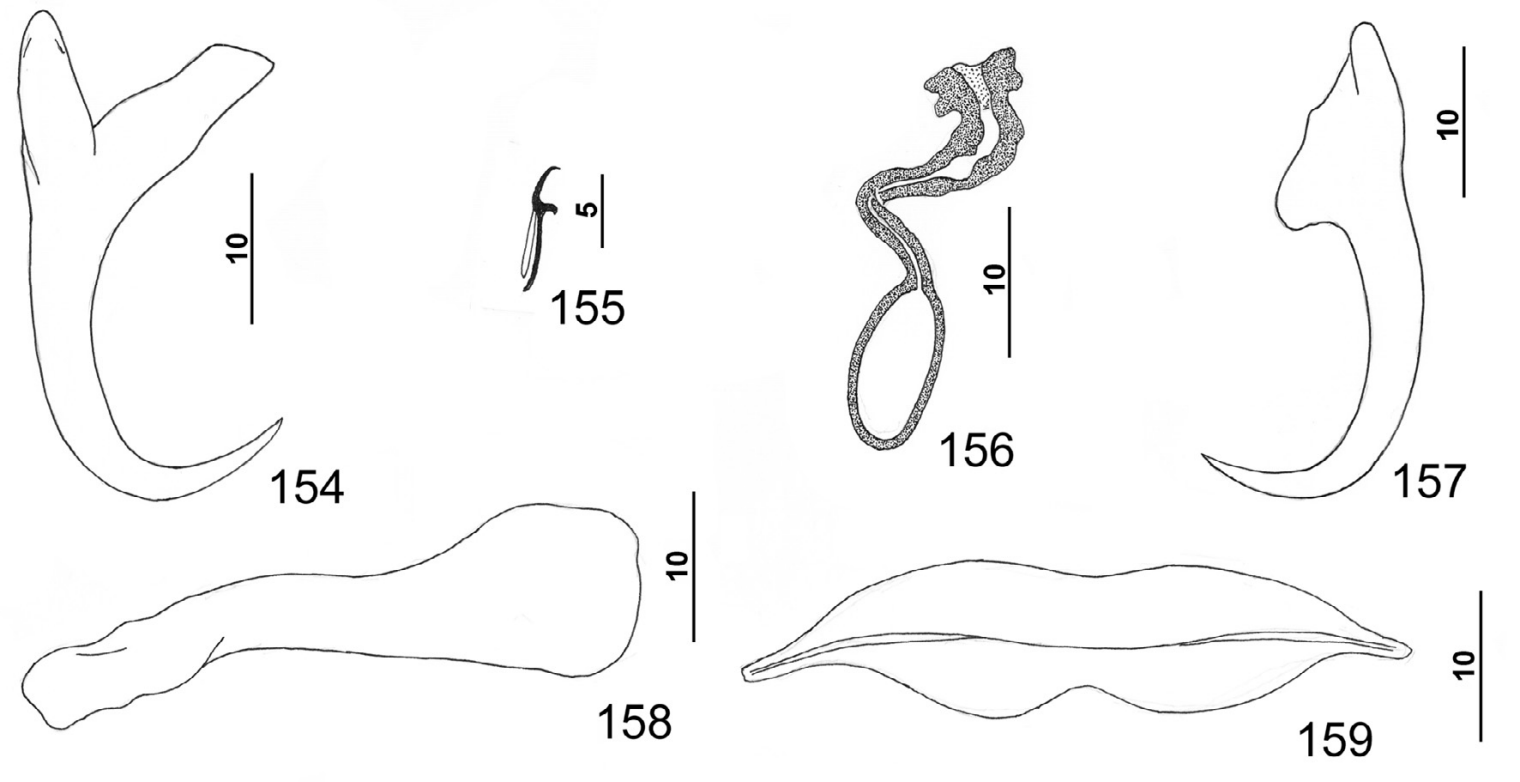
*Pseudorhabdosynochus woodi* n. sp. from red hind *Epinephelus guttatus*. 154: ventral anchor; 155: hook; 156: vaginal sclerite (dorsal view); 157: dorsal anchor; 158: right dorsal bar (ventral view); 159: ventral bar.

Measurements: Body 396–397 (*n* = 1) long; width at level of germarium 131–132 (*n* = 1). Haptor 118–119 (*n* = 1) wide; squamodisc 46–47 (*n* = 1) long, 44–45 (*n* = 1) wide. Ventral anchor 33–34 (*n* = 1) long; dorsal anchor 33–34 (*n* = 1) long. Ventral bar 47–48 (*n* = 1) long; dorsal bar 47–48 (*n* = 1) long. Hook 11–12 (*n* = 1) long.

#### Remarks

*Pseudorhabdosynochus woodi* n. sp. and *P. mizellei* n. sp. were coinhabitants of red hind in Bermuda. *Pseudorhabdosynochus woodi* differs from *P. mizellei* by possessing an ovate chamber in the vaginal sclerite (Fig. 156) (two chambers of *P. mizellei* small and lacking a definitive shape, Fig. 130). It most closely resembles *P. justinella* and *P. bunkleywilliamsae* n. sp. by possessing a short robust ventral bar and a vaginal sclerite having a sigmoid distal tube. The chamber of the vaginal sclerite is small and ovate in *P. woodi* (larger and subspherical in *P. justinella* and *P. bunkleywilliamsae*).

### *Pseudorhabdosynochus tumeovagina* n. sp.

urn:lsid:zoobank.org:act:0ED1DA95-1F1D-4713-9B35-0D2BAC130937

Type host and locality: Speckled hind, *Epinephelus drummondhayi* Goode & Bean (Serranidae: Epinephelinae: Epinephelini) (FSBC 7097): Florida Middle Grounds, Gulf of Mexico (28.453° N, 84.217° W), June 5, 1972.

Infection site: Gill lamellae.

Minimum prevalence: 50% (1 of 2 speckled grouper from Florida infected).

Specimens studied: Holotype, USNM 1273677; 30 paratypes, USNM 1273678, NHMUK 2015.2.25.1–2, MNHN HEL530–531, FSBC-I 127767, 127768.

Etymology: The specific name (a noun) is from Latin (tume/o = to be inflated + vagina) and refers to the bulbous portion of the distal tube of the vaginal sclerite.

#### Description (Figs. 160–167)

Body flattened dorsoventrally, elongate-ovate in dorsoventral view. Tegumental scales not observed (absent?). Cephalic region broad, with terminal and two bilateral poorly developed lobes; three pairs of head organs; pair of bilateral groups of cephalic-gland cells lateral to pharynx. Usually two eyespots anterior to pharynx lacking lenses; chromatic granules small, irregular in outline; accessory granules occasionally absent, few to many usually near eyespots. Pharynx subspherical; esophagus short to nonexistent; intestinal ceca blind, extending in trunk posteriorly from pharynx to posterior to testis. Peduncle broad. Haptor with dorsal and ventral anteromedial lobes containing respective squamodiscs and lateral lobes having hook pairs 2–4, 6, 7. Squamodiscs subequal, with 10–12 (usually 11 or 12) open concentric rows of delicate rodlets. Ventral anchor with short superficial root, deep root with large lateral swelling, short curved shaft, and recurved point extending past level of tip of superficial root. Dorsal anchor with subtriangular base, short roots, slightly curved shaft, and recurved point extending past level of tip of superficial root. Ventral bar with slight medial constriction, tapered ends, longitudinal ventral groove. Paired dorsal bar with expanded medial end. Hook with depressed thumb, delicate point, uniform shank; FH loop about shank length. Testis subspherical to ovate; distal male ducts and vesicles not observed. MCO reniform, quadriloculate, with comparatively long arcing cone; walls of chambers variably thick; distal tube short, with comparatively thick walls; retractile filament variable in length. Germarium pyriform, looping dorsoventrally around right intestinal cecum; Mehlis’ gland not observed; uterus with delicate walls, often collapsed. Vaginal sclerite with distal tube having bulbous expansion, small chamber giving rise to narrow vaginal canal; proximal portion of vaginal canal, seminal receptacle not observed. Vitellarium absent in regions of other reproductive organs, otherwise dense throughout trunk.

**Figures 160–167.**
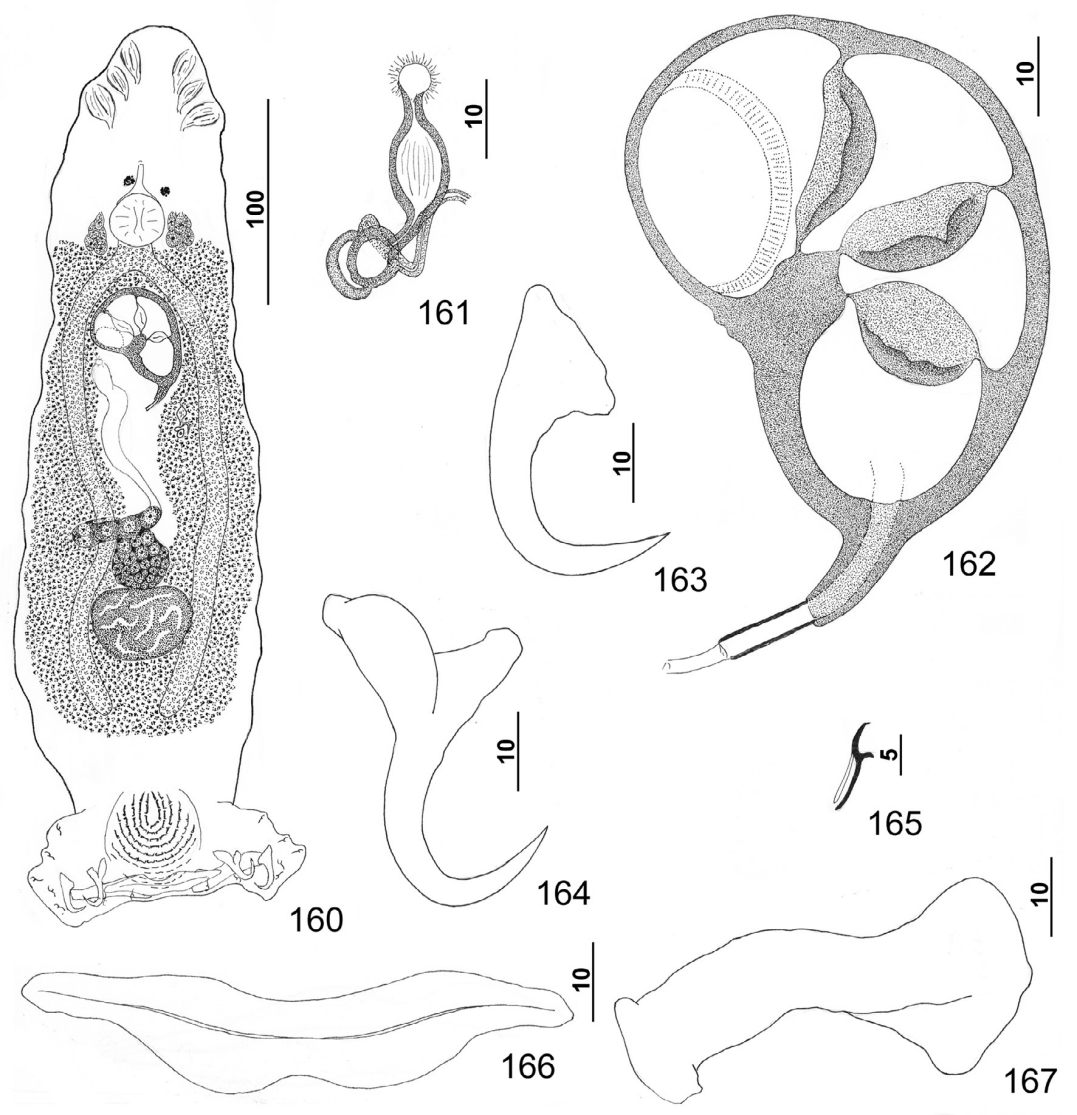
*Pseudorhabdosynochus tumeovagina* n. sp. from speckled hind *Epinephelus drummondhayi*. 160: whole mount (composite, ventral view, dorsal squamodisc and dorsal anteromedial haptoral lobe not shown); 161: vaginal sclerite (dorsal view); 162: male copulatory organ (ventral view); 163: dorsal anchor; 164: ventral anchor; 165: hook; 166: ventral bar; 167: right dorsal bar (ventral view).

Measurements: Body 501 (398–713; *n* = 18) long; width at level of germarium 108 (75–142; *n* = 20). Haptor 136 (115–163; *n* = 14) wide; squamodisc 48 (39–59; *n* = 17) long, 51 (36–60; *n* = 17) wide. Ventral anchor 35 (33–37; *n* = 13) long; dorsal anchor 35 (32–38; *n* = 13) long. Ventral bar 67 (64–70; *n* = 12) long; dorsal bar 48 (43–53; *n* = 13) long. Hook 11–12 (*n* = 11) long. Pharynx 31 (28–35; *n* = 14) wide. MCO 71 (59–87; *n* = 28) long. Testis 38 (30–45; *n* = 6) long, 38 (34–42; *n* = 6) wide. Germarial bulb 27 (18–33; *n* = 11) wide.

#### Remarks

This species is based on specimens obtained from one of two speckled hind examined for gill parasites and held in the FSBC ichthyology collection of the Florida Fish and Wildlife Conservation Commission. The infected speckled hind was collected in 1972 and was not fixed and preserved with its external parasites in mind. As a result, the specimens of *P. tumeovagina* n. sp. from this fish were in generally poor shape, and many of the internal features, particularly those of the male and female reproductive systems, could not be determined. Nonetheless, the unique vaginal sclerite clearly indicated the species to be new to science.

*Pseudorhabdosynochus tumeovagina* n. sp. is differentiated from all previously described species of *Pseudorhabdosynochus* from the region by having an expanded (bulbous) distal tube and a small chamber of the vaginal sclerite. It most closely resembles *P. williamsi* n. sp., by possessing an MCO having an elongate and curved distal cone and comparatively thick-walled chambers. *Pseudorhabdosynochus tumeovagina* differs from *P. williamsi* in the morphology of the distal tube of the vaginal sclerite (bulbous expansion of the distal tube lacking in *P. williamsi*).

### *Pseudorhabdosynochus* sp. 1

Host and locality: Yellowfin grouper, *Mycteroperca venenosa* (Linnaeus) (FSBC 18052): ~12 mi SW of Dry Tortugas National Park, Florida (24.483° N, 82.967° W), March 17, 1980.

Infection site: Gill lamellae.

Minimum Prevalence: 100% (1 yellowfin grouper examined and infected).

Specimen studied: Voucher specimen, USNM 1273684.

#### Remarks

Although the single specimen collected from the yellowfin grouper was strongly contracted and unsatisfactory for species identification, it was easily assigned to *Pseudorhabdosynochus* and recognized as a member of the species group infecting most western Atlantic *Mycteroperca* spp. (see Remarks for *P. kritskyi* for a list of species in the group) based on the general morphology of the MCO and vaginal sclerite. The basic morphology of its dorsal bars and ventral squamodisc, the latter with 16 rows of rodlets (4 innermost rows closed), indicated that the specimen was closest morphologically to *P. capurroi*. However, specific details of the haptoral sclerites, the dorsal squamodisc, the vaginal sclerite, the MCO, and the internal anatomy could not be determined, precluding resolution of the specimen as new or previously described.

### *Pseudorhabdosynochus* sp. 2

Host and locality: Yellowmouth grouper, *Mycteroperca interstitialis* (Poey) (Serranidae: Epinephelinae: Epinephelini) (FSBC 12022): Gulf of Mexico, ~135 mi SW of Galveston, Texas (27°49′59″ N, 93°19′59″ W), March 31, 1981.

Infection site: Gill lamellae.

Minimum Prevalence: 50% (1 of 2 yellowmouth grouper infected).

Specimens studied: 2 voucher specimens, USNM 1273685.

#### Remarks

While the two specimens of *Pseudorhabdosynochus* sp. 2 were unsatisfactory for species identification, they appear to represent an undescribed species. The vaginal sclerite comprises a small chamber associated with a complex of structures situated externally along its proximal wall; the distal tube of the vaginal sclerite was not visible. The MCO had an elongate cone, and the ventral and dorsal squamodiscs had 12 and 10 or 11 concentric rows of rodlets, respectively (two innermost rows closed in both squamodiscs). The single observed egg was bacilliform and lacked filaments. *Pseudorhabdosynochus* sp. 2 appears closest morphologically to *P. mcmichaeli* n. sp. based on the presence of an elongate cone of the MCO and a number of concentric rows of rodlets in the squamodiscs but can be differentiated from *P. mcmichaeli* by the morphology of their vaginal sclerites.

### *Pseudorhabdosynochus* sp. 3

Host and locality: Tiger grouper, *Mycteroperca tigris* (Valenciennes) (Serranidae: Epinephelinae: Epinephelini) (FSBC 11990): ~15 km NNW of Dry Tortugas National Park, Florida (24.533° N, 82.983° W; these coordinates are in the records of the FSBC collection but are not congruent with the written collecting notes accompanying the specimens, the latter of which are considered to be more accurate in this case), April 23, 1980.

Infection site: Gill lamellae.

Minimum Prevalence: 100% (1 specimen from Florida examined and infected).

Specimens studied: 13 voucher specimens, USNM 1273686.

#### Remarks

Specimens of *Pseudorhabdosynochus* sp. 3 were in poor condition, which precluded determination of internal anatomy and specific assignment of the specimens. *Pseudorhabdosynochus* sp. 3 appears closest, if not identical, to *P. mcmichaeli* n. sp., based on the comparative morphology of the MCO, vaginal sclerite, anchors, and ventral bar. In *Pseudorhabdosynochus* sp. 3, the MCO is delicate and has a long depressed cone, an elongate distal tube, and conspicuous filament; the vaginal sclerite has two apparently tandem chambers from which the delicate vaginal duct arises; and the ventral bar and dorsal and ventral anchors are similar to those of *P. mcmichaeli*. The only possibly significant difference observed between the specimens of *Pseudorhabdosynochus* sp. 3 and those of *P. mcmichaeli* is the medial end of the dorsal bar, which in *Pseudorhabdosynochus* sp. 3 is somewhat more spatulate. However, morphology of the dorsal bar is generally not a good feature for species identification because its appearance often changes depending on the rotation of the bar within the plane of view under microscopy.

## Discussion

Species of *Pseudorhabdosynochus* are parasites almost exclusively of groupers (Serranidae) assigned to the tribe Epinephelini. Of the 25 known species of groupers assigned to the tribe and occurring in western Atlantic waters [4, 16], individuals of 23, including those collected during the present study, were examined for species of *Pseudorhabdosynochus*. Prior to the present study, only seven species of *Pseudorhabdosynochus* were documented from seven epinephelin species in the region. Thirteen new species are described herein, resulting in a total of 20 species of *Pseudorhabdosynochus* known to infect the gills of these fishes off eastern North and South America.

This number is likely an underestimate of the total diversity of *Pseudorhabdosynochus* species on groupers in the region given that members of the genus generally exhibit a relatively high host specificity, with most restricted to a single species [48]. Although we found no infections by *Pseudorhabdosynochus* species in groupers belonging to six species (the coney *Cephalopholis fulva*, marbled grouper *Dermatolepis inermis*, Spanish flag *Gonioplectrus hispanus*, misty grouper *Hyporthodus mystacinus*, Atlantic creolefish *Paranthias furcifer*, and mutton hamlet *Alphestes afer*), our sample sizes for these potential hosts were small (generally < 5 specimens per species) and all representative host specimens were drawn from the shelves of the FSBC where fixation and sampling methods could easily have led to loss of parasite specimens. An additional two species of epinephelin groupers from the region [the comb grouper *Mycteroperca acutirostris* (Valenciennes) and the Venezuelan grouper *Mycteroperca cidi* (Cervigón)] have yet to be examined. In the present study, none of the 19 identified species of *Pseudorhabdosynochus* crossed host generic boundaries in host preference, and only two occurred on more than one congeneric host: *P. firmicoleatus* on *Hyporthodus flavolimbatus* and *H. niveatus*; and *P. sulamericanus* on *H. niveatus* and *H. nigritus*.

Recent studies on parasites of other fishes off the eastern coasts of North and South America further demonstrate that the overall monogenoidean fauna of the region is larger than previously recorded. In what are primarily focused studies dealing with specific host groups or individual species of fish in the region, new species of Monogenoidea have frequently been described [21, 24, 27, 43, 47, among others]. The monogenoidean fauna off western North and South America is even less documented, although a few studies, in which new species of Monogenoidea are described, have recently been published [15, 29, 30, 31, 41, 60, and others].

Based on molecular data, Craig & Hastings [4] revised the Epinephelini to include 11 genera, of which representatives of seven, comprising all epinephelin genera with species in the western Atlantic region, were examined for *Pseudorhabdosynochus* species during the present study. While phylogenetic analyses are lacking for this group of helminths, comparative morphology and the occurrence of *Pseudorhabdosynochus* species on these hosts suggest that concomitant speciation among these helminths occurred at least in part with that of their hosts. For example, the six host species assigned to *Mycteroperca* by Craig & Hastings [4] and examined during the present study are parasitized by morphologically similar species (*P. kritskyi, P. capurroi, P. vascellum, P. contubernalis*, *P. hyphessometochus*, *P. mycteropercae*, and *Pseudorhabdosynochus* sp. 1), suggesting that coevolution is a likely component of the evolutionary histories of the respective host/parasite relationships. Similarly, species of *Hyporthodus* are parasitized by identical or similar species of *Pseudorhabdosynochus* (*P. sulamericanus* and *P. firmicoleatus*), while *P. yucatanensis, P. bunkleywilliamsae*, and *P. woodi*, all parasites of *Epinephelus* spp., also may share a common ancestor based on the comparative morphology of their respective vaginal sclerites.

## A note on the type species of *Pseudorhabdosynochus* Yamaguti, 1958

Kritsky & Beverley-Burton [25] discussed the concept of the type species of a genus sensu the International Code of Zoological Nomenclature (ICZN), in regard to that of *Pseudorhabdosynochus*. Based on the ICZN, these authors indicated that type species was a nomenclatural concept and not one of taxonomy or biology. As a result, Kritsky & Beverley-Burton [25] stated that the type species of *Pseudorhabdosynochus* was *P. epinepheli* Yamaguti, 1958, which was a junior subjective synonym of *P. epinepheli* (Yamaguti, 1938) Kritsky & Beverley-Burton, 1986. According to the ICZN, the placement of *P. epinepheli* Yamaguti, 1958 as a junior synonym does not invalidate the nominal species as the type species of *Pseudorhabdosynochus*. Unfortunately, some recent publications [20, 46] erroneously stated that the type species of the genus was *P. epinepheli* (Yamaguti, 1938) Kritsky & Beverley-Burton, 1986, apparently because it was the senior synonym of the “taxonomic” species. Indeed, Justine [20] included the error in the title of his paper.

Subjective synonymy of the type species of a genus is not an unusual occurrence. An example comparable to that described above for *Pseudorhabdosynochus* was found within the genera comprising the host group on which the present study is based. *Hyporthodus* Gill was recently resurrected for 14 grouper species previously assigned to other epinephelin genera [4]. *Hyporthodus* was originally proposed by Gill [14], who by monotypy assigned *Hyporthodus flavicauda* Gill, 1861 as its type species. *Hyporthodus flavicauda* was listed as a subjective junior synonym of *Epinephelus niveatus* by Heemstra & Randall [16]. Craig & Hastings [4] accepted the junior subjective synonymy of *H. flavicauda* with *E. niveatus* (now *H. niveatus*), while rightfully recognizing *H. flavicauda* as the type species when resurrecting *Hyporthodus*. Information on the nomenclatural and taxonomic history of *Hyporthodus* and its type species is available in Eschmeyer & Fong [8], who concurred with the actions of Heemstra & Randall [16] and Craig & Hastings [4].
